# A COVID-19 Drug Repurposing Strategy through Quantitative Homological Similarities Using a Topological Data Analysis-Based Framework

**DOI:** 10.3390/pharmaceutics13040488

**Published:** 2021-04-02

**Authors:** Raul Pérez-Moraga, Jaume Forés-Martos, Beatriz Suay-García, Jean-Louis Duval, Antonio Falcó, Joan Climent

**Affiliations:** 1 ESI International Chair@CEU-UCH, Universidad Cardenal Herrera-CEU, CEU Universities, San Bartolomé 55, Alfara del Patriarca, 46115 Valencia, Spain; raulcl1994@gmail.com (R.P.-M.); fores.martos.jaume@gmail.com (J.F.-M.); beatriz.suay@uchceu.es (B.S.-G.); 2 Departamento de Matemáticas, Física y Ciencias Tecnológicas, Universidad Cardenal Herrera-CEU, CEU Universities, San Bartolomé 55, Alfara del Patriarca, 46115 Valencia, Spain; 3 Biomedical Research Networking Center of Mental Health (CIBERSAM), 28029 Madrid, Spain; 4 ESI Group, 3bis rue Saarinen, 94528 Rungis, France; Jean-Louis.Duval@esi-group.com; 5 Departamento de Producción y Sanidad Animal, Salud Pública Veterinaria y Ciencia y Tecnología de los Alimentos, Universidad Cardenal Herrera-CEU, CEU Universities, C/Tirant lo Blanc 7, Alfara del Patriarca, 46115 Valencia, Spain

**Keywords:** COVID-19, drug repurposing, topological data analysis, persistent Betti function

## Abstract

Since its emergence in March 2020, the SARS-CoV-2 global pandemic has produced more than 116 million cases and 2.5 million deaths worldwide. Despite the enormous efforts carried out by the scientific community, no effective treatments have been developed to date. We applied a novel computational pipeline aimed to accelerate the process of identifying drug repurposing candidates which allows us to compare three-dimensional protein structures. Its use in conjunction with two in silico validation strategies (molecular docking and transcriptomic analyses) allowed us to identify a set of potential drug repurposing candidates targeting three viral proteins (3CL viral protease, NSP15 endoribonuclease, and NSP12 RNA-dependent RNA polymerase), which included rutin, dexamethasone, and vemurafenib. This is the first time that a topological data analysis (TDA)-based strategy has been used to compare a massive number of protein structures with the final objective of performing drug repurposing to treat SARS-CoV-2 infection.

## 1. Introduction

On 11 March 2020, the World Health Organization (WHO) declared the Coronavirus Disease 2019 (COVID-19) outbreak, produced by the novel SARS-CoV-2 virus, a global pandemic [1]. To date, three previously approved antiviral drugs and one antimalarial medication (remdesevir, iopinavir, interferon-1, and hydroxychloroquine) have been tested for efficacy against SARS-CoV-2 infection by the WHO SOLIDARITY consortium in a large multicentric study. The results of the trial suggested that these treatments had little or no effect in a set of clinical outcomes which included overall mortality, time to initiation of mechanical ventilation, and duration of hospital stay [2].

With the third wave ongoing in many countries, herd immunity a distant prospect, and new strains challenging the existing vaccines, it is still a pressing need to find adequate treatments for the disease. De novo drug development and testing, including preclinical research and clinical trials, is a slow process that could take more than 12 years [3,4]. However, the current sanitary emergency makes it imperative to shorten this time frame. Therefore, sustained efforts to identify potential candidates for drug repurposing are necessary.

In the context of COVID-19, Kumar and co-workers compiled sets of genes linked to the disorder and studied their distribution in the human interactome [5]. They first identified the interactome subnetworks’ hub genes in which the disease-related genes were placed. Then, they queried the drug–gene interaction database to identify Food and Drug Administration (FDA)-approved drugs that had the hub genes as their target (i.e., chloroquine, lenalidomide, pentoxifylline) [6,7]. Zhou and collaborators compiled a list of human proteins that physically interact with four previous human coronaviruses (SARS-CoV, MERS-CoV, HCoV-229E, and HCoV-NL63) and used network proximity measures to prioritize 16 potential anti-human coronavirus repurposable drugs including melatonin, mercaptopurine, and sirolimus [8]. Drug repurposing studies using virtual screening procedures based on molecular docking have also been reported. To cite an example, Kerestsu et al. used a protease inhibitors database (MEROSP) and the geometric structure of the 3C-Like virus protease (3CLpro) to identify 15 potential inhibitors using the surflex-Dock software [9].

**Figure 1:**
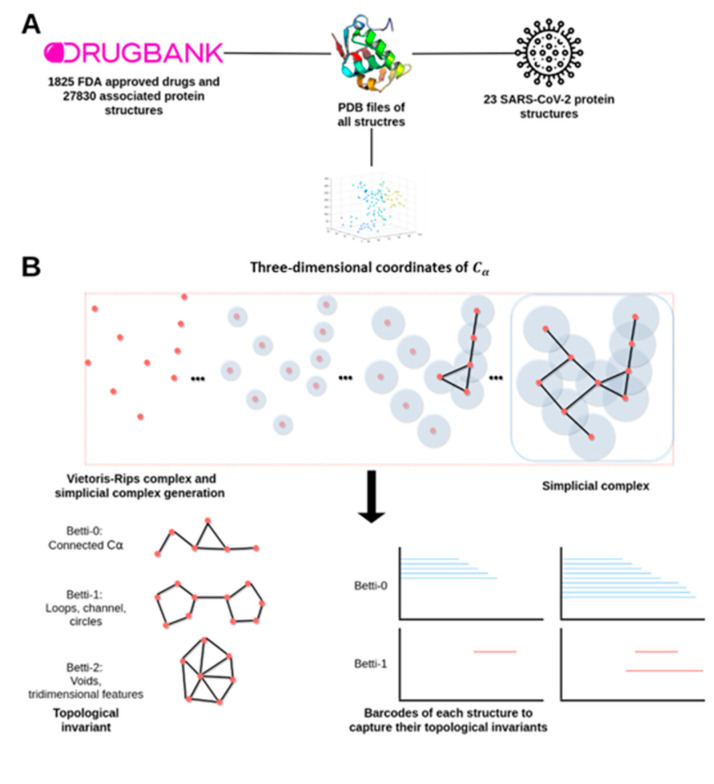

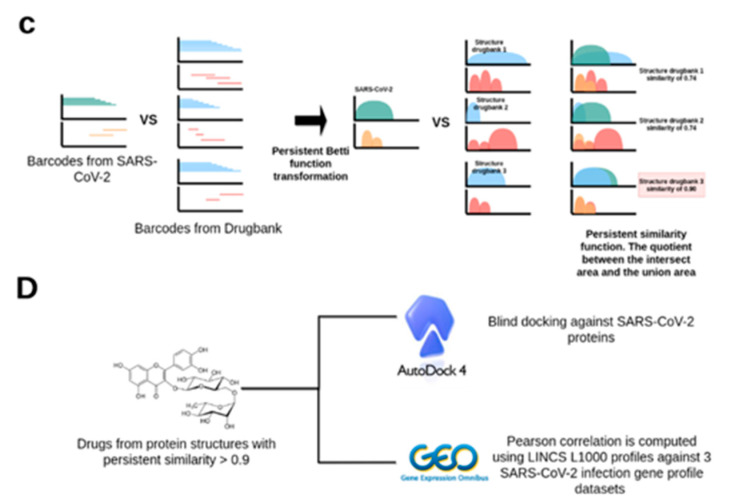
Bioinformatic work-flow used. (**A**) Data preprocessing and acquisition (**B**) Topological data analysis phase, Vietoris–Rips complexes at scale ε are computed to generate the barcodes. Each ε-associated Betti number captures a unique topological feature of the protein. (**C**) To compare barcodes of viral proteins against structures with known drugs, it is necessary to transform barcodes into comparable curves using persistent Betti functions (PBFs). (**D**) Candidate drugs from proteins with a mean persistent similarity score above 0.9 were validated by a dual in silico strategy. We used AutoDock 4 to analyze the capacity of the drug to bind against viral proteins. Transcriptomics analysis was performed to test the capacity of the candidate drugs to revert the transcriptomics effect induced by the COVID-19.

Here, we present a general-purpose drug repositioning workflow and its application to the specific case of COVID-19. Our procedure is based on recent developments in the field of topological data analysis (TDA) and its use in the study of biological geometric structures [10]. In particular, our method relies on the idea that drugs that are known to target a specific protein would likely target other proteins that present high degrees of topological similarities with the initial protein. Therefore, the accumulated knowledge of drug–protein interactions available in public repositories such as DrugBank in combination with the information about protein three-dimensional structures found in the Protein Data Bank (PDB) can be used to predict new potential drug protein targets based on the computation of protein–protein topological similarities. Figure 1 contains a brief summary of the general methodology.

## 2. Results

### 2.1. Drugs, Protein Targets, and PDB Structures Included in This Study

DrugBank queries yielded 1825 drugs approved by the American Food and Drug Administration (FDA). The identified drugs had 1821 known unique protein targets, for which 27,839 three-dimensional structures were available in the protein databank. The first three persistent Betti functions (PBFs, see Section 4.2) were successfully calculated for 25,800 of the 27,839 structures, whereas computational limitations prevented us from estimating the remaining 1622 structures’ PBFs. We also retrieved multiple protein structures from SARS-CoV-2 that were available in PDB, including the Spike protein receptor binding domain, the RNA-dependent RNA polymerase (NSP12), the endoribonuclease (NSP15), the ADP ribose phosphatase (NSP3), the RNA binding protein (NSP9), the 3C-like protease, and the NSP 8 and 7. In total, we calculated the PBFs of 23 viral protein structures. Table 1 shows the complete information regarding the included SARS-CoV-2 protein structures.

### 2.2. TDA Results, Viral Proteins Showing Mean Persistent Similarities above 0.9 with Structures Targeted by Known FDA-Approved Drugs

We compared 23 PDB structures derived from SARS-CoV-2 with 25,800 structures belonging to proteins that are known targets of FDA-approved drugs through the computation of 593,400 persistent similarity measures. We selected a stringent threshold of 0.9 for the mean of the persistent similarity measures (see Section 4.2) in order to call two protein structures similar. Three viral structures, the 3CL protease (6M2Q), the RNA-dependent RNA polymerase (6M71), and the NSP15 endoribonuclease (6W01), presented a mean of the persistent similarity measures with values higher than the selected threshold with proteins known to be targeted by approved drugs. The 3CL protease was found to be associated with 284 PDB structures (Supplementary Table S1), most of them classified as Aldo/Keto reductases and protein kinases, which were targeted by 55 different pharmacological compounds (Supplementary Table S2). The RNA-dependent RNA polymerase was found to be significantly associated with 361 PDB structures (Supplementary Table S3), which in many cases belonged to the protein kinase and flavin-containing oxidoreductase families, and that were found to be targeted by 204 unique drugs (Supplementary Table S4). Finally, the viral NSP15 endoribonuclease presented topological similarity values higher than 0.9 with 13 PDB structures (Supplementary Table S5), where the most abundant group was the poly(Adp-RIbose) Polymerase Catalytic Domain. These structures were targeted by 45 drugs (Supplementary Table S6).

**Figure 2:**
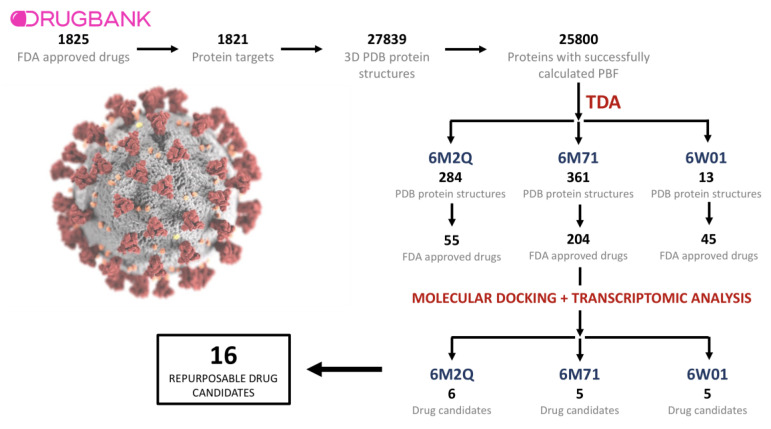
Schematization of the narrowing-down process followed to identify the final 16 drug candidates.

Drugs known to target proteins presenting a mean of the persistent similarity measures larger than 0.9 with the SARS-CoV-2 structures were subjected to blind docking with the viral proteins. Blind docking was carried out using the complete viral protein and drug structure information preprocessed as detailed in Section 4, which included polar hydrogen addition. A set of potential repurposable candidates was then selected based on the topological similarity criteria (a mean of the persistent similarity measures), the correlations between the transcriptomic profiles observed in patients infected by SARS-CoV-2 and those generated by treating cell lines with the candidate drugs, and the blind docking analyses results. Therefore, the selected candidates are known to target proteins with large topological similarities with a specific viral protein, present high affinities with the viral structures, and have the capacity to partially revert the transcriptomic effects induced by the viral infection. Figure 2 provides a schematic overview of the narrowing-down process followed to identify the final 16 drug candidates. Furthermore, the full description of the candidates can be consulted in Table 2.

We identified six repurposable candidates to target the 3CL viral protease (6M2Q). Cholic acid, an amphipathic sterol, presented the strongest binding energies (BE = −15.06 kcal/mol), and was found to negatively correlate with transcriptomic dataset 2 (DS2 r = −0.11). Rutin (BE = −14.52 kcal/mol, DS2 r = −0.184 DS3 r = −0.1), a flavonoid-3-o-glycoside with known antioxidant and cytoprotective activity, was also selected [11,12]. Two non-steroidal anti-inflammatory drugs, indomethacin (BE = −13.31 kcal/mol, DS2 r = −0.12) and sulindac (BE = −13.14 kcal/mol, DS2 r = −0.12), were also identified. Whereas indomethacin presents antipyretic and analgesic properties [13], sulindac is used to treat conditions that involve chronic inflammation, such as arthritis [14]. Finally, sulfisoxazole (BE = −11.59 kcal/mol DS2 r = −0.13), a sulfanilamide used as a broad-spectrum antibiotic, and dasatinib (BE = −10.94 kcal/mol DS2 r = −0.15), a tyrosine kinase inhibitor indicated for the treatment of chronic myeloid leukaemia [15], were also identified as drugs with the potential of targeting the viral 3CL protease.

Five compounds were found to be candidates to target the SARS-CoV-2 NSP15 endoribonuclease (6W01), which included two corticosteroids, dexamethasone (BE = −11.42 kcal/mol, DS2 r = −0.15) and spironolactone (BE = −10.99 kcal/mol, DS1 r = −0.12 and DS2 r = −0.1), which are indicated for the treatment of allergies and asthma and resistant hypertension, respectively [14,16,17]; phenolphthalein (BE = −11.15 kcal/mol, DS1 r = −0.13), a compound historically used as a laxative [18]; mifepristone (BE = −10.04 kcal/mol, DS1 r = −0.13, DS2 r = −0.14), a synthetic steroid progesterone antagonist drug that is indicated for Cushing’s syndrome and is also used as an emergency contraceptive pill [19,20]; and, finally, carbamazepine (BE = −9.66 kcal/mol, DS2 r = −0.15), a pharmacologically active molecule related to the group of tricyclic antidepressants, mainly used as anticonvulsant [14,21].

Lastly, the analysis of the NSP12 RNA-dependent RNA polymerase (6M71) yielded multiple antineoplastic drugs as possible repurposing candidates: vemurafenib (BE = −8.09 kcal/mol DS2 r = −0.16), a BRAF inhibitor [22,23]; sorafenib (BE = −7.34 kcal/mol DS1 r = −0.11, DS2 r = −0.15), a multitarget protein kinase inhibitor [24]; levonorgestrel (BE = −7,21 kcal/mol, DS2 r = −0.14), a synthetic progestogen used as a first-line oral emergency contractive pill [14]; the opioid antagonist naloxone (BE = −7.07 kcal/mol, DS2 r = −0.11); and raloxifene (BE = −7.05 kcal/mol, DS1 r = −0.13 and DS2 r = −0.17), a selective estrogen receptor modulator mainly used to treat osteoporosis in postmenopausal women and avoid bone loss [25]. Supplementary File 2 shows the interacting residues between the three viral proteins and the 16 drugs identified as potential repurposing candidates.

### 2.3. Transcriptomic Data Analysis Results

Differential gene expression analyses were carried out with the three identified datasets including samples infected with SARS-CoV-2 and uninfected controls, and were followed by Gene Set Enrichment Analysis (GSEA) and LINCS L1000 analysis. GSEA analyses allow the identification of coordinated changes in the expression of genes belonging to specific biological processes and pathways in case samples compared to controls. GSEA results are reported using the Normalized Enrichment Score (NES) and the *p*-value adjusted by multiple comparisons (p-adj). LINCS L1000 analyses aim to find drugs capable of reverting the transcriptomic effects produced by SARS-CoV-2 infection. Differential gene expression analysis of DS1 yielded 451 deregulated genes (DEGs), of which 213 were found to be upregulated and 238 were downregulated in SARS-CoV-2 infected samples compared to controls. The top upregulated genes were derived from the virus open reading frames. Gene Set Enrichment Analysis (GSEA) showed that pathways linked to the immune response were heavily upregulated in SARSCoV-2-infected samples. Instances of such pathways included immune response mediated by circulating immunoglobulin (p-adj = 1.8 × 10^−25^), B-cell mediated immunity, (p-adj = 3.2 × 10^−22^), and adaptive immune response (p-adj = 2.0 × 10^−20^). The FDA-approved drugs showing the strongest negative correlation in LINCS L1000 analysis were niclosamide, bisacodyl, and perhexiline (r = −0.21, −0.19, −0.18, respectively). GSEA analysis of the transcriptomic signatures produced by these medications suggested that they induce significant gene expression changes in pathways linked to interleukin signaling and NF-kB activation. Genes included in the set of potential 105 therapeutics for SARS were also found to be upregulated in the bisacodyl signature (NES = 1.61, p-adj = 2.19 × 10^−2^). The JAK-STAT complex and the TCF-dependent signaling pathways were found to be downregulated in the perhexiline and niclosamide signatures, respectively.

A total of 8380 DEGs were identified in the DS2 analysis. A total of 4606 genes were found to be upregulated, and 3774 were found to be downregulated in SARS CoV-2 infected samples compared to uninfected controls. Upregulated genes were enriched in components of the humoral immune response, epidermis development, keratinization, and B-cell-mediated immunity (p-adj = 1.1 × 10^−20^, 8.2 × 10^−20^, 1.3 × 10^−18^, 2.5 × 10^−10^, respectively), among others. The top negatively correlated drugs included instances of several different compound families, such as anti-inflammatories (phenylbutazone, r = −0.21), antidiabetics (troglitazone, r = −0.20), antimalarials (chloroquine, r = −0.20), and other compounds such as nicotine (r = −0.17). Treatment with phenylbutazone was found to upregulate the gene expression of genes included in the interleukin-12 and 17 signaling pathways. In contrast, interleukin-4 and 13 signaling-related genes tended to be downregulated by chloroquine treatment (NES = −1.45, p-adj = 4.30 × 10^−2^). Genes involved in the viral mRNA translation and the ISG15 antiviral mechanism were also upregulated in the gene expression profiles induced by treatment with chloroquine, phenylbutazone, and troglitazone. In addition, the SARS-CoV infection pathway was found to be upregulated in samples treated by chloroquine and troglitazone. ADORA2B-mediated anti-inflammatory cytokine production-related genes were downregulated by the treatment of the three top negatively correlated drugs.

DS3 presented the lowest yield in terms of differentially expressed genes. A total of 188 genes were found to be upregulated to controls, whereas 31 genes were found to be downregulated in infected samples compared to controls. Twenty-nine biological processes were found to be significantly upregulated and were mainly linked to mechanisms aimed to fight the viral infection and immune system-related processes including, defense response to virus (p-adj = 7.2 × 10^−13^), myeloid leukocyte-mediated immunity (p-adj = 8.8 × 10^−15^), regulation of cytokine production (p-adj = 1.5 × 10^−8^), and response to interferon-gamma (p-adj = 1.9 × 10^−8^), among others. Chloroquine was found to be the top negatively correlated drug (r = −0.11), followed by others such as pazopanib, spectinomycin, and troglitazone (r = −0.11, −0.11, −0.10, respectively). The correlations observed in this dataset tended to be weaker than those computed for DS1 and DS2. GSEA analyses of the drug signatures showed that troglitazone increased the expression of genes classified as potential therapeutics for SARS (NES = 1.46, p-adj = 4.65 × 10^−2^), in addition to antiviral pathways such as the ISG15 and IFN-stimulated antiviral mechanisms. Spectinomycin was found to reduce the expression of interferon-gamma signaling 135 and interleukin 2, 3, and 5 pathway-related genes, whereas pazopanib was found to upregulate viral-related pathways such as viral mRNA translation influenza and SARS-CoV-2 infection. Supplementary File 1 includes the complete differential gene expression and enrichment analysis results for transcriptomic datasets 1, 2, and 3, whereas Supplementary File 2 contains the full LINCS L1000 analysis information.

### 2.4. GSEA Analysis of the Repurposing Candidates

**Figure 3:**
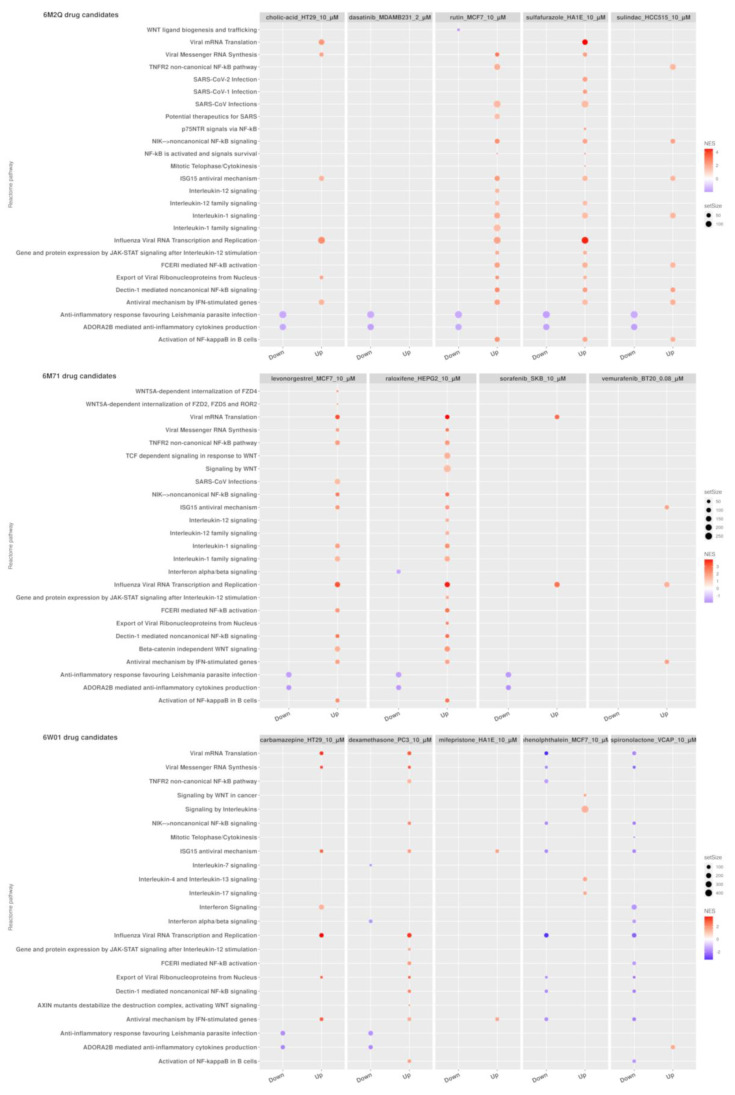
Gene Set Enrichment Analysis (GSEA) results for candidate drugs for 6M2Q, 6M71, and 6W01 SARS-CoV-2 structures with the expression signature yields from correlation analyses from DS2. Reactome pathways related to the immune system and viral infections. Only drugs with at least one pathway with an adjusted *p*-value < 0.05 are displayed. The GSEA table with the results is available in Supplementary Tables S7–S9.

We determined the transcriptomic impact of the treatment with the selected candidates on two sets of biological processes linked to COVID-19, viral infections, and immune-related pathways by performing Gene Set Enrichment Analysis (GSEA) of their gene expression signatures derived from LINCS L1000. The transcriptomic profiles generated by cholic acid, rutin, sulfafurazole, and sulindac treatment (candidates to target the 3CL protease) were found to be enriched in the ISG15 antiviral mechanism. Furthermore, genes related to interleukin-1 and 12 signaling tended to be upregulated in rutin’s signature, in addition to genes belonging to the potential therapeutics for SARS gene set (NES = 1.51, p-adj = 3.85 × 10^−2^) whereas WNT ligand biogenesis and trafficking (NES) genes were found to be downregulated by rutin treatment (NES = −1.99, p-adj = 2.12 × 10^−3^) (Supplementary Table S7). RNA-dependent RNA polymerase drug candidates, levonorgestrel and raloxifene, were found to be enriched in pathways related to antiviral processes such as ISG15 antiviral mechanism (levonorgestrel, NES = 2.08, p-adj = 9.95 × 10^−4^; raloxifene, NES = 2.06, p-adj = 8.13 × 10^−4^) and antiviral mechanism by IFN-stimulated genes (levonorgestrel, NES = 1.95, p-adj = 1.22 × 10^−3^; raloxifene, NES = 1.94, p-adj = 1.12 × 10^−3^). In addition, interferon alpha/beta signaling was observed to be depleted in raloxifene-treated cells (NES = −1.52, p-adj = 4.59 × 10^−2^) (Supplementary Table S8). Finally, in the case of NSP15 endoribonuclease candidate drugs, dexamethasone produced gene expression signatures upregulated in pathways associated with viral infection response, such as ISG15 antiviral mechanism (NES = 1.82, p-adj = 3.17 × 10^−3^) and the antiviral mechanism by IFN-stimulated genes (NES = 1.59, p-adj = 1.20 × 10^−2^). This pathway was also found to be upregulated in the gene expression profiles of carbamazepine and mifepristone. Finally, interleukin-7 signaling (NES = −1.64, p-adj = 3.47 × 10^−2^) and interferon alpha/beta signaling (NES = −1.68, p-adj = 5.48 × 10^−3^) were downregulated by dexamethasone treatment (Supplementary Table S9). Figure 3 shows a dot plot representation of the GSEA analysis results. 

## 3. Discussion

On December 31st, 2019, the World Health Organization (WHO) was officially notified about several cases of pneumonia in Wuhan City, China, caused by COVID-19, a disease with no effective treatment nor a specific vaccine at that time, which history and quest for a cure is a daily struggle and is constantly being rewritten. As specific antiviral treatments are still under development and the vaccination campaign has faced difficulties derived from unmet forecasts in the process of production and distribution, drug repurposing strategies suggesting the use of FDA-approved drugs continue to be a valuable option to find candidate drugs for the effective treatment of COVID-19 in a short timeframe. 

Here, we report a novel TDA-based strategy for drug repurposing in combination with current methodologies of molecular docking, differential expression analysis of SARS-CoV-2 infected cells, and correlation with FDA-approved drugs transcriptomic profiles. Our results indicate that the proposed TDA-based formalism is a promising tool to address biological problems from a dual perspective. First, from a structural biology perspective, we used the Vietoris–Rips complex to compute the PBF encoding the shape of each protein structure. Next, to measure the degree of similarity between proteins we introduced the persistent similarity measure (PSM, see Section 4.2). This allowed us to classify proteins based solely on the C atomic coordinates. TDA-based methods have been previously proposed as a method to study the topological invariants of the three-dimensional structure of biomolecules. Several studies have employed this framework to classify protein structures using only the three-dimensional coordinates of the atoms from crystallographically resolved proteins. For instance, Xia and collaborators performed TDA-based methods on three-dimensional biomolecular structures to study their structural characteristics, flexibility prediction, and folding properties [10]. Hence, they defined the molecular topological fingerprints (MFTs) to extract the topological information from protein structures using the so-called persistent Betti numbers [26]. K. Dey and colleagues proposed another topology-based method to generate protein signatures to create a fast domain classifier using a support vector machine [27]. Interestingly, our mean persistence similarity metric was able to achieve results comparable to those obtained by the state-of-the-art structural alignment method, DALI [28], and presented a high predictive power clustering protein in terms of external classifications.

Molecular docking simulation is a rapid screening method to test compound binding activity. Additionally, transcriptomic data represent a rich alternative resource for inferring non-obvious relationships between drugs and genes. Previous in silico molecular docking studies have highlighted the potential of repurposed drugs for the treatment of COVID-19 [29,30,31,32,33,34,35]. However, here we used in silico molecular docking combined with transcriptomic small molecule treatment data from LINCS L1000 to determine which FDA-approved drugs may reverse the effects of SARS-CoV-2 infection. The gene expression profiles in response to the identified drugs support the docking results and offer a plausible perspective for the pathways associated with protein responses to drugs binding to SARS-CoV-2 proteins. To our knowledge, this is the first time that an application of barcode-based similarity measures has been used for the analysis of large datasets of PDB structures.

The generation of PBF depends upon the previous construction of Vietoris–Rips complexes, which have a computational store cost that scales exponentially with the number of points defining a particular structure. Moreover, in the worst case, the standard algorithm to compute the barcodes has cubic complexity in the number of simplices. Although our analyses were carried out in a cluster with 32 cores and up to 500 GB of RAM, the computational cost of the barcode generation of the excluded 1622 genes exceeded the available amount of RAM or required an exponential amount of runtime.

Among all of the SARS-Cov-2 proteins analyzed (*n* = 23, Table 1), only three showed a persistent similarity score above 0.9 against other protein structures targeted with known drugs. Interestingly, these proteins are key components in coronavirus replication and structural assembly: the Viral 3CL protease (6M2Q), a chymotrypsin-like protease that is essential for the production of non-structural proteins [36]; the nsp12 RNA-dependent RNA polymerase (6M71), the main component of coronavirus replication and transcription machinery, and because of that an excellent target for new therapeutics [37]; and the nsp15 endoribonuclease (6W01), a protein with a poorly defined role in SARS-CoV-2 infection, but which has been described to be linked to pRB downregulation affecting host cell cycle division and coronavirus infection in other coronaviruses (SARS-CoV), and with a role as an antagonist of host dsRNA sensors during coronavirus infection in macrophages to evade innate immune system defenses [38,39]. Hence, in this study, we selected three proteins from the SARS-CoV-2 coronavirus as the best candidates to find repurposed drugs to combat the disease.

Our differential expression analyses revealed that troglitazone, niclosamide, and chloroquine, among multiple candidates, were the top negatively correlated drugs that may revert the effects of SARS-CoV-2 infection to the cell transcriptome. Moreover, chloroquine is already under study in several clinical trials, although recent results reported by the WHO SOLIDARITY study stated that chloroquine has no significant effect on hospitalized COVID-19 patients, in terms of the overall mortality level [2]. Niclosamide is also being evaluated under a Phase 2 clinical trial [40]. In addition, the antiviral activity of the niclosamide has been demonstrated against SARS-CoV in in vitro studies [41] and recent investigations against SARS-CoV-2 [42], and also previously against other MERS coronaviruses [43].

To date, no therapeutic agents have been proven to be effective against SARS-CoV-2. Several treatments have been reported under investigation specifically to treat COVID-19 as the result of drug repurposing strategies [44,45] and, as this draft is being written, up to 700 research papers have already been published. The number of clinical trials using repurposed drugs such as hydroxychloroquine, remdesivir, and lopinavir/ritonavir, among others, alone or in combination, is also exponentially growing, although in most cases unfortunately the results are not as good as initially expected [46,47,48]. Recently, a new treatment, plitidepsin, has been reported as the most potent antiviral drug against the coronavirus [49].

Our more promising candidates arise from the combination of molecular docking and transcriptomic results, and the cornerstone of our work, the TDA-based formalism. Among the 16 compounds related to the three SARS-CoV-2 proteins analyzed, nine have been described as possible candidates in other repurposing studies and five of these have already shown antiviral activity or have already been described as possible COVID-19 treatments (Supplementary Table S10), although preclinical studies will be required to determine their efficacy. In this direction, 3 of the 16 compounds are being evaluated under different clinical trials (indomethacin (*n* = 2), dexamethasone (*n* = 40), and spironolactone (*n* = 4)).

Rutin and indomethacin were amongst the notable compounds selected from 3CL main protease. In addition, they have been proven as good candidates in other studies. Rutin is a polyphenolic flavonoid that has shown a wide range of pharmacological applications due to its significant antioxidant properties [50]. Our results from GSEA analyses revealed that rutin might act in early stages of SARS-CoV-2 infection by activating the interferon-induced ISG15 pathway. ISG15 is an interferon-induced protein that has been implicated as a central player in the host antiviral response, and is the key element for the innate immune response against viral infection [51]. Furthermore, ISG15 modulates the immune system stimulating the IFN-gamma production by NK cells that lead to the promotion of early viral response [52]. Although the result of the possible interaction between rutin and 3CL protease has been reported by other studies using an in silico approach [53], our results provide a transcriptomic dimension to the possible effect of rutin during infection with SARS-CoV-2. Moreover, to our knowledge this is the first time the natural compound rutin has been related with the antiviral activity induced by the protein ISG15.

Dexamethasone, a corticosteroid used in a wide range of conditions for its anti-inflammatory and immunosuppressive effects, could be one of the most promising repurposed drugs chosen to treat COVID-19 disease, based on some results that prove a decrease in the incidence of death versus the usual care group among patients receiving invasive mechanical ventilation [54]. This compound was chosen because of its immunosuppressant properties to treat the cytokine storm induced by the immune response to coronavirus infection in late stages of the disease. Nonetheless, our results indicated that dexamethasone could also be a good candidate to target nsp15 endoribonuclease, although some repurposed works also suggested it as the target of the main protease [55]. These data could support the idea of administering corticosteroids, not just at the advanced infection stage, but also at the beginning. However, a recent study tested multiple pharmacological compounds derived from the steroids in vitro and demonstrated that dexamethasone has no antiviral activity against SARS-CoV-2 [56]. Nevertheless, we also found other corticosteroids that could interact with nsp15 protein, such as mifepristone, which suppressed viral growth conferring more than 95% of cell survival rate after viral infection and drug administration in vitro [56].

Lastly, the RNA-dependent RNA polymerase nsp12 of SARS-CoV-2 is a protein that performs essential functions in the coronavirus life cycle with no host cell homolog. This is an advantage for antiviral drug development, reducing the risk of affecting any protein present in human cells, as has been proven by many drug repurposing studies directed against nsp12 RdRP [57,58,59,60]. Vemurafenib, sorafenib, and raloxifene may be potential candidates against nsp12 RdRP. Vemurafenib can disturb the cellular Raf/MEK/ERK signaling cascade via binding in the ATP-binding site of BRAF(V600E) kinase and inhibiting its function [61], whereas sorafenib is another kinase inhibitor that targets VEGFR, PDGFR, and RAF kinases [62]. Interestingly, SARS-CoV-1 uses Raf/MEK/ERK signaling pathways to promote its replication via various mechanisms, indicating that this signaling cascade is a critical therapeutic target for host-directed SARS-CoV-2 antivirals [63,64,65].

## 4. Materials and Methods

### 4.1. Data Acquisition

DrugBank queries were carried out to retrieve the information regarding drugs with known protein targets [66]. In short, the DrugBank database version 5.1.5 (https://go.drugbank.com/releases/5-1-5, accessed on 21 March 2020) was downloaded in XML format, and the dbparser package and custom R scripts were employed to extract the relevant information [67]. We only selected drugs approved by the American Food and Drug Administration (FDA) and retrieved the names and UniProt identifiers of their protein targets. Then, UniProt IDs were mapped to their respective Protein Data Bank (PDB) structures using the Retrieve/ID mapping tool available at UniProt. All of the PDB structures targeted by FDA-approved drugs were downloaded in PDB format and stored for downstream analysis. Protein Data Bank queries were also performed to identify the three-dimensional structures of SARS-CoV-2 proteins.

### 4.2. A Topological Data Analysis Based Formalism to Compare, at Quantitative Level, the Homological Similarities of Pairwise Three-Dimensional Molecules Considered as Surfaces

In this paper, we used an adapted a TDA-based strategy which combines concepts and results from Algebraic Topology to compare three-dimensional protein structures [68,69,70]. More precisely, we considered the shape of the protein structure as a surface for which we only know a sample of points that are given by the coordinates of its $C_{\alpha }$. Using this information, we construct a set of simplicial complexes associated to that protein. This set is composed by three classes of geometrical objects: isolated points, non-intersecting segments connecting these points, and non-intersecting triangles composed using non-intersecting segments. To quantify the above geometrical information, we associate a non-negative continuous function to each of the three components of a simplicial complex. The first function, denoted by $f_{0},$ represents the structure of the position of the individual points, the second function $f_{1},$ corresponds to the non-intersecting segments and finally, the third function $f_{2}$ correspond to the triangles. These three functions are called the persistent Betti Functions (PBFs) and allow us to characterize the representation of a protein’s tertiary structure.

Therefore, we computed the persistent Betti functions using PDB structures from DrugBank. To compare the shape of both structures, one given by the PBF ${\left\{f_{i}\right\}}_{i=0}^{i=2}$ of each structure from DrugBank, against the PBF of *SARS-CoV*-2 proteins ${\left\{f_{i}^{SARS-Cov-2}\right\}}_{i=0}^{i=2}$ we construct the persistent similarity measure (*PSM*), which is defined as

(1) $$PSM_{i}=\frac{\int \min\left(f_{i}\left(x\right),f_{i}^{SARS-Cov-2}\left(x\right)\right)dx}{\int \max\left(f_{i}\left(x\right),f_{i}^{SARS-Cov-2}\left(x\right)\right)dx}\;\mathrm{for}\;i=0,1,2.$$

Then, we calculate the mean of the persistent similarity measures:

(2) $$\bar{PSM}=\frac{1}{3}\left(PSM_{0}+PSM_{1}+PSM_{2}\right)$$

for each protein comparison. A $\bar{PSM}\geq 0.9$ threshold value was established, considering those drugs whose target protein had a value of 0.9 or higher for their mean persistent similarity measure with a *SARS-CoV*-2 protein as drug repurposing candidates.

### 4.3. Data Preprocessing and Persistent Similarity Measures Computation

All protein structures in PDB format were loaded into the R environment using the bio3d package [71]. Then, the coarse-grain representation of each structure was generated by selecting only the three-dimensional atomic coordinates of the alpha-carbons of the amino acids [26]. Two main reasons compelled us to work with this reduced representation. First, the construction of simplicial complexes scales exponentially with the number of initial points present in the point cloud. Therefore, structures defined by a very large number of points are not computationally tractable even in state-of-the-art computers. Second, all-atom models present a high degree of detail that could mask the general structure of the protein. Barcodes were constructed using the R package of TDAstats [72]. TDAstats makes use internally of the Ripser C++ library [73], an optimized fast software package for simplicial complexes and barcodes construction.

### 4.4. Protein–Ligand Binding with AutoDock 4.2

Ligand preparation was carried out as follows: First, the FDA-approved drugs in SDF format were retrieved from DrugBank. A custom R script and Open Babel v.3.0.0 were used to transform SDF into the mol2 format [74,75,76,77]. Following, the MGLTools v.1.5.7 toolkit was employed to add the polar hydrogens and protonation at pH 7.4. Then, mol2 drug structures were converted into PDBQT format, and their stereochemical properties were computed using AutoDock 4.2 [78]. A virtual screening library was then constructed using the preprocessed drug structures. Drugs containing atoms different from those included in the following list (H, C, N, O, F, Mg, P, S, Cl, Ca, Mn, Fe, Zn, Br, I) were discarded from the subsequent analyses because AutoDock does not include the values of their atomic force fields and is, therefore, unable to perform molecular docking using them. Polar hydrogens were also added to the SARS-CoV-2 protein PDB structures which were also transformed to the PDBQT format. Docking was carried out using AutoDock 4.2 [78], a molecular docking software package developed by the Scripps Research Institute. A grid box spanning the whole protein structure was set to perform blind docking. AutoDock was configured following the manual recommendations [79]. We increased the parameter ga_runs from 10 to 150 to improve the accuracy of the results.

### 4.5. Differential Gene Expression Analyses of SARS-CoV-2 Infected Human Samples and Cell Lines and Uninfected Controls

We carried out searches for transcriptomic datasets of patients and human-derived cell lines including samples infected with SARS-CoV-2 and uninfected controls. At the time the searches were carried out, three datasets were identified. Dataset 1 (DS1) was found in the gene expression omnibus (GEO) under ID GSE150316 [80]. This includes formalin-fixed paraffin-embedded samples from multiple tissues (i.e., lung, jejunum, heart) derived from SARS-CoV-2-infected individuals and uninfected controls obtained in autopsies. We restricted our analysis to lung samples. Twenty-one samples (16 cases and five controls) were selected for downstream analysis.

Dataset 2 (DS2) gathers samples derived from bronchoalveolar lavage fluids (BALF) of SARS-CoV-2 infected patients (four samples derived from two patients with two technical replicates) and three healthy controls [81]. Samples derived from infected patients were stored at the National Genomics Data Center under accession number CRA002390, whereas control samples were downloaded from the NCBI SRA database and were available under the identifiers SRR10571724, SRR10571730, and SRR10571732. Sequence alignment using the human reference genome hGR38 and count extraction were carried out using the Rsubread package [82].

Finally, the third dataset (DS3) was available in GEO under accession ID GSE147507 [83]. It presented a complex design including both primary cell lines derived from the human lung epithelium and transformed lung alveolar which were either mock treated or infected with different viruses including the influenza A virus (IAV), the respiratory syncytial virus (RSV), and SARS-CoV-2, in addition to samples derived from infected ferrets and two technical replicates of a lung sample derived from a SARS-CoV-2-infected human patient. We restricted our analysis to the cell lines NHBE, A549, and Calu-3, which were either infected with SARS-CoV-2 or were mock treated. The infected human lung samples and the healthy lung biopsies were also included. Overall, 28 samples were analyzed in this dataset.

For each dataset, differential gene expression analysis between SARS-CoV-2 infected samples and uninfected controls was carried out using the DESeq2 package [84].

### 4.6. Identification of LINCS 1000 Signatures Negatively Correlated with the SARS-CoV-2 Differential Gene Expression Profiles

LINCS L1000 contains an extensive collection of gene expression profiles generated using thousands of perturbagens (i.e., small molecules, ligands, micro-environments, CRISPR gene over-expression, and knockdown perturbations) and different cell lines, doses, and exposure times [85]. In particular, LINCS L1000 Level 5 data includes differential gene expression signatures computed by comparing three technical replicates of the same perturbation to appropriate controls. Level 5 LINCS L1000 phases I (GSE92742) and II (GSE70138) datasets were downloaded from GEO. Signatures involving FDA-approved drugs were identified with the help of the information contained in file *repurposing_drugs_20180907.txt* and *repurposing_samples_20180907.txt* available at the LINCS L1000 repurposing hub [85] (see Supplementary Materials). Drugbank and LINCS 1000 data were merged based on Pubchem compound identifiers. Then, the subset of signatures corresponding to FDA approved medications with 435 known Pubchem identifiers were selected. Overall, we obtained 52,144 expression signatures generated using 1313 approved drugs. To identify drugs with the potential of reverting the differential expression profiles generated by SARS-CoV-2 infection, we computed Pearson’s correlations between each expression signature derived from LINCS L1000 and the differential expression profiles from DS1, DS2, and DS3, and picked those drugs exhibiting the most negative correlations.

### 4.7. Gene Set Enrichment Analysis (GSEA)

Dysregulated biological processes were identified for each transcriptomic dataset using the pre-ranked Gene Set Enrichment Analysis (GSEA) implementation of the fgsea package [86]. The C5 molecular signatures collection, which contains gene sets derived from the three branches of Gene Ontology (GO), was used as a source of functional information. GO terms including more than 500 or less than 15 genes were filtered out. GSEA analyses were also performed for those LINCS L1000 level 5 expression signatures negatively correlated with the differential gene expression profiles generated by the SARS-CoV-2 infection to determine their effect in specific pathways and biological processes. Reactome (version 73) was used as a source of pathway information and analyses were carried out using the clusterProfiler R-package (https://www.rdocumentation.org/packages/clusterProfiler/versions/3.0.4, accessed on 21 March 2020) [87]. Biological processes and pathways presenting false discovery rate (FDR) adjusted *p*-values were called to be significantly deregulated.

## 5. Conclusions

In conclusion, our strategy of quantitative homological similarities using TDA-based formalism would allow researchers and clinicians to select optimal candidates from drug repurposing to achieve the desired target, not only regarding the SARS-CoV-2 coronavirus, but also any new viruses that may appear in the future, by choosing the best targets among all virus proteins. In this specific case, targeting nsp15 endonuclease and nsp12 RNA polymerase, in addition to other promising drug targets of the 3CL main protease, could support the development of a cocktail of anti-coronavirus treatments that could also be potentially used for the discovery of broad-spectrum antivirals. In particular, we identified 16 potential repurposable drug candidates including cholic acid, rutin, indomethacin, sulindac, sulfisoxazole, dasatinib, dexamethasone, phenolphthalein, spironolactone, mifepristone, carbamazepine, vemurafenib, sorafenib, levonorgestrel, naloxone, and raloxifene. Furthermore, by choosing a precision multidrug treatment, we could rescue any specific drug failure or avoid any future drug resistance due to possible acquired mutations in any of the proteins as a consequence of continuous virus replication and spreading, because the virus will be attacked from different fronts. Nevertheless, our results based on multidrug combinations should be validated in both in vitro and in vivo experiments, not just to prove the effectiveness of the treatment, but also to select the best combination against SARS-CoV-2 infection and consequent disease symptoms.

## Figures and Tables

**Table 1:** Protein Data Bank (PDB) structures of SARS-CoV-2 proteins analyzed in the study. Entry ID (column 1) encodes the PDB identifyers of the analyzed protein structures, Structure Title (column 2) provides the protein structure description, Macromolecular Name (column 3) is the protein short name and Chain ID (column 4) are the studied chains.

Entry ID	Structure Title	Macromolecule Name	Chain ID
6LVN	2019-nCoV HR2 Domain	Spike protein S2	A, B, C, D
6YI3	The N-terminal RNA-binding domain of the SARS-CoV-2 nucleocapsid phosphoprotein	Nucleoprotein	A
6M3M	SARS-CoV-2 nucleocapsid protein N-terminal RNA binding domain	SARS-CoV-2 nucleocapsid protein	A, B, C, D
6VYO	RNA binding domain of nucleocapsid phosphoprotein from SARS coronavirus 2	Nucleoprotein	A, B, C, D
6WJI	C-terminal Dimerization Domain of Nucleocapsid Phosphoprotein from SARS-CoV-2	SARS-CoV-2 nucleocapsid protein	A, B, C, D, E, F
6LXT	Structure of post fusion core of 2019-nCoV S2 subunit	Spike protein S2	A, B, C, D, E, F
6VSB	Prefusion 2019-nCoV spike glycoprotein with a single receptor-binding domain up	SARS-CoV-2 spike glycoprotein	A, B, C
6VYB	SARS-CoV-2 spike ectodomain structure (open state)	Spike glycoprotein	A, B, C
6W41	Crystal structure of SARS-CoV-2 receptor binding domain in complex with human antibody CR3022	CR3022 Fab heavy chain	H
		CR3022 Fab light chain	L
		Spike protein S1	C
6YLA	Crystal structure of the SARS-CoV-2 receptor binding domain in complex with CR3022 Fab	Spike glycoprotein	A, E
		Heavy Chain	B, H
		Light chain	C, L
6M0J	Crystal structure of SARS-CoV-2 spike receptor-binding domain bound with ACE2	Angiotensin converting enzyme 2	A
		Spike receptor binding domain	E
6M17	2019-nCoV RBD/ACE2-B0AT1 complex	Sodium-dependent neutral amino acid transporter B(0)AT1	A, C
		Angiotensin converting enzyme 2	B, D
		SARS-coV-2 Receptor Binding Domain	E, F
6M2Q	SARS-CoV-2 3CL protease (3CL pro) apo structure (space group C21)	SARS-CoV-2 3CL protease	A
6W4B	Crystal structure of Nsp9 RNA binding protein of SARS CoV-2	Non-structural protein 9	A, B
6W9Q	Peptide-bound SARS-CoV-2 Nsp9 RNA replicase	3C-like proteinase peptide, Nonstructural protein 9 fusion	A
6VXS	Crystal Structure of ADP ribose phosphatase of NSP3 from SARS CoV-2	Non-structural protein 3	A, B
6W9C	Crystal structure of papain-like protease of SARS CoV-2	Papain-like proteinase	A, B, C
6WCF	Crystal Structure of ADP ribose phosphatase of NSP3 from SARS-CoV-2 in complex with MES	Non-structural protein 3	A
6WEN	Crystal Structure of ADP ribose phosphatase of NSP3 from SARS-CoV-2 in the apo form	Non-structural protein 3	A
6WIQ	Crystal structure of the co-factor complex of NSP7 and the C-terminal domain of NSP8 from SARS CoV-2	SARS-CoV-2 NSP7	A
		SARS-CoV-2 NSP8	B
6M71	SARS-Cov-2 RNA-dependent RNA polymerase in complex with cofactors	SARS-Cov-2 NSP 12	A
		SARS-Cov-2 NSP 8	C
		SARS-Cov-2 NSP 7	B, D
6W01	1.9 A Crystal Structure of NSP15 Endoribonuclease from SARS CoV-2 in the Complex with a Citrate	Uridylate-specific endoribonuclease	A, B
6VWW	Crystal Structure of NSP15 Endoribonuclease from SARS CoV-2	Uridylate-specific endoribonuclease	A, B

**Table 2:** Drug repurposing candidates based on the topological, trascriptomic, and docking criteria. PC: Pearson correlation. LE: Lowest energy conformation in the cluster. Candidates with a PC of <−0.1 may revert the transcriptomic effects of SARS-CoV-2 infection. The maximum number of the AutoDock cluster is 150. Drug ID (colum 2) encodes the DrugBank ID of the corresponding drug (column 1).

6M2Q (SARS-CoV-2 3CL Protease)						
Drug Name	Drug ID	PC DS1 (GSE150316)	PC DS2 (CRA002390)	PC DS3 (GSE147507)	AutoDock LE (kcal/mol)	AutoDock Cluster
CholicAcid	DB02659	−0.09	−0.11	−0.08	−15.06	74
Rutin	DB01698	−0.07	−0.18	−0.1	−14.52	149
Indomethacin	DB00328	−0.07	−0.12	−0.05	−13.31	146
Sulindac	DB00605	−0.07	−0.12	−0.07	−13.14	73
Sulfisoxazole	DB00263	−0.05	−0.13	−0.09	−11.59	77
Dasatinib	DB01254	−0.04	−0.15	−0.09	−10.94	43
**6W01 (NSP15 Endoribonuclease)**						
Dexamethasone	DB01234	−0.07	−0.15	−0.08	−11.42	49
Phenolphthalein	DB04824	−0.13	−0.1	−0.04	−11.15	101
Spironolactone	DB00421	−0.12	−0.1	−0.09	−10.99	110
Mifepristone	DB00834	−0.13	−0.14	−0.06	−10.04	28
Carbamazepine	DB00564	−0.08	−0.14	−0.07	−9.66	86
**6M71 (NSP12 RNA-dependent RNA polymerase)**						
Vemurafenib	DB08881	−0.09	−0.16	−0.08	−8.09	13
Sorafenib	DB00398	−0.11	−0.15	−0.05	−7.34	30
Levonorgestrel	DB00367	−0.08	−0.14	−0.08	−7.21	89
Naloxone	DB01183	−0.06	−0.12	−0.09	−7.07	69
Raloxifene	DB00481	−0.13	−0.17	−0.07	−7.05	6

## Data Availability

All data used in this work was obtained from the following public repositories: Drug Bank (https://go.drugbank.com/ (accessed on 21 March 2020)), Gene Expression Omnibus (https://www.ncbi.nlm.nih.gov/geo/ (accessed on 21 March 2020)), Protein Data Bank (https://www.rcsb.org/ (accessed on 21 March 2020)), and the Genome Sequence Archive (https://bigd.big.ac.cn/gsa/browse/CRA002390 (accessed on 21 March 2020)).

## Supplementary Materials

The following are available online at https://www.mdpi.com/article/10.3390/pharmaceutics13040488/s1, File S1: Differential gene expression and GSEA analyses results for the three transcriptomic datasets, File S2: INCS L1000 analyses results for the three transcriptomic datasets, File S3: Supplementary tables. File S4: interacting residues between the viral proteins and the drugs identified as potential repurposing candidates. File S5: repurposing_drugs_20180907. File S6: repurposing_samples_20180907. Table S1: Proteins targeted by Drugbank FDA-approved medications showing average persistent similarity measures higher than 0.9 with 6M2Q, Table S2: Transcriptomic and molecular docking analyses results for drugs with the potential of targeting the SARS-CoV-2 3CL protease in apo conformation (6M2Q), Table S3: Proteins targeted by Drugbank FDA-approved medications showing average persistent similarity measures higher than 0.9 with 6M71, Table S4: Transcriptomic and molecular docking analyses results for drugs with the potential of target-ing the SARS-CoV-2 RNA dependent RNA polymerase (6M71), Table S5: Proteins targeted by Drugbank FDA-approved medications showing average persistent similarity measures higher than 0.9 with 6W01, Table S6: Transcriptomic and molecular docking analyses results for drugs with the potential of targeting the SARS-CoV-2 NSP15 Endoribonuclease (6W01), Table S7: GSEA results for top drugs targeting the 3CL protease (6M2Q), Table S8: GSEA results for top drugs tar-geting the RNA-dependent RNA polymerase (NSP12)(6M71), Table S9: GSEA results for top drugs targeting the SP15 Endoribonuclease (6W01), Table S10: Previous research analyzing the effects of our candidate drugs in SARS-CoV-2 infection.
