# Supplementary material for: A COVID-19 Drug Repurposing Strategy through Quantitative Homological Similarities Using a Topological Data Analysis-Based Framework
Source: Pharmaceutics. 2021 Apr 2;13(4):488. doi: 10.3390/pharmaceutics13040488 (PMC8066156; doi:10.3390/pharmaceutics13040488)
Supplement: Supplementary file 1 [file pharmaceutics-13-00488-s001.zip › SupplementaryFile3.pdf]

Supplementary Materials 3 for *A COVID-19 Drug Repurposing Strategy through Quantitative Homological Similarities Using a Topological Data Analysis-Based Framework*

1. Supplementary tables

PDB ID	Residue count	EC number	SCOPE fold	Classification	CATH description	Protein Family Annotation	Mean persistent similary
104L	332	NA	Lysozyme-Like	Hydrolase(O-Glycosyl)	NA	Phage Lysozyme	0.906
1A5Y	330	3.1.3.48	(Phosphotyrosine Protein) Phosphatases Ii	Hydrolase	Protein Tyrosine Phosphatase Superfamily	Protein-Tyrosine Phosphatase	0.909
1AAX	321	3.1.3.48	(Phosphotyrosine Protein) Phosphatases Ii	Hydrolase	Protein Tyrosine Phosphatase Superfamily	Protein-Tyrosine Phosphatase	0.907
1ABI	315	3.4.21.5	NA	Hydrolase/Hydrolase Inhibitor	Trypsin-Like Serine Proteases	Trypsin	0.908
1ADS	315	1.1.1.21	Tim Beta/Alpha-Barrel	Oxidoreductase	Nadp-Dependent Oxidoreductase	Aldo/Keto Reductase Family	0.902
1AE8	305	3.4.21.5	NA	Hydrolase/Hydrolase Inhibitor	Trypsin-Like Serine Proteases	Trypsin	0.905
1AFE	305	3.4.21.5	NA	Hydrolase/Hydrolase Inhibitor	Trypsin-Like Serine Proteases	Trypsin	0.912
1AI8	307	3.4.21.5	NA	Blood Coagulation/Hydrolase Inhibitor	Trypsin-Like Serine Proteases	Trypsin	0.902
1AJ0	282	2.5.1.15	Tim Beta/Alpha-Barrel	Synthase	Dihydropteroate (Dhp) Synthetase	Pterin Binding Enzyme	0.900
1AY6	308	3.4.21.5	NA	Hydrolase/Hydrolase Inhibitor	Trypsin-Like Serine Proteases	Trypsin	0.903
1AZ2	315	1.1.1.21	Tim Beta/Alpha-Barrel	Oxidoreductase	Nadp-Dependent Oxidoreductase	Aldo/Keto Reductase Family	0.915
1C1W	306	3.4.21.5	NA	Hydrolase/Hydrolase Inhibitor	Trypsin-Like Serine Proteases	Trypsin	0.900
1C4Y	308	3.4.21.5	Trypsin-Like Serine Proteases	Hydrolase/Hydrolase Inhibitor	NA	Thrombin Light Chain	0.900
1C5L	305	3.4.21.5	NA	Blood Clotting/Hydrolase Inhibitor	Trypsin-Like Serine Proteases	Trypsin	0.903
1C5N	305	3.4.21.5	NA	Hydrolase/Hydrolase Inhibitor	Trypsin-Like Serine Proteases	Trypsin	0.907
1D3T	307	3.4.21.5	Trypsin-Like Serine Proteases	Hydrolase/Hydrolase Inhibitor	NA	Thrombin Light Chain	0.902
1DM4	306	NA	Trypsin-Like Serine Proteases	Hydrolase	NA	Thrombin Light Chain	0.905
1DWC	306	3.4.21.5	NA	Hydrolase/Hyrolase Inhibitor	Trypsin-Like Serine Proteases	Trypsin	0.901
1E25	282	3.5.2.6	Beta-Lactamase/Transpeptidase-Like	Hydrolase	Dd-Peptidase/Beta-Lactamase Superfamily	Beta-Lactamase Enzyme Family	0.912
1EB1	297	NA	NA	Hydrolase/Hydrolase Inhibitor	NA	NA	0.904
1EEO	332	3.1.3.48	(Phosphotyrosine Protein) Phosphatases Ii	Hydrolase/Hydrolase Substrate	Protein Tyrosine Phosphatase Superfamily	Protein-Tyrosine Phosphatase	0.913
1EL3	316	1.1.1.21	Tim Beta/Alpha-Barrel	Oxidoreductase	Nadp-Dependent Oxidoreductase	Aldo/Keto Reductase Family	0.924
1ENY	268	NA	Nad(P)-Binding Rossmann-Fold Domains	Oxidoreductase	Nad(P)-Binding Rossmann-Like Domain	Enoyl-(Acyl Carrier Protein) Reductase	0.906
1G30	306	3.4.21.5	Trypsin-Like Serine Proteases	Hydrolase/Hydrolase Inhibitor	NA	Thrombin Light Chain	0.909
1G32	306	3.4.21.5	Trypsin-Like Serine Proteases	Hydrolase/Hydrolase Inhibitor	NA	Thrombin Light Chain	0.906
1GHV	304	3.4.21.5	NA	Hydrolase/Hydrolase Inhibitor	Trypsin-Like Serine Proteases	Trypsin	0.910
1GJ5	305	3.4.21.5	NA	Blood Clotting,Hydrolase/Inhibitor	Trypsin-Like Serine Proteases	Trypsin	0.901
1H8I	290	3.4.21.5	NA	Hydrolase/Hydrolase Inhibitor	Trypsin-Like Serine Proteases	Trypsin	0.903
1HAH	305	3.4.21.5	NA	Complex(Serine Proteinase/Inhibitor)	Trypsin-Like Serine Proteases	Trypsin	0.912
1HAI	295	3.4.21.5	NA	Hydrolase/Hydrolase Inhibitor	Trypsin-Like Serine Proteases	Trypsin	0.904
1HDT	304	3.4.21.5	NA	Hydrolase/Hydrolase Inhibitor	Trypsin-Like Serine Proteases	Trypsin	0.910
1IHS	316	3.4.21.5	NA	Hydrolase/Hydrolase Inhibitor	Trypsin-Like Serine Proteases	Trypsin	0.912
1JWT	305	3.4.21.5	Trypsin-Like Serine Proteases	Hydrolase	Trypsin-Like Serine Proteases	Thrombin Light Chain	0.904
1KAV	298	3.1.3.48	(Phosphotyrosine Protein) Phosphatases Ii	Hydrolase	Protein Tyrosine Phosphatase Superfamily	Protein-Tyrosine Phosphatase	0.903
1KW0	325	1.14.16.1	Aromatic Aminoacid Monoxygenases, Catalytic And Oligomerization Domains	Oxidoreductase	Phenylalanine Hydroxylase	Biopterin-Dependent Aromatic Amino Acid Hydroxylase	0.907
1MAR	315	1.1.1.21	Tim Beta/Alpha-Barrel	Oxidoreductase(Nadp)	NA	Aldo/Keto Reductase Family	0.921
1MMK	325	1.14.16.1	Aromatic Aminoacid Monoxygenases, Catalytic And Oligomerization Domains	Oxidoreductase	Phenylalanine Hydroxylase	Biopterin-Dependent Aromatic Amino Acid Hydroxylase	0.921
1MMT	325	1.14.16.1	Aromatic Aminoacid Monoxygenases, Catalytic And Oligomerization Domains	Oxidoreductase	Phenylalanine Hydroxylase	Biopterin-Dependent Aromatic Amino Acid Hydroxylase	0.906
1MRQ	323	1.1.1.149	Tim Beta/Alpha-Barrel	Oxidoreductase	Nadp-Dependent Oxidoreductase	Aldo/Keto Reductase Family	0.904
1MUE	306	3.4.21.5	Trypsin-Like Serine Proteases	Hydrolase Inhibitor	NA	Thrombin Light Chain	0.905
1NO9	306	3.4.21.5	NA	Hydrolase/Hydrolase Inhibitor	Trypsin-Like Serine Proteases	Trypsin	0.901
1NRN	318	3.4.21.5	NA	Serine Proteinase/Receptor	Trypsin-Like Serine Proteases	Trypsin	0.907
1NRS	311	3.4.21.5	NA	Hydrolase/Hydrolase Inhibitor	Trypsin-Like Serine Proteases	Trypsin	0.905
1NW9	375	NA	Inhibitor Of Apoptosis (Iap) Repeat	Apoptosis	Inhibitor Of Apoptosis Protein (2mihbc-Iap-1); Chain A	Inhibitor Of Apoptosis Domain	0.901
1OES	321	3.1.3.48	(Phosphotyrosine Protein) Phosphatases Ii	Hydrolase	Protein Tyrosine Phosphatase Superfamily	Protein-Tyrosine Phosphatase	0.902
1OEV	321	3.1.3.48	(Phosphotyrosine Protein) Phosphatases Ii	Hydrolase	Protein Tyrosine Phosphatase Superfamily	Protein-Tyrosine Phosphatase	0.901
1ONY	321	3.1.3.48	(Phosphotyrosine Protein) Phosphatases Ii	Hydrolase	Protein Tyrosine Phosphatase Superfamily	Protein-Tyrosine Phosphatase	0.915
1PF7	289	2.4.2.1	Phosphorylase/Hydrolase-Like	Transferase	NA	Phosphorylase Superfamily	0.912
1PME	380	2.7.1.-	Protein Kinase-Like (Pk-Like)	Transferase	Phosphorylase Kinase; Domain 1	Protein Kinase Domain	0.916
1PMU	364	2.7.1.-	Protein Kinase-Like (Pk-Like)	Transferase	Phosphorylase Kinase; Domain 1	Protein Kinase Domain	0.911
1PTT	326	3.1.3.48	(Phosphotyrosine Protein) Phosphatases Ii	Complex (Hydrolase/Peptide)	Protein Tyrosine Phosphatase Superfamily	Protein-Tyrosine Phosphatase	0.911
1PTV	321	3.1.3.48	(Phosphotyrosine Protein) Phosphatases Ii	Complex (Hydrolase/Peptide)	Protein Tyrosine Phosphatase Superfamily	Protein-Tyrosine Phosphatase	0.905
1PTY	321	3.1.3.48	(Phosphotyrosine Protein) Phosphatases Ii	Hydrolase	Protein Tyrosine Phosphatase Superfamily	Protein-Tyrosine Phosphatase	0.902
1PWL	316	1.1.1.21	Tim Beta/Alpha-Barrel	Oxidoreductase	Nadp-Dependent Oxidoreductase	Aldo/Keto Reductase Family	0.910
1PWM	316	1.1.1.21	Tim Beta/Alpha-Barrel	Oxidoreductase	Nadp-Dependent Oxidoreductase	Aldo/Keto Reductase Family	0.920
1PWY	288	2.4.2.1	Phosphorylase/Hydrolase-Like	Transferase	NA	Phosphorylase Superfamily	0.902
1QBV	306	NA	NA	Hydrolase/Hydrolase Inhibitor	NA	NA	0.900
1QHR	305	3.4.21.5	Trypsin-Like Serine Proteases	Blood Clotting/Hydrolase Inhibitor	NA	Thrombin Light Chain	0.900
1QUR	303	NA	NA	Hydrolase/Hydrolase Inhibitor	Trypsin-Like Serine Proteases	Trypsin	0.906
1RCT	288	2.4.2.1	Phosphorylase/Hydrolase-Like	Transferase	NA	Phosphorylase Superfamily	0.903
1RW8	301	2.7.1.37	NA	Transferase	Transferase(Phosphotransferase) Domain 1	Protein Kinase Domain	0.913
1S2A	331	1.1.1.213	Tim Beta/Alpha-Barrel	Oxidoreductase	Nadp-Dependent Oxidoreductase	Aldo/Keto Reductase Family	0.905
1S2C	331	1.1.1.213	Tim Beta/Alpha-Barrel	Oxidoreductase	Nadp-Dependent Oxidoreductase	Aldo/Keto Reductase Family	0.904
1SVC	384	NA	NA	Transcription/Dna	NA	NA	0.906
1T40	316	1.1.1.21	Tim Beta/Alpha-Barrel	Oxidoreductase	Nadp-Dependent Oxidoreductase	Aldo/Keto Reductase Family	0.916
1T48	298	3.1.3.48	(Phosphotyrosine Protein) Phosphatases Ii	Hydrolase	Protein Tyrosine Phosphatase Superfamily	Protein-Tyrosine Phosphatase	0.906
1THR	308	3.4.21.5	NA	Hydrolase(Serine Proteinase)	Trypsin-Like Serine Proteases	Trypsin	0.903
1TMB	313	3.4.21.5	NA	Hydrolase/Hydrolase Inhibitor	Trypsin-Like Serine Proteases	Trypsin	0.905
1TMT	316	3.4.21.5	NA	Hydrolase/Hydrolase Inhibitor	Trypsin-Like Serine Proteases	Trypsin	0.909
1UMA	305	3.4.21.5	NA	Hydrolase/Hydrolase Inhibitor	Trypsin-Like Serine Proteases	Trypsin	0.914
1V41	288	2.4.2.1	Phosphorylase/Hydrolase-Like	Transferase	NA	Phosphorylase Superfamily	0.921
1VJY	303	2.7.1.37	Protein Kinase-Like (Pk-Like)	Transferase	Phosphorylase Kinase; Domain 1	Protein Kinase Domain	0.931
1WAY	305	3.4.21.5	NA	Hydrolase/Hydrolase Inhibitor	NA	Thrombin Light Chain	0.903
1WBG	305	3.4.21.5	NA	Hydrolase/Hydrolase Inhibitor	NA	Thrombin Light Chain	0.905
1X96	316	1.1.1.21	Tim Beta/Alpha-Barrel	Oxidoreductase	Nadp-Dependent Oxidoreductase	Aldo/Keto Reductase Family	0.921
1X97	316	1.1.1.21	Tim Beta/Alpha-Barrel	Oxidoreductase	Nadp-Dependent Oxidoreductase	Aldo/Keto Reductase Family	0.916
1X98	316	1.1.1.21	Tim Beta/Alpha-Barrel	Oxidoreductase	Nadp-Dependent Oxidoreductase	Aldo/Keto Reductase Family	0.912
1XAS	299	3.2.1.8	Tim Beta/Alpha-Barrel	Xylanase A	NA	Glycosyl Hydrolase Family 10	0.921
1XM1	305	3.4.21.5	Trypsin-Like Serine Proteases	Hydrolase/Hydrolase Inhibitor	Trypsin-Like Serine Proteases	Trypsin	0.900
1YRY	289	2.4.2.1	Phosphorylase/Hydrolase-Like	Transferase	NA	Phosphorylase Superfamily	0.902
1Z3N	319	1.1.1.21	Tim Beta/Alpha-Barrel	Oxidoreductase	Nadp-Dependent Oxidoreductase	Aldo/Keto Reductase Family	0.930
1Z89	316	1.1.1.21	Tim Beta/Alpha-Barrel	Oxidoreductase	Nadp-Dependent Oxidoreductase	Aldo/Keto Reductase Family	0.921
1Z8A	316	1.1.1.21	Tim Beta/Alpha-Barrel	Oxidoreductase	Nadp-Dependent Oxidoreductase	Aldo/Keto Reductase Family	0.905
1ZUA	317	1.1.1.-	NA	Oxidoreductase	Nadp-Dependent Oxidoreductase	Aldo/Keto Reductase Family	0.913

PDB ID	Residue count	EC number	SCOPE fold	Classification	CATH description	Protein Family Annotation	Mean persistent similary
2ACQ	315	1.1.1.21	Tim Beta/Alpha-Barrel	Oxidoreductase	Nadp-Dependent Oxidoreductase	Aldo/Keto Reductase Family	0.905
2ACR	315	1.1.1.21	Tim Beta/Alpha-Barrel	Oxidoreductase	Nadp-Dependent Oxidoreductase	Aldo/Keto Reductase Family	0.902
2ACU	315	1.1.1.21	Tim Beta/Alpha-Barrel	Oxidoreductase	Nadp-Dependent Oxidoreductase	Aldo/Keto Reductase Family	0.921
2AGT	319	1.1.1.21	NA	Oxidoreductase	Nadp-Dependent Oxidoreductase	Aldo/Keto Reductase Family	0.915
2BGD	321	3.1.3.48	(Phosphotyrosine Protein) Phosphatases Ii	Hydrolase	Protein Tyrosine Phosphatase Superfamily	Protein-Tyrosine Phosphatase	0.900
2BGE	321	3.1.3.48	(Phosphotyrosine Protein) Phosphatases Ii	Hydrolase	Protein Tyrosine Phosphatase Superfamily	Protein-Tyrosine Phosphatase	0.912
2C93	307	3.4.21.5	NA	Hydrolase/Hydrolase Inhibitor	NA	Thrombin Light Chain	0.903
2CMA	327	3.1.3.48	(Phosphotyrosine Protein) Phosphatases Ii	Hydrolase	Protein Tyrosine Phosphatase Superfamily	Protein-Tyrosine Phosphatase	0.905
2DUZ	316	1.1.1.21	Tim Beta/Alpha-Barrel	Oxidoreductase	Nadp-Dependent Oxidoreductase	Aldo/Keto Reductase Family	0.902
2DV0	316	1.1.1.21	Tim Beta/Alpha-Barrel	Oxidoreductase	Nadp-Dependent Oxidoreductase	Aldo/Keto Reductase Family	0.910
2F2K	316	1.1.1.21	Tim Beta/Alpha-Barrel	Oxidoreductase	Nadp-Dependent Oxidoreductase	Aldo/Keto Reductase Family	0.911
2F6F	302	3.1.3.48	NA	Hydrolase	Protein Tyrosine Phosphatase Superfamily	Protein-Tyrosine Phosphatase	0.902
2FGB	323	1.1.1.62	Tim Beta/Alpha-Barrel	Oxidoreductase	Nadp-Dependent Oxidoreductase	Aldo/Keto Reductase Family	0.906
2FZ8	316	1.1.1.21	Tim Beta/Alpha-Barrel	Oxidoreductase	Nadp-Dependent Oxidoreductase	Aldo/Keto Reductase Family	0.906
2FZ9	316	1.1.1.21	Tim Beta/Alpha-Barrel	Oxidoreductase	Nadp-Dependent Oxidoreductase	Aldo/Keto Reductase Family	0.915
2FZB	316	1.1.1.21	Tim Beta/Alpha-Barrel	Oxidoreductase	Nadp-Dependent Oxidoreductase	Aldo/Keto Reductase Family	0.920
2G3J	313	3.2.1.8	NA	Hydrolase	Glycosidases	Glycosyl Hydrolase Family 10	0.915
2GGT	328	NA	Thioredoxin Fold	Chaperone	Glutaredoxin	Sco1/Senc	0.927
2H4G	299	3.1.3.48	(Phosphotyrosine Protein) Phosphatases Ii	Hydrolase	Protein Tyrosine Phosphatase Superfamily	Protein-Tyrosine Phosphatase	0.902
2HV5	316	1.1.1.21	Tim Beta/Alpha-Barrel	Oxidoreductase	Nadp-Dependent Oxidoreductase	Aldo/Keto Reductase Family	0.913
2HVN	316	1.1.1.21	Tim Beta/Alpha-Barrel	Oxidoreductase	Nadp-Dependent Oxidoreductase	Aldo/Keto Reductase Family	0.918
2HVO	316	1.1.1.21	Tim Beta/Alpha-Barrel	Oxidoreductase	Nadp-Dependent Oxidoreductase	Aldo/Keto Reductase Family	0.912
2IKG	316	1.1.1.21	Tim Beta/Alpha-Barrel	Oxidoreductase	Nadp-Dependent Oxidoreductase	Aldo/Keto Reductase Family	0.916
2IKH	316	1.1.1.21	Tim Beta/Alpha-Barrel	Oxidoreductase	Nadp-Dependent Oxidoreductase	Aldo/Keto Reductase Family	0.914
2IKJ	316	1.1.1.21	Tim Beta/Alpha-Barrel	Oxidoreductase	Nadp-Dependent Oxidoreductase	Aldo/Keto Reductase Family	0.905
2INE	315	1.1.1.21	Tim Beta/Alpha-Barrel	Oxidoreductase	Nadp-Dependent Oxidoreductase	Aldo/Keto Reductase Family	0.909
2INZ	315	1.1.1.21	Tim Beta/Alpha-Barrel	Oxidoreductase	Nadp-Dependent Oxidoreductase	Aldo/Keto Reductase Family	0.903
2IQ0	315	1.1.1.21	Tim Beta/Alpha-Barrel	Oxidoreductase	Nadp-Dependent Oxidoreductase	Aldo/Keto Reductase Family	0.909
2IQD	315	1.1.1.21	Tim Beta/Alpha-Barrel	Oxidoreductase	Nadp-Dependent Oxidoreductase	Aldo/Keto Reductase Family	0.909
2ISF	315	1.1.1.21	Tim Beta/Alpha-Barrel	Oxidoreductase	Nadp-Dependent Oxidoreductase	Aldo/Keto Reductase Family	0.905
2J8T	316	1.1.1.21	Tim Beta/Alpha-Barrel	Oxidoreductase	Nadp-Dependent Oxidoreductase	Aldo/Keto Reductase Family	0.903
2NVC	316	1.1.1.21	Tim Beta/Alpha-Barrel	Oxidoreductase	Nadp-Dependent Oxidoreductase	Aldo/Keto Reductase Family	0.913
2PD5	316	1.1.1.21	NA	Oxidoreductase	Nadp-Dependent Oxidoreductase	Aldo/Keto Reductase Family	0.928
2PD9	316	1.1.1.21	NA	Oxidoreductase	Nadp-Dependent Oxidoreductase	Aldo/Keto Reductase Family	0.907
2PDB	316	1.1.1.21	NA	Oxidoreductase	Nadp-Dependent Oxidoreductase	Aldo/Keto Reductase Family	0.918
2PDC	316	1.1.1.21	NA	Oxidoreductase	Nadp-Dependent Oxidoreductase	Aldo/Keto Reductase Family	0.923
2PDF	316	1.1.1.21	NA	Oxidoreductase	Nadp-Dependent Oxidoreductase	Aldo/Keto Reductase Family	0.924
2PDG	316	1.1.1.21	Tim Beta/Alpha-Barrel	Oxidoreductase	Nadp-Dependent Oxidoreductase	Aldo/Keto Reductase Family	0.909
2PDH	316	1.1.1.21	NA	Oxidoreductase	Nadp-Dependent Oxidoreductase	Aldo/Keto Reductase Family	0.907
2PDI	316	1.1.1.21	NA	Oxidoreductase	Nadp-Dependent Oxidoreductase	Aldo/Keto Reductase Family	0.912
2PDJ	316	1.1.1.21	NA	Oxidoreductase	Nadp-Dependent Oxidoreductase	Aldo/Keto Reductase Family	0.903
2PDK	316	1.1.1.21	NA	Oxidoreductase	Nadp-Dependent Oxidoreductase	Aldo/Keto Reductase Family	0.912
2PDL	316	1.1.1.21	NA	Oxidoreductase	Nadp-Dependent Oxidoreductase	Aldo/Keto Reductase Family	0.917
2PDN	316	1.1.1.21	NA	Oxidoreductase	Nadp-Dependent Oxidoreductase	Aldo/Keto Reductase Family	0.927
2PDP	316	1.1.1.21	NA	Oxidoreductase	Nadp-Dependent Oxidoreductase	Aldo/Keto Reductase Family	0.914
2PDQ	316	1.1.1.21	NA	Oxidoreductase	Nadp-Dependent Oxidoreductase	Aldo/Keto Reductase Family	0.904
2PDW	316	1.1.1.21	NA	Oxidoreductase	Nadp-Dependent Oxidoreductase	Aldo/Keto Reductase Family	0.904
2PEV	316	1.1.1.21	Tim Beta/Alpha-Barrel	Oxidoreductase	Nadp-Dependent Oxidoreductase	Aldo/Keto Reductase Family	0.903
2PGQ	304	3.4.21.5	NA	Hydrolase/Hydrolase Inhibitor	Epsilon-Thrombin, Subunit L	Thrombin Light Chain	0.902
2PZN	316	1.1.1.21	Tim Beta/Alpha-Barrel	Oxidoreductase	Nadp-Dependent Oxidoreductase	Aldo/Keto Reductase Family	0.925
2Q0N	312	2.7.11.1	NA	Transferase	Phosphorylase Kinase; Domain 1	Protein Kinase Domain	0.902
2QBR	299	3.1.3.48	NA	Hydrolase	Protein Tyrosine Phosphatase Superfamily	Protein-Tyrosine Phosphatase	0.900
2R24	316	1.1.1.21	NA	Oxidoreductase	Nadp-Dependent Oxidoreductase	Aldo/Keto Reductase Family	0.927
2RLC	328	3.5.1.24	NA	Hydrolase	Penicillin V Acylase; Chain A	Linear Amide C-N Hydrolases, Choloylglycine Hydrolase Family	0.910
2WOT	306	2.7.11.30	NA	Transferase	Transferase(Phosphotransferase) Domain 1	Protein Kinase Domain	0.901
2WOU	306	2.7.11.30	NA	Transferase	Phosphorylase Kinase; Domain 1	Protein Kinase Domain	0.917
3BCJ	316	1.1.1.21	Tim Beta/Alpha-Barrel	Oxidoreductase	Nadp-Dependent Oxidoreductase	Aldo/Keto Reductase Family	0.903
3C3U	323	1.1.1.149	Tim Beta/Alpha-Barrel	Oxidoreductase	Nadp-Dependent Oxidoreductase	Aldo/Keto Reductase Family	0.906
3D1V	289	2.4.2.1	NA	Transferase	NA	Phosphorylase Superfamily	0.903
3D42	316	3.1.3.48	NA	Hydrolase	Protein Tyrosine Phosphatase Superfamily	Protein-Tyrosine Phosphatase	0.930
3D9C	321	3.1.3.48	NA	Hydrolase	Protein Tyrosine Phosphatase Superfamily	Protein-Tyrosine Phosphatase	0.914
3DN5	316	1.1.1.21	NA	Oxidoreductase	Nadp-Dependent Oxidoreductase	Aldo/Keto Reductase Family	0.921
3FMN	372	2.7.11.24	NA	Signaling Protein, Transferase	Phosphorylase Kinase; Domain 1	Protein Kinase Domain	0.906
3G5E	319	1.1.1.21	NA	Oxidoreductase	Nadp-Dependent Oxidoreductase	Aldo/Keto Reductase Family	0.919
3GHR	316	1.1.1.21	NA	Oxidoreductase	Nadp-Dependent Oxidoreductase	Aldo/Keto Reductase Family	0.901
3GHS	316	1.1.1.21	NA	Oxidoreductase	Nadp-Dependent Oxidoreductase	Aldo/Keto Reductase Family	0.904
3GHT	316	1.1.1.21	NA	Oxidoreductase	Nadp-Dependent Oxidoreductase	Aldo/Keto Reductase Family	0.912
3GHU	316	1.1.1.21	NA	Oxidoreductase	Nadp-Dependent Oxidoreductase	Aldo/Keto Reductase Family	0.915
3GUG	323	1.1.1.-	NA	Oxidoreductase	Nadp-Dependent Oxidoreductase	Aldo/Keto Reductase Family	0.903
3HMM	303	2.7.11.30	NA	Transferase	Transferase(Phosphotransferase) Domain 1	Protein Kinase Domain	0.912
3HRB	359	2.7.11.24	NA	Transferase	Phosphorylase Kinase; Domain 1	Protein Kinase Domain	0.915
3INY	289	2.4.2.1	NA	Transferase	NA	Phosphorylase Superfamily	0.924
3LEN	316	1.1.1.21	NA	Oxidoreductase/Oxidoreductase Inhibitor	Nadp-Dependent Oxidoreductase	Aldo/Keto Reductase Family	0.916
3M0I	316	1.1.1.21	NA	Oxidoreductase/Oxidoreductase Inhibitor	Nadp-Dependent Oxidoreductase	Aldo/Keto Reductase Family	0.910
3M4H	316	1.1.1.21	NA	Oxidoreductase/Oxidoreductase Inhibitor	Nadp-Dependent Oxidoreductase	Aldo/Keto Reductase Family	0.907
3M64	316	1.1.1.21	NA	Oxidoreductase/Oxidoreductase Inhibitor	Nadp-Dependent Oxidoreductase	Aldo/Keto Reductase Family	0.909
3MB9	316	1.1.1.21	NA	Oxidoreductase/Oxidoreductase Inhibitor	Nadp-Dependent Oxidoreductase	Aldo/Keto Reductase Family	0.919
3MC5	316	1.1.1.21	NA	Oxidoreductase/Oxidoreductase Inhibitor	Nadp-Dependent Oxidoreductase	Aldo/Keto Reductase Family	0.915
3MFR	351	2.7.11.1	NA	Transferase	Phosphorylase Kinase; Domain 1	Protein Kinase Domain	0.911
3MFS	351	2.7.11.1	NA	Transferase	Phosphorylase Kinase; Domain 1	Protein Kinase Domain	0.912
3MFT	351	2.7.11.1	NA	Transferase	Transferase(Phosphotransferase) Domain 1	Protein Kinase Domain	0.903
3MFU	351	2.7.11.1	NA	Transferase	Phosphorylase Kinase; Domain 1	Protein Kinase Domain	0.917
3P2V	316	1.1.1.21	NA	Oxidoreductase/Oxidoreductase Inhibitor	Nadp-Dependent Oxidoreductase	Aldo/Keto Reductase Family	0.921
3QKP	321	3.1.3.48	NA	Hydrolase	Protein Tyrosine Phosphatase Superfamily	Protein-Tyrosine Phosphatase	0.902

PDB ID	Residue count	EC number	SCOPE fold	Classification	CATH description	Protein Family Annotation	Mean persistent similary
3R43	331	1.-.-.-	NA	Oxidoreductase/Oxidoreductase Inhibitor	Nadp-Dependent Oxidoreductase	Aldo/Keto Reductase Family	0.907
3R58	331	1.-.-.-	NA	Oxidoreductase/Oxidoreductase Inhibitor	Nadp-Dependent Oxidoreductase	Aldo/Keto Reductase Family	0.911
3R6I	331	1.-.-.-	NA	Oxidoreductase/Oxidoreductase Inhibitor	Nadp-Dependent Oxidoreductase	Aldo/Keto Reductase Family	0.903
3R7M	331	1.1.1.213	NA	Oxidoreductase/Oxidoreductase Inhibitor	Nadp-Dependent Oxidoreductase	Aldo/Keto Reductase Family	0.926
3R8G	331	1.-.-.-	NA	Oxidoreductase/Oxidoreductase Inhibitor	Nadp-Dependent Oxidoreductase	Aldo/Keto Reductase Family	0.931
3RX2	336	1.1.1.21	NA	Oxidoreductase	Nadp-Dependent Oxidoreductase	Aldo/Keto Reductase Family	0.907
3RX3	336	1.1.1.21	NA	Oxidoreductase	Nadp-Dependent Oxidoreductase	Aldo/Keto Reductase Family	0.914
3RX4	336	1.1.1.21	NA	Oxidoreductase	Nadp-Dependent Oxidoreductase	Aldo/Keto Reductase Family	0.924
3S3G	336	1.1.1.21	NA	Oxidoreductase	Nadp-Dependent Oxidoreductase	Aldo/Keto Reductase Family	0.914
3T42	319	1.1.1.21	NA	Oxidoreductase/Oxidoreductase Inhibitor	Nadp-Dependent Oxidoreductase	Aldo/Keto Reductase Family	0.905
3U2C	316	1.1.1.21	NA	Oxidoreductase/Oxidoreductase Inhibitor	Nadp-Dependent Oxidoreductase	Aldo/Keto Reductase Family	0.910
3UFY	331	1.1.1.213	NA	Oxidoreductase	Nadp-Dependent Oxidoreductase	Aldo/Keto Reductase Family	0.924
3UG8	331	1.1.1.213	NA	Oxidoreductase	Nadp-Dependent Oxidoreductase	Aldo/Keto Reductase Family	0.913
3UGR	331	1.1.1.213	NA	Oxidoreductase	Nadp-Dependent Oxidoreductase	Aldo/Keto Reductase Family	0.922
3UWE	331	1.1.1.213	NA	Oxidoreductase	Nadp-Dependent Oxidoreductase	Aldo/Keto Reductase Family	0.901
3V35	336	1.1.1.21	NA	Oxidoreductase	Nadp-Dependent Oxidoreductase	Aldo/Keto Reductase Family	0.917
3V36	336	1.1.1.21	NA	Oxidoreductase	Nadp-Dependent Oxidoreductase	Aldo/Keto Reductase Family	0.902
3VXF	315	3.4.21.5	NA	Hydrolase/Hydrolase Inhibitor	NA	Trypsin	0.907
4ANP	324	1.14.16.1	NA	Oxidoreductase	Phenylalanine Hydroxylase	Biopterin-Dependent Aromatic Amino Acid Hydroxylase	0.901
4AX9	296	3.4.21.5	NA	Hydrolase/Hydrolase Inhibitor	Trypsin-Like Serine Proteases	Trypsin	0.901
4D0R	269	1.3.1.9	NA	Oxidoreductase	NA	Enoyl-(Acyl Carrier Protein) Reductase	0.906
4DZ5	331	1.-.-.-	NA	Oxidoreductase	Nadp-Dependent Oxidoreductase	Aldo/Keto Reductase Family	0.908
4E05	356	3.4.21.5	NA	Hydrolase/Hydrolase Inhibitor	Trypsin-Like Serine Proteases	Trypsin	0.916
4E97	374	3.2.1.17	NA	Hydrolase	NA	Phage Lysozyme	0.901
4EKP	374	3.2.1.17	NA	Hydrolase	NA	Phage Lysozyme	0.901
4FA3	331	1.-.-.-	NA	Oxidoreductase/Oxidoreductase Inhibitor	Nadp-Dependent Oxidoreductase	Aldo/Keto Reductase Family	0.925
4FAL	331	1.1.1.213	NA	Oxidoreductase/Oxidoreductase Inhibitor	Nadp-Dependent Oxidoreductase	Aldo/Keto Reductase Family	0.907
4FV0	360	2.7.11.24	NA	Transferase	Phosphorylase Kinase; Domain 1	Protein Kinase Domain	0.908
4FV9	360	2.7.11.24	NA	Transferase	Phosphorylase Kinase; Domain 1	Protein Kinase Domain	0.908
4G6N	360	2.7.11.24	NA	Transferase	Transferase(Phosphotransferase) Domain 1	Protein Kinase Domain	0.902
4GCA	315	1.1.1.21	NA	Oxidoreductase/Oxidoreductase Inhibitor	NA	Aldo/Keto Reductase Family	0.906
4H7C	331	1.-.-.-	NA	Oxidoreductase/Oxidoreductase Inhibitor	Nadp-Dependent Oxidoreductase	Aldo/Keto Reductase Family	0.908
4I7J	374	3.2.1.17	NA	Hydrolase	NA	Phage Lysozyme	0.902
4I7L	374	3.2.1.17	NA	Hydrolase	NA	Phage Lysozyme	0.903
4I7O	374	3.2.1.17	NA	Hydrolase	NA	Phage Lysozyme	0.901
4I7R	374	3.2.1.17	NA	Hydrolase	NA	Phage Lysozyme	0.903
4I7S	374	3.2.1.17	NA	Hydrolase	NA	Phage Lysozyme	0.903
4IGS	316	1.1.1.21	NA	Oxidoreductase/Oxidoreductase Inhibitor	NA	Aldo/Keto Reductase Family	0.913
4JII	318	1.1.1.-	NA	Oxidoreductase/Oxidoreductase Inhibitor	NA	Aldo/Keto Reductase Family	0.927
4JIR	316	1.1.1.21	NA	Oxidoreductase/Oxidoreductase Inhibitor	NA	Aldo/Keto Reductase Family	0.928
4LAU	316	1.1.1.21	NA	Oxidoreductase	NA	Aldo/Keto Reductase Family	0.905
4LAZ	316	1.1.1.21	NA	Oxidoreductase	NA	Aldo/Keto Reductase Family	0.904
4LB3	316	1.1.1.21	NA	Oxidoreductase	NA	Aldo/Keto Reductase Family	0.905
4LB4	316	1.1.1.21	NA	Oxidoreductase	NA	Aldo/Keto Reductase Family	0.904
4LBR	316	1.1.1.21	NA	Oxidoreductase	NA	Aldo/Keto Reductase Family	0.901
4LBS	316	1.1.1.21	NA	Oxidoreductase	NA	Aldo/Keto Reductase Family	0.902
4NKC	315	1.1.1.21	NA	Oxidoreductase/Oxidoreductase Inhibitor	NA	Aldo/Keto Reductase Family	0.915
4O6E	368	2.7.11.24	NA	Transferase/Transferase Inhibitor	NA	Protein Kinase Domain	0.903
4PR4	315	1.1.1.21	NA	Oxidoreductase/Oxidoreductase Inhibitor	NA	Aldo/Keto Reductase Family	0.917
4PRR	315	1.1.1.21	NA	Oxidoreductase/Oxidoreductase Inhibitor	NA	Aldo/Keto Reductase Family	0.920
4PRT	316	1.1.1.21	NA	Oxidoreductase/Oxidoreductase Inhibitor	NA	Aldo/Keto Reductase Family	0.911
4PUU	316	1.1.1.21	NA	Oxidoreductase/Oxidoreductase Inhibitor	NA	Aldo/Keto Reductase Family	0.911
4PUW	316	1.1.1.21	NA	Oxidoreductase/Oxidoreductase Inhibitor	NA	Aldo/Keto Reductase Family	0.914
4Q7B	315	1.1.1.21	NA	Oxidoreductase/Oxidoreductase Inhibitor	NA	Aldo/Keto Reductase Family	0.913
4QAH	299	3.1.3.48	NA	Hydrolase	NA	Protein-Tyrosine Phosphatase	0.914
4QAP	299	3.1.3.48	NA	Hydrolase	NA	Protein-Tyrosine Phosphatase	0.908
4QBE	298	3.1.3.48	NA	Hydrolase	NA	Protein-Tyrosine Phosphatase	0.905
4QBW	299	3.1.3.48	NA	Hydrolase	NA	Protein-Tyrosine Phosphatase	0.910
4QBX	316	1.1.1.21	NA	Oxidoreductase/Oxidoreductase Inhibitor	NA	Aldo/Keto Reductase Family	0.912
4QX4	316	1.1.1.21	NA	Oxidoreductase/Oxidoreductase Inhibitor	NA	Aldo/Keto Reductase Family	0.910
4RPQ	315	1.1.1.21	NA	Oxidoreductase/Oxidoreductase Inhibitor	NA	Aldo/Keto Reductase Family	0.916
4WRH	331	1.-.-.-	NA	Oxidoreductase/Oxidoreductase Inhibitor	NA	Aldo/Keto Reductase Family	0.909
4X0M	305	2.7.11.30	NA	Transferase	NA	Protein Kinase Domain	0.914
4X2F	305	2.7.11.30	NA	Transferase/Transferase Inhibitor	NA	Protein Kinase Domain	0.915

PDB ID	Residue count	EC number	SCOPE fold	Classification	CATH description	Protein Family Annotation	Mean persistent similary
4X2G	305	2.7.11.30	NA	Transferase/Transferase Inhibitor	NA	Protein Kinase Domain	0.909
4X2J	305	2.7.11.30	NA	Transferase	NA	Protein Kinase Domain	0.916
4X2K	305	2.7.11.30	NA	Transferase	NA	Protein Kinase Domain	0.916
4X2N	305	2.7.11.30	NA	Transferase	NA	Protein Kinase Domain	0.920
4XZI	316	1.1.1.21	NA	Oxidoreductase	NA	Aldo/Keto Reductase Family	0.905
4YS1	316	1.1.1.21	NA	Oxidoreductase	NA	Aldo/Keto Reductase Family	0.908
4YU1	316	1.1.1.21	NA	Oxidoreductase	NA	Aldo/Keto Reductase Family	0.924
4ZRT	309	3.1.3.48	NA	Hydrolase	NA	Protein-Tyrosine Phosphatase	0.906
4ZZN	350	2.7.11.24	NA	Transferase	NA	Protein Kinase Domain	0.906
5E8Z	307	2.7.11.30	NA	Transferase/Transferase Inhibitor	NA	Protein Kinase Domain	0.908
5GDS	321	3.4.21.5	NA	Hydrolase/Hydrolase Inhibitor	Trypsin-Like Serine Proteases	Trypsin	0.909
5GIM	325	3.4.21.5	NA	Hydrolase/Hydrolase Inhibitor	NA	Thrombin Light Chain	0.905
5JFD	307	3.4.21.5	NA	Hydrolase	NA	Trypsin	0.903
5KAA	289	3.1.3.48	NA	Hydrolase	NA	Protein-Tyrosine Phosphatase	0.909
5LUC	3224	2.6.1.51	NA	Transferase	NA	Aminotransferase Class-V	0.918
5NHH	381	2.7.11.24	NA	Transferase	NA	Protein Kinase Domain	0.901
5QIM	306	2.7.11.30	NA	Transferase/Transferase Inhibitor	NA	Protein Kinase Domain	0.908
5TC6	297	2.4.2.28	NA	Transferase/Transferase Inhibitor	NA	Phosphorylase Superfamily	0.900
5TC8	297	2.4.2.28	NA	Transferase/Transferase Inhibitor	NA	Phosphorylase Superfamily	0.904
5TE0	347	2.7.11.1	NA	Transferase/Transferase Inhibitor	NA	Protein Kinase Domain	0.904
5USQ	299	2.7.11.30	NA	Transferase/Transferase Inhibitor	NA	Protein Kinase Domain	0.910
5Y7N	316	1.1.1.-	NA	Oxidoreductase	NA	Aldo/Keto Reductase Family	0.912
6B8T	321	3.1.3.48	NA	Signaling Protein	NA	Protein-Tyrosine Phosphatase	0.903
6B8X	321	3.1.3.48	NA	Signaling Protein	NA	Protein-Tyrosine Phosphatase	0.902
6B8Y	307	2.7.11.30	NA	Transferase/Transferase Inhibitor	NA	Protein Kinase Domain	0.907
6F7R	316	1.1.1.21	NA	Oxidoreductase	NA	Aldo/Keto Reductase Family	0.900
6F81	316	1.1.1.21	NA	Oxidoreductase	NA	Aldo/Keto Reductase Family	0.904
6F82	316	1.1.1.21	NA	Oxidoreductase	NA	Aldo/Keto Reductase Family	0.913
6F84	316	1.1.1.21	NA	Oxidoreductase	NA	Aldo/Keto Reductase Family	0.916
6F8O	317	1.1.1.21	NA	Oxidoreductase	NA	Aldo/Keto Reductase Family	0.905
6G6J	354	NA	NA	Apoptosis	NA	Helix-Loop-Helix Dna-Binding Domain	0.905
6G8X	368	2.7.11.24	NA	Signaling Protein	NA	Protein Kinase Domain	0.906
6G91	368	2.7.11.24	NA	Signaling Protein	NA	Protein Kinase Domain	0.911
6G9A	368	2.7.11.24	NA	Signaling Protein	NA	Protein Kinase Domain	0.920
6G9K	368	2.7.11.24	NA	Signaling Protein	NA	Protein Kinase Domain	0.901
6G9M	368	2.7.11.24	NA	Signaling Protein	NA	Protein Kinase Domain	0.910
6NBS	367	2.7.11.24	NA	Transferase/Transferase Inhibitor	NA	Protein Kinase Domain	0.908
6OMY	297	3.1.3.48	NA	Hydrolase	NA	Protein-Tyrosine Phosphatase	0.902
6OPG	354	2.7.11.24	NA	Transferase	NA	Protein Kinase Domain	0.906
6OPI	354	2.7.11.24	NA	Transferase	NA	Protein Kinase Domain	0.907
6PFW	297	3.1.3.48	NA	Hydrolase	NA	Protein-Tyrosine Phosphatase	0.909
6PG0	297	3.1.3.48	NA	Hydrolase	NA	Protein-Tyrosine Phosphatase	0.901
6PHA	297	3.1.3.48	NA	Hydrolase	NA	Protein-Tyrosine Phosphatase	0.904
6PHS	297	3.1.3.48	NA	Hydrolase	NA	Protein-Tyrosine Phosphatase	0.908
6Q7S	368	2.7.11.24	NA	Signaling Protein	NA	Protein Kinase Domain	0.905
6Q7T	368	2.7.11.24	NA	Signaling Protein	NA	Protein Kinase Domain	0.911
6QA1	368	2.7.11.24	NA	Signaling Protein	NA	Protein Kinase Domain	0.921
6QA4	368	2.7.11.24	NA	Signaling Protein	NA	Protein Kinase Domain	0.909
6QAL	368	2.7.11.24	NA	Signaling Protein	NA	Protein Kinase Domain	0.924
6QAW	368	2.7.11.24	NA	Signaling Protein	NA	Protein Kinase Domain	0.902
6SLG	386	2.7.11.24	NA	Transferase	NA	Protein Kinase Domain	0.904

Supplementary Table 1: Proteins targeted by Drugbank FDA-approved medications showing average persistent similarity measures higher than 0.9 with 6M2Q.

Drug name	Drug id	PC set 1(GSE150316)	PC set 2 (CRA002390)	PC set 3 (GSE147507)	autodock LE (kcal/mol)	autodock cluster
**Cholic Acid**	DB02659	-0.08805090329	-0.1122360022	-0.08069352402	-15.06	74
**Rutin**	DB01698	-0.07057525739	-0.1847669519	-0.1024903315	-14.52	149
**Deoxycholic acid**	DB03619	-0.08557550853	-0.07828704382	-0.06475834161	-13.95	50
**Vorapaxar**	DB09030	NA	NA	NA	-13.57	38
**Indomethacin**	DB00328	-0.06872835755	-0.1234924169	-0.05359016853	-13.31	146
**Sulindac**	DB00605	-0.06768358761	-0.1193287796	-0.06631246593	-13.14	73
**NADH**	DB00157	NA	NA	NA	-13.11	19
**Argatroban**	DB00278	NA	NA	NA	-12.2	14
**Sulfaphenazole**	DB06729	-0.03586963775	-0.09303735598	-0.05246772731	-11.88	77
**Sulfisoxazole**	DB00263	-0.05362836277	-0.1325276338	-0.08602283924	-11.59	76
**Dequalinium**	DB04209	-0.07308190955	-0.02608396353	-0.02037787892	-11.35	15
**Pretomanid**	DB05154	-0.01941042793	-0.02603060121	-0.02876344372	-11.07	60
**Sulfacytine**	DB01298	NA	NA	NA	-11.03	80
**Dasatinib**	DB01254	-0.03959075682	-0.1464677954	-0.09185735897	-10.94	43
**Fostamatinib**	DB12010	NA	NA	NA	-10.74	11
**Triclosan**	DB08604	-0.1338300494	-0.0825734045	-0.03948117551	-10.71	69
**Thalidomide**	DB01041	-0.08657364237	-0.1593972746	-0.08730739799	-10.7	11
**Sulfamethizole**	DB00576	-0.02669528015	-0.009223741736	5.7299665057815e-05	-10.56	53

Drug name	Drug id	PC set 1(GSE150316)	PC set 2 (CRA002390)	PC set 3 (GSE147507)	autodock LE (kcal/mol)	autodock cluster
**Lactose**	DB04465	NA	NA	NA	-10.43	30
**Sulfamethoxazole**	DB01015	-0.01913885916	-0.003014737224	-0.004657065098	-10.39	50
**Sulfamethazine**	DB01582	-0.02788408235	-0.02028104527	-0.007299216869	-10.37	63
**Sulfamerazine**	DB01581	-0.03492115083	-0.01994952868	-0.05072835775	-9.97	44
**Sulfameter**	DB06821	NA	NA	NA	-9.94	62
**Menadione**	DB00170	-0.1453822115	-0.1355622276	-0.07575705865	-9.31	150
**Timolol**	DB00373	-0.06960236247	-0.1682405832	-0.08729867794	-9.18	12
**Glutathione**	DB00143	NA	NA	NA	-9.14	16
**Sulfacetamide**	DB00634	-0.04831995979	-0.1149364926	-0.08114789578	-9.13	94
**Proflavine**	DB01123	NA	NA	NA	-9.09	30
**Cladribine**	DB00242	-0.1176725341	-0.1384668748	-0.0553976742	-8.98	65
**Flufenamic acid**	DB02266	-0.03794344247	-0.1482318394	-0.0330936669	-8.92	35
**Isoprenaline**	DB01064	NA	NA	NA	-8.73	44
**Didanosine**	DB00900	-0.0523769169	-0.07471125925	-0.0586221941	-8.57	69
**Pyridoxal phosphate**	DB00114	NA	NA	NA	-8.4	58
**Droxidopa**	DB06262	NA	NA	NA	-8.4	55
**Sapropterin**	DB00360	NA	NA	NA	-8.25	66
**Dabigatran etexilate**	DB06695	NA	NA	NA	-8.16	4
**Triflusal**	DB08814	-0.05294840337	-0.1230937216	-0.07077350638	-7.57	39
**Sulfanilamide**	DB00259	NA	NA	NA	-7.52	128
**Norepinephrine**	DB00368	NA	NA	NA	-7.46	46
**Pseudoephedrine**	DB00852	NA	NA	NA	-7.3	42
**Phenylalanine**	DB00120	NA	NA	NA	-7.24	47
**Acetylsalicylic acid**	DB00945	-0.03307493015	-0.1012026588	-0.07444387473	-7.03	36

Drug name	Drug id	PC set 1(GSE150316)	PC set 2 (CRA002390)	PC set 3 (GSE147507)	autodock LE (kcal/mol)	autodock cluster
**Ximelagatran**	DB04898	NA	NA	NA	-7	16
**Ethionamide**	DB00609	-0.006951782285	-0.07256201732	-0.04149610657	-6.96	54
**Salicylic acid**	DB00936	NA	NA	NA	-6.34	59
**Adenine**	DB00173	NA	NA	NA	-6.29	49
**Isoniazid**	DB00951	-0.02183996932	-0.06238072939	-0.05362049256	-6.07	69
**Citric acid**	DB04272	NA	NA	NA	-5.83	28
**Phenol**	DB03255	NA	NA	NA	-5.48	150
**Taurine**	DB01956	NA	NA	NA	-4.92	81
**Guanidine**	DB00536	NA	NA	NA	-3.44	28
**Glycine**	DB00145	NA	NA	NA	-3.32	91
**Sucralfate**	DB00364	NA	NA	NA	15.17	75
**Bivalirudin**	DB00006	NA	NA	NA	4030	1

Supplementary Table 2: Transcriptomic and molecular docking analyses results for drugs with the potential of targeting the SARS-CoV-2 3CL protease in apo conformation (6M2Q)

PDB ID	Residue count	EC number	SCOPE fold	Classification	CATH description	Protein Family Annotation	Mean persistent similary
104L	332	NA	Lysozyme-Like	Hydrolase(O-Glycosyl)	NA	Phage Lysozyme	0.906
1A5Y	330	3.1.3.48	(Phosphotyrosine Protein) Phosphatases Ii	Hydrolase	Protein Tyrosine Phosphatase Superfamily	Protein-Tyrosine Phosphatase	0.909
1AAX	321	3.1.3.48	(Phosphotyrosine Protein) Phosphatases Ii	Hydrolase	Protein Tyrosine Phosphatase Superfamily	Protein-Tyrosine Phosphatase	0.907
1ABI	315	3.4.21.5	NA	Hydrolase/Hydrolase Inhibitor	Trypsin-Like Serine Proteases	Trypsin	0.908
1ADS	315	1.1.1.21	Tim Beta/Alpha-Barrel	Oxidoreductase	Nadp-Dependent Oxidoreductase	Aldo/Keto Reductase Family	0.902
1AE8	305	3.4.21.5	NA	Hydrolase/Hydrolase Inhibitor	Trypsin-Like Serine Proteases	Trypsin	0.905
1AFE	305	3.4.21.5	NA	Hydrolase/Hydrolase Inhibitor	Trypsin-Like Serine Proteases	Trypsin	0.912
1AI8	307	3.4.21.5	NA	Blood Coagulation/Hydrolase Inhibitor	Trypsin-Like Serine Proteases	Trypsin	0.902
1AJ0	282	2.5.1.15	Tim Beta/Alpha-Barrel	Synthase	Dihydropteroate (Dhp) Synthetase	Pterin Binding Enzyme	0.900
1AY6	308	3.4.21.5	NA	Hydrolase/Hydrolase Inhibitor	Trypsin-Like Serine Proteases	Trypsin	0.903
1AZ2	315	1.1.1.21	Tim Beta/Alpha-Barrel	Oxidoreductase	Nadp-Dependent Oxidoreductase	Aldo/Keto Reductase Family	0.915
1C1W	306	3.4.21.5	NA	Hydrolase/Hydrolase Inhibitor	Trypsin-Like Serine Proteases	Trypsin	0.900
1C4Y	308	3.4.21.5	Trypsin-Like Serine Proteases	Hydrolase/Hydrolase Inhibitor	NA	Thrombin Light Chain	0.900
1C5L	305	3.4.21.5	NA	Blood Clotting/Hydrolase Inhibitor	Trypsin-Like Serine Proteases	Trypsin	0.903
1C5N	305	3.4.21.5	NA	Hydrolase/Hydrolase Inhibitor	Trypsin-Like Serine Proteases	Trypsin	0.907
1D3T	307	3.4.21.5	Trypsin-Like Serine Proteases	Hydrolase/Hydrolase Inhibitor	NA	Thrombin Light Chain	0.902
1DM4	306	NA	Trypsin-Like Serine Proteases	Hydrolase	NA	Thrombin Light Chain	0.905
1DWC	306	3.4.21.5	NA	Hydrolase/Hyrolase Inhibitor	Trypsin-Like Serine Proteases	Trypsin	0.901
1E25	282	3.5.2.6	Beta-Lactamase/Transpeptidase-Like	Hydrolase	Dd-Peptidase/Beta-Lactamase Superfamily	Beta-Lactamase Enzyme Family	0.912
1EB1	297	NA	NA	Hydrolase/Hydrolase Inhibitor	NA	NA	0.904
1EEO	332	3.1.3.48	(Phosphotyrosine Protein) Phosphatases Ii	Hydrolase/Hydrolase Substrate	Protein Tyrosine Phosphatase Superfamily	Protein-Tyrosine Phosphatase	0.913
1EL3	316	1.1.1.21	Tim Beta/Alpha-Barrel	Oxidoreductase	Nadp-Dependent Oxidoreductase	Aldo/Keto Reductase Family	0.924
1ENY	268	NA	Nad(P)-Binding Rossmann-Fold Domains	Oxidoreductase	Nad(P)-Binding Rossmann-Like Domain	Enoyl-(Acyl Carrier Protein) Reductase	0.906
1G30	306	3.4.21.5	Trypsin-Like Serine Proteases	Hydrolase/Hydrolase Inhibitor	NA	Thrombin Light Chain	0.909
1G32	306	3.4.21.5	Trypsin-Like Serine Proteases	Hydrolase/Hydrolase Inhibitor	NA	Thrombin Light Chain	0.906
1GHV	304	3.4.21.5	NA	Hydrolase/Hydrolase Inhibitor	Trypsin-Like Serine Proteases	Trypsin	0.910
1GJ5	305	3.4.21.5	NA	Blood Clotting,Hydrolase/Inhibitor	Trypsin-Like Serine Proteases	Trypsin	0.901
1H8I	290	3.4.21.5	NA	Hydrolase/Hydrolase Inhibitor	Trypsin-Like Serine Proteases	Trypsin	0.903
1HAH	305	3.4.21.5	NA	Complex(Serine Proteinase/Inhibitor)	Trypsin-Like Serine Proteases	Trypsin	0.912
1HAI	295	3.4.21.5	NA	Hydrolase/Hydrolase Inhibitor	Trypsin-Like Serine Proteases	Trypsin	0.904
1HDT	304	3.4.21.5	NA	Hydrolase/Hydrolase Inhibitor	Trypsin-Like Serine Proteases	Trypsin	0.910
1IHS	316	3.4.21.5	NA	Hydrolase/Hydrolase Inhibitor	Trypsin-Like Serine Proteases	Trypsin	0.912
1JWT	305	3.4.21.5	Trypsin-Like Serine Proteases	Hydrolase	Trypsin-Like Serine Proteases	Thrombin Light Chain	0.904
1KAV	298	3.1.3.48	(Phosphotyrosine Protein) Phosphatases Ii	Hydrolase	Protein Tyrosine Phosphatase Superfamily	Protein-Tyrosine Phosphatase	0.903
1KW0	325	1.14.16.1	Aromatic Aminoacid Monoxygenases, Catalytic And Oligomerization Domains	Oxidoreductase	Phenylalanine Hydroxylase	Biopterin-Dependent Aromatic Amino Acid Hydroxylase	0.907
1MAR	315	1.1.1.21	Tim Beta/Alpha-Barrel	Oxidoreductase(Nadp)	NA	Aldo/Keto Reductase Family	0.921
1MMK	325	1.14.16.1	Aromatic Aminoacid Monoxygenases, Catalytic And Oligomerization Domains	Oxidoreductase	Phenylalanine Hydroxylase	Biopterin-Dependent Aromatic Amino Acid Hydroxylase	0.921
1MMT	325	1.14.16.1	Aromatic Aminoacid Monoxygenases, Catalytic And Oligomerization Domains	Oxidoreductase	Phenylalanine Hydroxylase	Biopterin-Dependent Aromatic Amino Acid Hydroxylase	0.906
1MRQ	323	1.1.1.149	Tim Beta/Alpha-Barrel	Oxidoreductase	Nadp-Dependent Oxidoreductase	Aldo/Keto Reductase Family	0.904
1MUE	306	3.4.21.5	Trypsin-Like Serine Proteases	Hydrolase Inhibitor	NA	Thrombin Light Chain	0.905
1NO9	306	3.4.21.5	NA	Hydrolase/Hydrolase Inhibitor	Trypsin-Like Serine Proteases	Trypsin	0.901
1NRN	318	3.4.21.5	NA	Serine Proteinase/Receptor	Trypsin-Like Serine Proteases	Trypsin	0.907
1NRS	311	3.4.21.5	NA	Hydrolase/Hydrolase Inhibitor	Trypsin-Like Serine Proteases	Trypsin	0.905
1NW9	375	NA	Inhibitor Of Apoptosis (Iap) Repeat	Apoptosis	Inhibitor Of Apoptosis Protein (2mihbc-Iap-1); Chain A	Inhibitor Of Apoptosis Domain	0.901
1OES	321	3.1.3.48	(Phosphotyrosine Protein) Phosphatases Ii	Hydrolase	Protein Tyrosine Phosphatase Superfamily	Protein-Tyrosine Phosphatase	0.902
1OEV	321	3.1.3.48	(Phosphotyrosine Protein) Phosphatases Ii	Hydrolase	Protein Tyrosine Phosphatase Superfamily	Protein-Tyrosine Phosphatase	0.901
1ONY	321	3.1.3.48	(Phosphotyrosine Protein) Phosphatases Ii	Hydrolase	Protein Tyrosine Phosphatase Superfamily	Protein-Tyrosine Phosphatase	0.915
1PF7	289	2.4.2.1	Phosphorylase/Hydrolase-Like	Transferase	NA	Phosphorylase Superfamily	0.912
1PME	380	2.7.1.-	Protein Kinase-Like (Pk-Like)	Transferase	Phosphorylase Kinase; Domain 1	Protein Kinase Domain	0.916
1PMU	364	2.7.1.-	Protein Kinase-Like (Pk-Like)	Transferase	Phosphorylase Kinase; Domain 1	Protein Kinase Domain	0.911
1PTT	326	3.1.3.48	(Phosphotyrosine Protein) Phosphatases Ii	Complex (Hydrolase/Peptide)	Protein Tyrosine Phosphatase Superfamily	Protein-Tyrosine Phosphatase	0.911
1PTV	321	3.1.3.48	(Phosphotyrosine Protein) Phosphatases Ii	Complex (Hydrolase/Peptide)	Protein Tyrosine Phosphatase Superfamily	Protein-Tyrosine Phosphatase	0.905
1PTY	321	3.1.3.48	(Phosphotyrosine Protein) Phosphatases Ii	Hydrolase	Protein Tyrosine Phosphatase Superfamily	Protein-Tyrosine Phosphatase	0.902
1PWL	316	1.1.1.21	Tim Beta/Alpha-Barrel	Oxidoreductase	Nadp-Dependent Oxidoreductase	Aldo/Keto Reductase Family	0.910
1PWM	316	1.1.1.21	Tim Beta/Alpha-Barrel	Oxidoreductase	Nadp-Dependent Oxidoreductase	Aldo/Keto Reductase Family	0.920
1PWY	288	2.4.2.1	Phosphorylase/Hydrolase-Like	Transferase	NA	Phosphorylase Superfamily	0.902
1QBV	306	NA	NA	Hydrolase/Hydrolase Inhibitor	NA	NA	0.900
1QHR	305	3.4.21.5	Trypsin-Like Serine Proteases	Blood Clotting/Hydrolase Inhibitor	NA	Thrombin Light Chain	0.900
1QUR	303	NA	NA	Hydrolase/Hydrolase Inhibitor	Trypsin-Like Serine Proteases	Trypsin	0.906
1RCT	288	2.4.2.1	Phosphorylase/Hydrolase-Like	Transferase	NA	Phosphorylase Superfamily	0.903
1RW8	301	2.7.1.37	NA	Transferase	Transferase(Phosphotransferase) Domain 1	Protein Kinase Domain	0.913
1S2A	331	1.1.1.213	Tim Beta/Alpha-Barrel	Oxidoreductase	Nadp-Dependent Oxidoreductase	Aldo/Keto Reductase Family	0.905
1S2C	331	1.1.1.213	Tim Beta/Alpha-Barrel	Oxidoreductase	Nadp-Dependent Oxidoreductase	Aldo/Keto Reductase Family	0.904
1SVC	384	NA	NA	Transcription/Dna	NA	NA	0.906
1T40	316	1.1.1.21	Tim Beta/Alpha-Barrel	Oxidoreductase	Nadp-Dependent Oxidoreductase	Aldo/Keto Reductase Family	0.916
1T48	298	3.1.3.48	(Phosphotyrosine Protein) Phosphatases Ii	Hydrolase	Protein Tyrosine Phosphatase Superfamily	Protein-Tyrosine Phosphatase	0.906
1THR	308	3.4.21.5	NA	Hydrolase(Serine Proteinase)	Trypsin-Like Serine Proteases	Trypsin	0.903
1TMB	313	3.4.21.5	NA	Hydrolase/Hydrolase Inhibitor	Trypsin-Like Serine Proteases	Trypsin	0.905
1TMT	316	3.4.21.5	NA	Hydrolase/Hydrolase Inhibitor	Trypsin-Like Serine Proteases	Trypsin	0.909
1UMA	305	3.4.21.5	NA	Hydrolase/Hydrolase Inhibitor	Trypsin-Like Serine Proteases	Trypsin	0.914
1V41	288	2.4.2.1	Phosphorylase/Hydrolase-Like	Transferase	NA	Phosphorylase Superfamily	0.921
1VJY	303	2.7.1.37	Protein Kinase-Like (Pk-Like)	Transferase	Phosphorylase Kinase; Domain 1	Protein Kinase Domain	0.931
1WAY	305	3.4.21.5	NA	Hydrolase/Hydrolase Inhibitor	NA	Thrombin Light Chain	0.903
1WBG	305	3.4.21.5	NA	Hydrolase/Hydrolase Inhibitor	NA	Thrombin Light Chain	0.905
1X96	316	1.1.1.21	Tim Beta/Alpha-Barrel	Oxidoreductase	Nadp-Dependent Oxidoreductase	Aldo/Keto Reductase Family	0.921
1X97	316	1.1.1.21	Tim Beta/Alpha-Barrel	Oxidoreductase	Nadp-Dependent Oxidoreductase	Aldo/Keto Reductase Family	0.916
1X98	316	1.1.1.21	Tim Beta/Alpha-Barrel	Oxidoreductase	Nadp-Dependent Oxidoreductase	Aldo/Keto Reductase Family	0.912
1XAS	299	3.2.1.8	Tim Beta/Alpha-Barrel	Xylanase A	NA	Glycosyl Hydrolase Family 10	0.921
1XM1	305	3.4.21.5	Trypsin-Like Serine Proteases	Hydrolase/Hydrolase Inhibitor	Trypsin-Like Serine Proteases	Trypsin	0.900
1YRY	289	2.4.2.1	Phosphorylase/Hydrolase-Like	Transferase	NA	Phosphorylase Superfamily	0.902
1Z3N	319	1.1.1.21	Tim Beta/Alpha-Barrel	Oxidoreductase	Nadp-Dependent Oxidoreductase	Aldo/Keto Reductase Family	0.930
1Z89	316	1.1.1.21	Tim Beta/Alpha-Barrel	Oxidoreductase	Nadp-Dependent Oxidoreductase	Aldo/Keto Reductase Family	0.921
1Z8A	316	1.1.1.21	Tim Beta/Alpha-Barrel	Oxidoreductase	Nadp-Dependent Oxidoreductase	Aldo/Keto Reductase Family	0.905
1ZUA	317	1.1.1.-	NA	Oxidoreductase	Nadp-Dependent Oxidoreductase	Aldo/Keto Reductase Family	0.913

PDB ID	Residue count	EC number	SCOPE fold	Classification	CATH description	Protein Family Annotation	Mean persistent similary
2ACQ	315	1.1.1.21	Tim Beta/Alpha-Barrel	Oxidoreductase	Nadp-Dependent Oxidoreductase	Aldo/Keto Reductase Family	0.905
2ACR	315	1.1.1.21	Tim Beta/Alpha-Barrel	Oxidoreductase	Nadp-Dependent Oxidoreductase	Aldo/Keto Reductase Family	0.902
2ACU	315	1.1.1.21	Tim Beta/Alpha-Barrel	Oxidoreductase	Nadp-Dependent Oxidoreductase	Aldo/Keto Reductase Family	0.921
2AGT	319	1.1.1.21	NA	Oxidoreductase	Nadp-Dependent Oxidoreductase	Aldo/Keto Reductase Family	0.915
2BGD	321	3.1.3.48	(Phosphotyrosine Protein) Phosphatases Ii	Hydrolase	Protein Tyrosine Phosphatase Superfamily	Protein-Tyrosine Phosphatase	0.900
2BGE	321	3.1.3.48	(Phosphotyrosine Protein) Phosphatases Ii	Hydrolase	Protein Tyrosine Phosphatase Superfamily	Protein-Tyrosine Phosphatase	0.912
2C93	307	3.4.21.5	NA	Hydrolase/Hydrolase Inhibitor	NA	Thrombin Light Chain	0.903
2CMA	327	3.1.3.48	(Phosphotyrosine Protein) Phosphatases Ii	Hydrolase	Protein Tyrosine Phosphatase Superfamily	Protein-Tyrosine Phosphatase	0.905
2DUZ	316	1.1.1.21	Tim Beta/Alpha-Barrel	Oxidoreductase	Nadp-Dependent Oxidoreductase	Aldo/Keto Reductase Family	0.902
2DV0	316	1.1.1.21	Tim Beta/Alpha-Barrel	Oxidoreductase	Nadp-Dependent Oxidoreductase	Aldo/Keto Reductase Family	0.910
2F2K	316	1.1.1.21	Tim Beta/Alpha-Barrel	Oxidoreductase	Nadp-Dependent Oxidoreductase	Aldo/Keto Reductase Family	0.911
2F6F	302	3.1.3.48	NA	Hydrolase	Protein Tyrosine Phosphatase Superfamily	Protein-Tyrosine Phosphatase	0.902
2FGB	323	1.1.1.62	Tim Beta/Alpha-Barrel	Oxidoreductase	Nadp-Dependent Oxidoreductase	Aldo/Keto Reductase Family	0.906
2FZ8	316	1.1.1.21	Tim Beta/Alpha-Barrel	Oxidoreductase	Nadp-Dependent Oxidoreductase	Aldo/Keto Reductase Family	0.906
2FZ9	316	1.1.1.21	Tim Beta/Alpha-Barrel	Oxidoreductase	Nadp-Dependent Oxidoreductase	Aldo/Keto Reductase Family	0.915
2FZB	316	1.1.1.21	Tim Beta/Alpha-Barrel	Oxidoreductase	Nadp-Dependent Oxidoreductase	Aldo/Keto Reductase Family	0.920
2G3J	313	3.2.1.8	NA	Hydrolase	Glycosidases	Glycosyl Hydrolase Family 10	0.915
2GGT	328	NA	Thioredoxin Fold	Chaperone	Glutaredoxin	Sco1/Senc	0.927
2H4G	299	3.1.3.48	(Phosphotyrosine Protein) Phosphatases Ii	Hydrolase	Protein Tyrosine Phosphatase Superfamily	Protein-Tyrosine Phosphatase	0.902
2HV5	316	1.1.1.21	Tim Beta/Alpha-Barrel	Oxidoreductase	Nadp-Dependent Oxidoreductase	Aldo/Keto Reductase Family	0.913
2HVN	316	1.1.1.21	Tim Beta/Alpha-Barrel	Oxidoreductase	Nadp-Dependent Oxidoreductase	Aldo/Keto Reductase Family	0.918
2HVO	316	1.1.1.21	Tim Beta/Alpha-Barrel	Oxidoreductase	Nadp-Dependent Oxidoreductase	Aldo/Keto Reductase Family	0.912
2IKG	316	1.1.1.21	Tim Beta/Alpha-Barrel	Oxidoreductase	Nadp-Dependent Oxidoreductase	Aldo/Keto Reductase Family	0.916
2IKH	316	1.1.1.21	Tim Beta/Alpha-Barrel	Oxidoreductase	Nadp-Dependent Oxidoreductase	Aldo/Keto Reductase Family	0.914
2IKJ	316	1.1.1.21	Tim Beta/Alpha-Barrel	Oxidoreductase	Nadp-Dependent Oxidoreductase	Aldo/Keto Reductase Family	0.905
2INE	315	1.1.1.21	Tim Beta/Alpha-Barrel	Oxidoreductase	Nadp-Dependent Oxidoreductase	Aldo/Keto Reductase Family	0.909
2INZ	315	1.1.1.21	Tim Beta/Alpha-Barrel	Oxidoreductase	Nadp-Dependent Oxidoreductase	Aldo/Keto Reductase Family	0.903
2IQ0	315	1.1.1.21	Tim Beta/Alpha-Barrel	Oxidoreductase	Nadp-Dependent Oxidoreductase	Aldo/Keto Reductase Family	0.909
2IQD	315	1.1.1.21	Tim Beta/Alpha-Barrel	Oxidoreductase	Nadp-Dependent Oxidoreductase	Aldo/Keto Reductase Family	0.909
2ISF	315	1.1.1.21	Tim Beta/Alpha-Barrel	Oxidoreductase	Nadp-Dependent Oxidoreductase	Aldo/Keto Reductase Family	0.905
2J8T	316	1.1.1.21	Tim Beta/Alpha-Barrel	Oxidoreductase	Nadp-Dependent Oxidoreductase	Aldo/Keto Reductase Family	0.903
2NVC	316	1.1.1.21	Tim Beta/Alpha-Barrel	Oxidoreductase	Nadp-Dependent Oxidoreductase	Aldo/Keto Reductase Family	0.913
2PD5	316	1.1.1.21	NA	Oxidoreductase	Nadp-Dependent Oxidoreductase	Aldo/Keto Reductase Family	0.928
2PD9	316	1.1.1.21	NA	Oxidoreductase	Nadp-Dependent Oxidoreductase	Aldo/Keto Reductase Family	0.907
2PDB	316	1.1.1.21	NA	Oxidoreductase	Nadp-Dependent Oxidoreductase	Aldo/Keto Reductase Family	0.918
2PDC	316	1.1.1.21	NA	Oxidoreductase	Nadp-Dependent Oxidoreductase	Aldo/Keto Reductase Family	0.923
2PDF	316	1.1.1.21	NA	Oxidoreductase	Nadp-Dependent Oxidoreductase	Aldo/Keto Reductase Family	0.924
2PDG	316	1.1.1.21	Tim Beta/Alpha-Barrel	Oxidoreductase	Nadp-Dependent Oxidoreductase	Aldo/Keto Reductase Family	0.909
2PDH	316	1.1.1.21	NA	Oxidoreductase	Nadp-Dependent Oxidoreductase	Aldo/Keto Reductase Family	0.907
2PDI	316	1.1.1.21	NA	Oxidoreductase	Nadp-Dependent Oxidoreductase	Aldo/Keto Reductase Family	0.912
2PDJ	316	1.1.1.21	NA	Oxidoreductase	Nadp-Dependent Oxidoreductase	Aldo/Keto Reductase Family	0.903
2PDK	316	1.1.1.21	NA	Oxidoreductase	Nadp-Dependent Oxidoreductase	Aldo/Keto Reductase Family	0.912
2PDL	316	1.1.1.21	NA	Oxidoreductase	Nadp-Dependent Oxidoreductase	Aldo/Keto Reductase Family	0.917
2PDN	316	1.1.1.21	NA	Oxidoreductase	Nadp-Dependent Oxidoreductase	Aldo/Keto Reductase Family	0.927
2PDP	316	1.1.1.21	NA	Oxidoreductase	Nadp-Dependent Oxidoreductase	Aldo/Keto Reductase Family	0.914
2PDQ	316	1.1.1.21	NA	Oxidoreductase	Nadp-Dependent Oxidoreductase	Aldo/Keto Reductase Family	0.904
2PDW	316	1.1.1.21	NA	Oxidoreductase	Nadp-Dependent Oxidoreductase	Aldo/Keto Reductase Family	0.904
2PEV	316	1.1.1.21	Tim Beta/Alpha-Barrel	Oxidoreductase	Nadp-Dependent Oxidoreductase	Aldo/Keto Reductase Family	0.903
2PGQ	304	3.4.21.5	NA	Hydrolase/Hydrolase Inhibitor	Epsilon-Thrombin, Subunit L	Thrombin Light Chain	0.902
2PZN	316	1.1.1.21	Tim Beta/Alpha-Barrel	Oxidoreductase	Nadp-Dependent Oxidoreductase	Aldo/Keto Reductase Family	0.925
2Q0N	312	2.7.11.1	NA	Transferase	Phosphorylase Kinase; Domain 1	Protein Kinase Domain	0.902
2QBR	299	3.1.3.48	NA	Hydrolase	Protein Tyrosine Phosphatase Superfamily	Protein-Tyrosine Phosphatase	0.900
2R24	316	1.1.1.21	NA	Oxidoreductase	Nadp-Dependent Oxidoreductase	Aldo/Keto Reductase Family	0.927
2RLC	328	3.5.1.24	NA	Hydrolase	Penicillin V Acylase; Chain A	Linear Amide C-N Hydrolases, Choloylglycine Hydrolase Family	0.910
2WOT	306	2.7.11.30	NA	Transferase	Transferase(Phosphotransferase) Domain 1	Protein Kinase Domain	0.901
2WOU	306	2.7.11.30	NA	Transferase	Phosphorylase Kinase; Domain 1	Protein Kinase Domain	0.917
3BCJ	316	1.1.1.21	Tim Beta/Alpha-Barrel	Oxidoreductase	Nadp-Dependent Oxidoreductase	Aldo/Keto Reductase Family	0.903
3C3U	323	1.1.1.149	Tim Beta/Alpha-Barrel	Oxidoreductase	Nadp-Dependent Oxidoreductase	Aldo/Keto Reductase Family	0.906
3D1V	289	2.4.2.1	NA	Transferase	NA	Phosphorylase Superfamily	0.903
3D42	316	3.1.3.48	NA	Hydrolase	Protein Tyrosine Phosphatase Superfamily	Protein-Tyrosine Phosphatase	0.930
3D9C	321	3.1.3.48	NA	Hydrolase	Protein Tyrosine Phosphatase Superfamily	Protein-Tyrosine Phosphatase	0.914
3DN5	316	1.1.1.21	NA	Oxidoreductase	Nadp-Dependent Oxidoreductase	Aldo/Keto Reductase Family	0.921
3FMN	372	2.7.11.24	NA	Signaling Protein, Transferase	Phosphorylase Kinase; Domain 1	Protein Kinase Domain	0.906
3G5E	319	1.1.1.21	NA	Oxidoreductase	Nadp-Dependent Oxidoreductase	Aldo/Keto Reductase Family	0.919
3GHR	316	1.1.1.21	NA	Oxidoreductase	Nadp-Dependent Oxidoreductase	Aldo/Keto Reductase Family	0.901
3GHS	316	1.1.1.21	NA	Oxidoreductase	Nadp-Dependent Oxidoreductase	Aldo/Keto Reductase Family	0.904
3GHT	316	1.1.1.21	NA	Oxidoreductase	Nadp-Dependent Oxidoreductase	Aldo/Keto Reductase Family	0.912
3GHU	316	1.1.1.21	NA	Oxidoreductase	Nadp-Dependent Oxidoreductase	Aldo/Keto Reductase Family	0.915
3GUG	323	1.1.1.-	NA	Oxidoreductase	Nadp-Dependent Oxidoreductase	Aldo/Keto Reductase Family	0.903
3HMM	303	2.7.11.30	NA	Transferase	Transferase(Phosphotransferase) Domain 1	Protein Kinase Domain	0.912
3HRB	359	2.7.11.24	NA	Transferase	Phosphorylase Kinase; Domain 1	Protein Kinase Domain	0.915
3INY	289	2.4.2.1	NA	Transferase	NA	Phosphorylase Superfamily	0.924
3LEN	316	1.1.1.21	NA	Oxidoreductase/Oxidoreductase Inhibitor	Nadp-Dependent Oxidoreductase	Aldo/Keto Reductase Family	0.916
3M0I	316	1.1.1.21	NA	Oxidoreductase/Oxidoreductase Inhibitor	Nadp-Dependent Oxidoreductase	Aldo/Keto Reductase Family	0.910
3M4H	316	1.1.1.21	NA	Oxidoreductase/Oxidoreductase Inhibitor	Nadp-Dependent Oxidoreductase	Aldo/Keto Reductase Family	0.907
3M64	316	1.1.1.21	NA	Oxidoreductase/Oxidoreductase Inhibitor	Nadp-Dependent Oxidoreductase	Aldo/Keto Reductase Family	0.909
3MB9	316	1.1.1.21	NA	Oxidoreductase/Oxidoreductase Inhibitor	Nadp-Dependent Oxidoreductase	Aldo/Keto Reductase Family	0.919
3MC5	316	1.1.1.21	NA	Oxidoreductase/Oxidoreductase Inhibitor	Nadp-Dependent Oxidoreductase	Aldo/Keto Reductase Family	0.915
3MFR	351	2.7.11.1	NA	Transferase	Phosphorylase Kinase; Domain 1	Protein Kinase Domain	0.911
3MFS	351	2.7.11.1	NA	Transferase	Phosphorylase Kinase; Domain 1	Protein Kinase Domain	0.912
3MFT	351	2.7.11.1	NA	Transferase	Transferase(Phosphotransferase) Domain 1	Protein Kinase Domain	0.903
3MFU	351	2.7.11.1	NA	Transferase	Phosphorylase Kinase; Domain 1	Protein Kinase Domain	0.917
3P2V	316	1.1.1.21	NA	Oxidoreductase/Oxidoreductase Inhibitor	Nadp-Dependent Oxidoreductase	Aldo/Keto Reductase Family	0.921
3QKP	321	3.1.3.48	NA	Hydrolase	Protein Tyrosine Phosphatase Superfamily	Protein-Tyrosine Phosphatase	0.902

PDB ID	Residue count	EC number	SCOPE fold	Classification	CATH description	Protein Family Annotation	Mean persistent similary
3R43	331	1.-.-.-	NA	Oxidoreductase/Oxidoreductase Inhibitor	Nadp-Dependent Oxidoreductase	Aldo/Keto Reductase Family	0.907
3R58	331	1.-.-.-	NA	Oxidoreductase/Oxidoreductase Inhibitor	Nadp-Dependent Oxidoreductase	Aldo/Keto Reductase Family	0.911
3R6I	331	1.-.-.-	NA	Oxidoreductase/Oxidoreductase Inhibitor	Nadp-Dependent Oxidoreductase	Aldo/Keto Reductase Family	0.903
3R7M	331	1.1.1.213	NA	Oxidoreductase/Oxidoreductase Inhibitor	Nadp-Dependent Oxidoreductase	Aldo/Keto Reductase Family	0.926
3R8G	331	1.-.-.-	NA	Oxidoreductase/Oxidoreductase Inhibitor	Nadp-Dependent Oxidoreductase	Aldo/Keto Reductase Family	0.931
3RX2	336	1.1.1.21	NA	Oxidoreductase	Nadp-Dependent Oxidoreductase	Aldo/Keto Reductase Family	0.907
3RX3	336	1.1.1.21	NA	Oxidoreductase	Nadp-Dependent Oxidoreductase	Aldo/Keto Reductase Family	0.914
3RX4	336	1.1.1.21	NA	Oxidoreductase	Nadp-Dependent Oxidoreductase	Aldo/Keto Reductase Family	0.924
3S3G	336	1.1.1.21	NA	Oxidoreductase	Nadp-Dependent Oxidoreductase	Aldo/Keto Reductase Family	0.914
3T42	319	1.1.1.21	NA	Oxidoreductase/Oxidoreductase Inhibitor	Nadp-Dependent Oxidoreductase	Aldo/Keto Reductase Family	0.905
3U2C	316	1.1.1.21	NA	Oxidoreductase/Oxidoreductase Inhibitor	Nadp-Dependent Oxidoreductase	Aldo/Keto Reductase Family	0.910
3UFY	331	1.1.1.213	NA	Oxidoreductase	Nadp-Dependent Oxidoreductase	Aldo/Keto Reductase Family	0.924
3UG8	331	1.1.1.213	NA	Oxidoreductase	Nadp-Dependent Oxidoreductase	Aldo/Keto Reductase Family	0.913
3UGR	331	1.1.1.213	NA	Oxidoreductase	Nadp-Dependent Oxidoreductase	Aldo/Keto Reductase Family	0.922
3UWE	331	1.1.1.213	NA	Oxidoreductase	Nadp-Dependent Oxidoreductase	Aldo/Keto Reductase Family	0.901
3V35	336	1.1.1.21	NA	Oxidoreductase	Nadp-Dependent Oxidoreductase	Aldo/Keto Reductase Family	0.917
3V36	336	1.1.1.21	NA	Oxidoreductase	Nadp-Dependent Oxidoreductase	Aldo/Keto Reductase Family	0.902
3VXF	315	3.4.21.5	NA	Hydrolase/Hydrolase Inhibitor	NA	Trypsin	0.907
4ANP	324	1.14.16.1	NA	Oxidoreductase	Phenylalanine Hydroxylase	Biopterin-Dependent Aromatic Amino Acid Hydroxylase	0.901
4AX9	296	3.4.21.5	NA	Hydrolase/Hydrolase Inhibitor	Trypsin-Like Serine Proteases	Trypsin	0.901
4D0R	269	1.3.1.9	NA	Oxidoreductase	NA	Enoyl-(Acyl Carrier Protein) Reductase	0.906
4DZ5	331	1.-.-.-	NA	Oxidoreductase	Nadp-Dependent Oxidoreductase	Aldo/Keto Reductase Family	0.908
4E05	356	3.4.21.5	NA	Hydrolase/Hydrolase Inhibitor	Trypsin-Like Serine Proteases	Trypsin	0.916
4E97	374	3.2.1.17	NA	Hydrolase	NA	Phage Lysozyme	0.901
4EKP	374	3.2.1.17	NA	Hydrolase	NA	Phage Lysozyme	0.901
4FA3	331	1.-.-.-	NA	Oxidoreductase/Oxidoreductase Inhibitor	Nadp-Dependent Oxidoreductase	Aldo/Keto Reductase Family	0.925
4FAL	331	1.1.1.213	NA	Oxidoreductase/Oxidoreductase Inhibitor	Nadp-Dependent Oxidoreductase	Aldo/Keto Reductase Family	0.907
4FV0	360	2.7.11.24	NA	Transferase	Phosphorylase Kinase; Domain 1	Protein Kinase Domain	0.908
4FV9	360	2.7.11.24	NA	Transferase	Phosphorylase Kinase; Domain 1	Protein Kinase Domain	0.908
4G6N	360	2.7.11.24	NA	Transferase	Transferase(Phosphotransferase) Domain 1	Protein Kinase Domain	0.902
4GCA	315	1.1.1.21	NA	Oxidoreductase/Oxidoreductase Inhibitor	NA	Aldo/Keto Reductase Family	0.906
4H7C	331	1.-.-.-	NA	Oxidoreductase/Oxidoreductase Inhibitor	Nadp-Dependent Oxidoreductase	Aldo/Keto Reductase Family	0.908
4I7J	374	3.2.1.17	NA	Hydrolase	NA	Phage Lysozyme	0.902
4I7L	374	3.2.1.17	NA	Hydrolase	NA	Phage Lysozyme	0.903
4I7O	374	3.2.1.17	NA	Hydrolase	NA	Phage Lysozyme	0.901
4I7R	374	3.2.1.17	NA	Hydrolase	NA	Phage Lysozyme	0.903
4I7S	374	3.2.1.17	NA	Hydrolase	NA	Phage Lysozyme	0.903
4IGS	316	1.1.1.21	NA	Oxidoreductase/Oxidoreductase Inhibitor	NA	Aldo/Keto Reductase Family	0.913
4JII	318	1.1.1.-	NA	Oxidoreductase/Oxidoreductase Inhibitor	NA	Aldo/Keto Reductase Family	0.927
4JIR	316	1.1.1.21	NA	Oxidoreductase/Oxidoreductase Inhibitor	NA	Aldo/Keto Reductase Family	0.928
4LAU	316	1.1.1.21	NA	Oxidoreductase	NA	Aldo/Keto Reductase Family	0.905
4LAZ	316	1.1.1.21	NA	Oxidoreductase	NA	Aldo/Keto Reductase Family	0.904
4LB3	316	1.1.1.21	NA	Oxidoreductase	NA	Aldo/Keto Reductase Family	0.905
4LB4	316	1.1.1.21	NA	Oxidoreductase	NA	Aldo/Keto Reductase Family	0.904
4LBR	316	1.1.1.21	NA	Oxidoreductase	NA	Aldo/Keto Reductase Family	0.901
4LBS	316	1.1.1.21	NA	Oxidoreductase	NA	Aldo/Keto Reductase Family	0.902
4NKC	315	1.1.1.21	NA	Oxidoreductase/Oxidoreductase Inhibitor	NA	Aldo/Keto Reductase Family	0.915
4O6E	368	2.7.11.24	NA	Transferase/Transferase Inhibitor	NA	Protein Kinase Domain	0.903
4PR4	315	1.1.1.21	NA	Oxidoreductase/Oxidoreductase Inhibitor	NA	Aldo/Keto Reductase Family	0.917
4PRR	315	1.1.1.21	NA	Oxidoreductase/Oxidoreductase Inhibitor	NA	Aldo/Keto Reductase Family	0.920
4PRT	316	1.1.1.21	NA	Oxidoreductase/Oxidoreductase Inhibitor	NA	Aldo/Keto Reductase Family	0.911
4PUU	316	1.1.1.21	NA	Oxidoreductase/Oxidoreductase Inhibitor	NA	Aldo/Keto Reductase Family	0.911
4PUW	316	1.1.1.21	NA	Oxidoreductase/Oxidoreductase Inhibitor	NA	Aldo/Keto Reductase Family	0.914
4Q7B	315	1.1.1.21	NA	Oxidoreductase/Oxidoreductase Inhibitor	NA	Aldo/Keto Reductase Family	0.913
4QAH	299	3.1.3.48	NA	Hydrolase	NA	Protein-Tyrosine Phosphatase	0.914
4QAP	299	3.1.3.48	NA	Hydrolase	NA	Protein-Tyrosine Phosphatase	0.908
4QBE	298	3.1.3.48	NA	Hydrolase	NA	Protein-Tyrosine Phosphatase	0.905
4QBW	299	3.1.3.48	NA	Hydrolase	NA	Protein-Tyrosine Phosphatase	0.910
4QBX	316	1.1.1.21	NA	Oxidoreductase/Oxidoreductase Inhibitor	NA	Aldo/Keto Reductase Family	0.912
4QX4	316	1.1.1.21	NA	Oxidoreductase/Oxidoreductase Inhibitor	NA	Aldo/Keto Reductase Family	0.910
4RPQ	315	1.1.1.21	NA	Oxidoreductase/Oxidoreductase Inhibitor	NA	Aldo/Keto Reductase Family	0.916
4WRH	331	1.-.-.-	NA	Oxidoreductase/Oxidoreductase Inhibitor	NA	Aldo/Keto Reductase Family	0.909
4X0M	305	2.7.11.30	NA	Transferase	NA	Protein Kinase Domain	0.914
4X2F	305	2.7.11.30	NA	Transferase/Transferase Inhibitor	NA	Protein Kinase Domain	0.915

PDB ID	Residue count	EC number	SCOPE fold	Classification	CATH description	Protein Family Annotation	Mean persistent similary
4X2G	305	2.7.11.30	NA	Transferase/Transferase Inhibitor	NA	Protein Kinase Domain	0.909
4X2J	305	2.7.11.30	NA	Transferase	NA	Protein Kinase Domain	0.916
4X2K	305	2.7.11.30	NA	Transferase	NA	Protein Kinase Domain	0.916
4X2N	305	2.7.11.30	NA	Transferase	NA	Protein Kinase Domain	0.920
4XZI	316	1.1.1.21	NA	Oxidoreductase	NA	Aldo/Keto Reductase Family	0.905
4YS1	316	1.1.1.21	NA	Oxidoreductase	NA	Aldo/Keto Reductase Family	0.908
4YU1	316	1.1.1.21	NA	Oxidoreductase	NA	Aldo/Keto Reductase Family	0.924
4ZRT	309	3.1.3.48	NA	Hydrolase	NA	Protein-Tyrosine Phosphatase	0.906
4ZZN	350	2.7.11.24	NA	Transferase	NA	Protein Kinase Domain	0.906
5E8Z	307	2.7.11.30	NA	Transferase/Transferase Inhibitor	NA	Protein Kinase Domain	0.908
5GDS	321	3.4.21.5	NA	Hydrolase/Hydrolase Inhibitor	Trypsin-Like Serine Proteases	Trypsin	0.909
5GIM	325	3.4.21.5	NA	Hydrolase/Hydrolase Inhibitor	NA	Thrombin Light Chain	0.905
5JFD	307	3.4.21.5	NA	Hydrolase	NA	Trypsin	0.903
5KAA	289	3.1.3.48	NA	Hydrolase	NA	Protein-Tyrosine Phosphatase	0.909
5LUC	3224	2.6.1.51	NA	Transferase	NA	Aminotransferase Class-V	0.918
5NHH	381	2.7.11.24	NA	Transferase	NA	Protein Kinase Domain	0.901
5QIM	306	2.7.11.30	NA	Transferase/Transferase Inhibitor	NA	Protein Kinase Domain	0.908
5TC6	297	2.4.2.28	NA	Transferase/Transferase Inhibitor	NA	Phosphorylase Superfamily	0.900
5TC8	297	2.4.2.28	NA	Transferase/Transferase Inhibitor	NA	Phosphorylase Superfamily	0.904
5TE0	347	2.7.11.1	NA	Transferase/Transferase Inhibitor	NA	Protein Kinase Domain	0.904
5USQ	299	2.7.11.30	NA	Transferase/Transferase Inhibitor	NA	Protein Kinase Domain	0.910
5Y7N	316	1.1.1.-	NA	Oxidoreductase	NA	Aldo/Keto Reductase Family	0.912
6B8T	321	3.1.3.48	NA	Signaling Protein	NA	Protein-Tyrosine Phosphatase	0.903
6B8X	321	3.1.3.48	NA	Signaling Protein	NA	Protein-Tyrosine Phosphatase	0.902
6B8Y	307	2.7.11.30	NA	Transferase/Transferase Inhibitor	NA	Protein Kinase Domain	0.907
6F7R	316	1.1.1.21	NA	Oxidoreductase	NA	Aldo/Keto Reductase Family	0.900
6F81	316	1.1.1.21	NA	Oxidoreductase	NA	Aldo/Keto Reductase Family	0.904
6F82	316	1.1.1.21	NA	Oxidoreductase	NA	Aldo/Keto Reductase Family	0.913
6F84	316	1.1.1.21	NA	Oxidoreductase	NA	Aldo/Keto Reductase Family	0.916
6F8O	317	1.1.1.21	NA	Oxidoreductase	NA	Aldo/Keto Reductase Family	0.905
6G6J	354	NA	NA	Apoptosis	NA	Helix-Loop-Helix Dna-Binding Domain	0.905
6G8X	368	2.7.11.24	NA	Signaling Protein	NA	Protein Kinase Domain	0.906
6G91	368	2.7.11.24	NA	Signaling Protein	NA	Protein Kinase Domain	0.911
6G9A	368	2.7.11.24	NA	Signaling Protein	NA	Protein Kinase Domain	0.920
6G9K	368	2.7.11.24	NA	Signaling Protein	NA	Protein Kinase Domain	0.901
6G9M	368	2.7.11.24	NA	Signaling Protein	NA	Protein Kinase Domain	0.910
6NBS	367	2.7.11.24	NA	Transferase/Transferase Inhibitor	NA	Protein Kinase Domain	0.908
6OMY	297	3.1.3.48	NA	Hydrolase	NA	Protein-Tyrosine Phosphatase	0.902
6OPG	354	2.7.11.24	NA	Transferase	NA	Protein Kinase Domain	0.906
6OPI	354	2.7.11.24	NA	Transferase	NA	Protein Kinase Domain	0.907
6PFW	297	3.1.3.48	NA	Hydrolase	NA	Protein-Tyrosine Phosphatase	0.909
6PG0	297	3.1.3.48	NA	Hydrolase	NA	Protein-Tyrosine Phosphatase	0.901
6PHA	297	3.1.3.48	NA	Hydrolase	NA	Protein-Tyrosine Phosphatase	0.904
6PHS	297	3.1.3.48	NA	Hydrolase	NA	Protein-Tyrosine Phosphatase	0.908
6Q7S	368	2.7.11.24	NA	Signaling Protein	NA	Protein Kinase Domain	0.905
6Q7T	368	2.7.11.24	NA	Signaling Protein	NA	Protein Kinase Domain	0.911
6QA1	368	2.7.11.24	NA	Signaling Protein	NA	Protein Kinase Domain	0.921
6QA4	368	2.7.11.24	NA	Signaling Protein	NA	Protein Kinase Domain	0.909
6QAL	368	2.7.11.24	NA	Signaling Protein	NA	Protein Kinase Domain	0.924
6QAW	368	2.7.11.24	NA	Signaling Protein	NA	Protein Kinase Domain	0.902
6SLG	386	2.7.11.24	NA	Transferase	NA	Protein Kinase Domain	0.904

PDB ID	Residue count	EC number	SCOPE fold	Classification	CATH description	Protein Family Annotation	Mean persistent similary
1NP0	978	NA	Tim Beta/Alpha-Barrel	Hydrolase	Chitobiase. Domain 2	NA	0.915
1OGS	994	3.2.1.45	Glycosyl Hydrolase Domain	Hydrolase	Glycosidases	Glycosyl Hydrolase Family 30 Beta Sandwich Domain	0.907
1OGU	1124	2.7.1.37	Protein Kinase-Like (Pk-Like)	Transferase	Transferase(Phosphotransferase) Domain 1	Protein Kinase Domain	0.924
1OI9	1124	2.7.1.37	Protein Kinase-Like (Pk-Like)	Kinase	Transferase(Phosphotransferase) Domain 1	Protein Kinase Domain	0.926
1OIU	1124	2.7.1.37	Protein Kinase-Like (Pk-Like)	Kinase	Phosphorylase Kinase; Domain 1	Protein Kinase Domain	0.930
1OIY	1124	2.7.1.37	Protein Kinase-Like (Pk-Like)	Kinase	Transferase(Phosphotransferase) Domain 1	Protein Kinase Domain	0.926
1OJ9	1040	1.4.3.4	Fad/Nad(P)-Binding Domain	Oxidoreductase	Guanine Nucleotide Dissociation Inhibitor. Domain 1	Flavin Containing Amine Oxidoreductase	0.917
1OJC	1040	1.4.3.4	Fad/Nad(P)-Binding Domain	Oxidoreductase	Guanine Nucleotide Dissociation Inhibitor. Domain 1	Flavin Containing Amine Oxidoreductase	0.914
1OKV	1128	2.7.1.37	Protein Kinase-Like (Pk-Like)	Transferase/Transferase Inhibitor	Phosphorylase Kinase; Domain 1	Protein Kinase Domain	0.937
1OS2	990	3.4.24.65	Zincin-Like	Hydrolase	Collagenase (Catalytic Domain)	Matrixin	0.915
1OS9	990	3.4.24.65	Zincin-Like	Hydrolase	Collagenase (Catalytic Domain)	Matrixin	0.913
1OZF	1132	4.1.3.18	Dhs-Like Nad/Fad-Binding Domain	Lyase	Tpp-Binding Domain	Thiamine Pyrophosphate Enzyme. N-Terminal Tpp Binding Domain	0.910
1OZG	1132	4.1.3.18	Dhs-Like Nad/Fad-Binding Domain	Lyase	Tpp-Binding Domain	Thiamine Pyrophosphate Enzyme. N-Terminal Tpp Binding Domain	0.917
1P5E	1114	2.7.1.-	Protein Kinase-Like (Pk-Like)	Cell Cycle	Transferase(Phosphotransferase) Domain 1	Protein Kinase Domain	0.931
1PKD	1110	2.7.1.-	Protein Kinase-Like (Pk-Like)	Transferase/Cell Cycle	Transferase(Phosphotransferase) Domain 1	Protein Kinase Domain	0.931
1QMZ	1130	2.7.1.-	Protein Kinase-Like (Pk-Like)	Cell Cycle	Transferase(Phosphotransferase) Domain 1	Protein Kinase Domain	0.920
1QR6	1168	1.1.1.39	Nad(P)-Binding Rossmann-Fold Domains	Oxidoreductase	Aminoacid Dehydrogenase-Like. N-Terminal Domain. Chain A	Malic Enzyme. Nad Binding Domain	0.906
1S2Q	1040	1.4.3.4	Fad/Nad(P)-Binding Domain	Oxidoreductase	Guanine Nucleotide Dissociation Inhibitor. Domain 1	Flavin Containing Amine Oxidoreductase	0.912
1S3B	1040	1.4.3.4	Fad/Nad(P)-Binding Domain	Oxidoreductase	Guanine Nucleotide Dissociation Inhibitor. Domain 1	Flavin Containing Amine Oxidoreductase	0.901
1S3E	1040	1.4.3.4	Fad/Nad(P)-Binding Domain	Oxidoreductase	Guanine Nucleotide Dissociation Inhibitor. Domain 1	Flavin Containing Amine Oxidoreductase	0.916
1URC	1128	2.7.1.37	Protein Kinase-Like (Pk-Like)	Transferase/Inhibitor	Phosphorylase Kinase; Domain 1	Protein Kinase Domain	0.916
1VYW	1148	2.7.1.37	Protein Kinase-Like (Pk-Like)	Transferase	Phosphorylase Kinase; Domain 1	Protein Kinase Domain	0.935
1X6V	1260	2.7.7.4	Pua Domain-Like	Transferase	P-Loop Containing Nucleotide Triphosphate Hydrolases	Adenylylsulphate Kinase	0.910
1XJQ	1260	2.7.7.4	Pua Domain-Like	Transferase	Hups	Atp-Sulfurylase	0.901
1XMN	1180	3.4.21.5	Trypsin-Like Serine Proteases	Hydrolase/Hydrolase Inhibitor	NA	Thrombin Light Chain	0.916
1XNJ	1260	2.7.7.4	Pua Domain-Like	Transferase	P-Loop Containing Nucleotide Triphosphate Hydrolases	Adenylylsulphate Kinase	0.902
1Y7V	994	3.2.1.45	Glycosyl Hydrolase Domain	Hydrolase	Glycosidases	Glycosyl Hydrolase Family 30 Tim-Barrel Domain	0.902
1YXU	1172	2.7.1.37	NA	Transferase	Phosphorylase Kinase; Domain 1	Protein Kinase Domain	0.946
2B5T	1048	NA	NA	Blood Clotting	Epsilon-Thrombin. Subunit L	Thrombin Light Chain	0.907
2BK3	1040	1.4.3.4	Fad/Nad(P)-Binding Domain	Oxidoreductase	Guanine Nucleotide Dissociation Inhibitor. Domain 1	Flavin Containing Amine Oxidoreductase	0.916
2BK4	1040	1.4.3.4	Fad/Nad(P)-Binding Domain	Oxidoreductase	Guanine Nucleotide Dissociation Inhibitor. Domain 1	Flavin Containing Amine Oxidoreductase	0.913
2BKZ	1148	2.7.1.37	Protein Kinase-Like (Pk-Like)	Transferase	Phosphorylase Kinase; Domain 1	Protein Kinase Domain	0.931
2BPM	1148	2.7.1.37	Protein Kinase-Like (Pk-Like)	Transferase	Phosphorylase Kinase; Domain 1	Protein Kinase Domain	0.933
2BYB	1040	1.4.3.4	Fad/Nad(P)-Binding Domain	Oxidoreductase	Guanine Nucleotide Dissociation Inhibitor. Domain 1	Flavin Containing Amine Oxidoreductase	0.913
2C4G	1148	2.7.1.37	Protein Kinase-Like (Pk-Like)	Transferase	Transferase(Phosphotransferase) Domain 1	Protein Kinase Domain	0.914
2C5N	1114	2.7.1.37	Protein Kinase-Like (Pk-Like)	Transferase	Phosphorylase Kinase; Domain 1	Protein Kinase Domain	0.923
2C5O	1116	2.7.1.37	Protein Kinase-Like (Pk-Like)	Transferase	Transferase(Phosphotransferase) Domain 1	Protein Kinase Domain	0.922
2C5V	1132	2.7.1.37	Protein Kinase-Like (Pk-Like)	Cell Cycle	Transferase(Phosphotransferase) Domain 1	Protein Kinase Domain	0.901
2C5X	1114	2.7.1.37	Protein Kinase-Like (Pk-Like)	Transferase/Inhibitor	Phosphorylase Kinase; Domain 1	Protein Kinase Domain	0.920
2C64	1040	1.4.3.4	Fad/Nad(P)-Binding Domain	Oxidoreductase	Guanine Nucleotide Dissociation Inhibitor. Domain 1	Flavin Containing Amine Oxidoreductase	0.918
2C66	1040	1.4.3.4	Fad/Nad(P)-Binding Domain	Oxidoreductase	Guanine Nucleotide Dissociation Inhibitor. Domain 1	Flavin Containing Amine Oxidoreductase	0.901
2C67	1040	1.4.3.4	Fad/Nad(P)-Binding Domain	Oxidoreductase	Guanine Nucleotide Dissociation Inhibitor. Domain 1	Flavin Containing Amine Oxidoreductase	0.913
2C6F	1224	3.4.15.1	NA	Hydrolase	NA	Angiotensin-Converting Enzyme	0.909
2C6N	1224	3.4.15.1	NA	Hydrolase/Hydrolase Inhibitor	NA	Angiotensin-Converting Enzyme	0.908
2C6T	1112	2.7.1.37	Protein Kinase-Like (Pk-Like)	Cell Cycle	Transferase(Phosphotransferase) Domain 1	Protein Kinase Domain	0.905
2C70	1040	1.4.3.4	Fad/Nad(P)-Binding Domain	Oxidoreductase	Guanine Nucleotide Dissociation Inhibitor. Domain 1	Flavin Containing Amine Oxidoreductase	0.915
2C73	1040	1.4.3.4	Fad/Nad(P)-Binding Domain	Oxidoreductase	Guanine Nucleotide Dissociation Inhibitor. Domain 1	Flavin Containing Amine Oxidoreductase	0.907
2C75	1040	1.4.3.4	Fad/Nad(P)-Binding Domain	Oxidoreductase	Guanine Nucleotide Dissociation Inhibitor. Domain 1	Flavin Containing Amine Oxidoreductase	0.901
2C76	1040	1.4.3.4	Fad/Nad(P)-Binding Domain	Oxidoreductase	Guanine Nucleotide Dissociation Inhibitor. Domain 1	Flavin Containing Amine Oxidoreductase	0.919
2CCH	1142	2.7.1.-	Protein Kinase-Like (Pk-Like)	Cell Cycle	Phosphorylase Kinase; Domain 1	Protein Kinase Domain	0.926
2CCI	1174	2.7.11.22	Protein Kinase-Like (Pk-Like)	Cell Cycle	Transferase(Phosphotransferase) Domain 1	Protein Kinase Domain	0.912
2CJM	1112	2.7.1.37	Protein Kinase-Like (Pk-Like)	Cell Cycle	Phosphorylase Kinase; Domain 1	Protein Kinase Domain	0.926
2F61	994	3.2.1.45	Glycosyl Hydrolase Domain	Hydrolase	Glycosidases	Glycosyl Hydrolase Family 30 Beta Sandwich Domain	0.908
2G1T	1200	2.7.1.112	NA	Transferase	Phosphorylase Kinase; Domain 1	Protein Tyrosine Kinase	0.902
2G9X	1122	2.7.1.37	Protein Kinase-Like (Pk-Like)	Transferase/Cell Cycle	Transferase(Phosphotransferase) Domain 1	Protein Kinase Domain	0.903
2I40	1116	2.7.11.22	Protein Kinase-Like (Pk-Like)	Transferase/Cell Cycle	Phosphorylase Kinase; Domain 1	Protein Kinase Domain	0.940
2IW6	1124	2.7.1.37	Protein Kinase-Like (Pk-Like)	Cell Cycle	Phosphorylase Kinase; Domain 1	Protein Kinase Domain	0.931
2IW8	1124	2.7.1.37	Protein Kinase-Like (Pk-Like)	Cell Cycle	Transferase(Phosphotransferase) Domain 1	Protein Kinase Domain	0.922
2IW9	1124	2.7.1.37	Protein Kinase-Like (Pk-Like)	Cell Cycle	Phosphorylase Kinase; Domain 1	Protein Kinase Domain	0.918
2J25	994	3.2.1.45	Glycosyl Hydrolase Domain	Hydrolase	Golgi Alpha-Mannosidase Ii	Glycosyl Hydrolase Family 30 Tim-Barrel Domain	0.908
2PM8	1148	3.1.1.8	Alpha/Beta-Hydrolases	Hydrolase	NA	Carboxylesterase Family	0.927
2PVY	1296	2.7.10.1	NA	Transferase	Transferase(Phosphotransferase) Domain 1	Protein Tyrosine Kinase	0.902
2RD0	1375	2.7.1.153	NA	Transferase/Oncoprotein	Phosphatidylinositol 3-Kinase Catalytic Subunit; Chain A. Domain 5	Pi3-Kinase Family. P85-Binding Domain	0.909
2UUE	1124	2.7.11.1	Protein Kinase-Like (Pk-Like)	Transferase/Inhibitor	Phosphorylase Kinase; Domain 1	Protein Kinase Domain	0.924
2UZB	1112	2.7.1.37	NA	Transferase	Phosphorylase Kinase; Domain 1	Protein Kinase Domain	0.911
2UZD	1112	2.7.11.22	NA	Transferase	Phosphorylase Kinase; Domain 1	Protein Kinase Domain	0.921
2UZE	1112	2.7.11.22	NA	Transferase	Transferase(Phosphotransferase) Domain 1	Protein Kinase Domain	0.922
2UZL	1112	2.7.11.22	NA	Transferase	Phosphorylase Kinase; Domain 1	Protein Kinase Domain	0.931
2V22	1114	2.7.1.37	NA	Transferase	Phosphorylase Kinase; Domain 1	Protein Kinase Domain	0.932
2V3D	1010	3.2.1.45	Glycosyl Hydrolase Domain	Hydrolase	Golgi Alpha-Mannosidase Ii	Glycosyl Hydrolase Family 30 Beta Sandwich Domain	0.917
2V3E	1010	3.2.1.45	Glycosyl Hydrolase Domain	Hydrolase	Glycosidases	Glycosyl Hydrolase Family 30 Tim-Barrel Domain	0.907
2V3F	1010	3.2.1.45	Glycosyl Hydrolase Domain	Hydrolase	Glycosidases	Glycosyl Hydrolase Family 30 Beta Sandwich Domain	0.921
2V55	1212	2.7.11.1	NA	Transferase	Transferase(Phosphotransferase) Domain 1	Protein Kinase Domain	0.902
2V5Z	1040	1.4.3.4	Fad/Nad(P)-Binding Domain	Oxidoreductase	Guanine Nucleotide Dissociation Inhibitor. Domain 1	Flavin Containing Amine Oxidoreductase	0.915
2V60	1040	1.4.3.4	Fad/Nad(P)-Binding Domain	Oxidoreductase	Guanine Nucleotide Dissociation Inhibitor. Domain 1	Flavin Containing Amine Oxidoreductase	0.914
2V61	1040	1.4.3.4	Fad/Nad(P)-Binding Domain	Oxidoreductase	Guanine Nucleotide Dissociation Inhibitor. Domain 1	Flavin Containing Amine Oxidoreductase	0.916
2VRL	1040	1.4.3.4	Fad/Nad(P)-Binding Domain	Oxidoreductase	Guanine Nucleotide Dissociation Inhibitor. Domain 1	Flavin Containing Amine Oxidoreductase	0.915
2VRM	1040	1.4.3.4	Fad/Nad(P)-Binding Domain	Oxidoreductase	Guanine Nucleotide Dissociation Inhibitor. Domain 1	Flavin Containing Amine Oxidoreductase	0.913
2VT0	1010	3.2.1.45	Glycosyl Hydrolase Domain	Hydrolase	Glycosidases	Glycosyl Hydrolase Family 30 Beta Sandwich Domain	0.919
2VZ2	1040	1.4.3.4	NA	Oxidoreductase	Guanine Nucleotide Dissociation Inhibitor. Domain 1	Flavin Containing Amine Oxidoreductase	0.909
2WCG	1010	3.2.1.45	NA	Hydrolase	Glycosidases	Glycosyl Hydrolase Family 30 Beta Sandwich Domain	0.928
2WEV	1128	2.7.1.37	NA	Transferase	Transferase(Phosphotransferase) Domain 1	Protein Kinase Domain	0.902
2WFY	1128	2.7.1.37	NA	Transferase	Transferase(Phosphotransferase) Domain 1	Protein Kinase Domain	0.912
2WIH	1148	2.7.1.37	NA	Transferase	Phosphorylase Kinase; Domain 1	Protein Kinase Domain	0.938
2WIL	1058	3.1.1.8	NA	Hydrolase	NA	Carboxylesterase Family	0.925
2WIP	1148	2.7.1.37	NA	Transferase	Phosphorylase Kinase; Domain 1	Protein Kinase Domain	0.931
2WKL	994	3.2.1.45	NA	Hydrolase	Golgi Alpha-Mannosidase Ii	Glycosyl Hydrolase Family 30 Tim-Barrel Domain	0.926
2WMA	1129	2.7.11.22	NA	Cell Cycle	Phosphorylase Kinase; Domain 1	Protein Kinase Domain	0.921
2WMB	1129	2.7.11.22	NA	Cell Cycle	Phosphorylase Kinase; Domain 1	Protein Kinase Domain	0.921
2WPA	1148	2.7.1.37	NA	Cell Cycle	Transferase(Phosphotransferase) Domain 1	Protein Kinase Domain	0.930
2WXV	1148	2.7.1.37	NA	Transferase	Phosphorylase Kinase; Domain 1	Protein Kinase Domain	0.927
2XCG	1040	1.4.3.4	NA	Oxidoreductase	Guanine Nucleotide Dissociation Inhibitor. Domain 1	Flavin Containing Amine Oxidoreductase	0.912

PDB ID	Residue count	EC number	SCOPE fold	Classification	CATH description	Protein Family Annotation	Mean persistent similary
2XFN	1040	1.4.3.4	NA	Oxidoreductase	Guanine Nucleotide Dissociation Inhibitor. Domain 1	Flavin Containing Amine Oxidoreductase	0.916
2XFO	1040	1.4.3.4	NA	Oxidoreductase	Guanine Nucleotide Dissociation Inhibitor. Domain 1	Flavin Containing Amine Oxidoreductase	0.918
2XFP	1040	1.4.3.4	NA	Oxidoreductase	Guanine Nucleotide Dissociation Inhibitor. Domain 1	Flavin Containing Amine Oxidoreductase	0.907
2XFQ	1040	1.4.3.4	NA	Oxidoreductase	Guanine Nucleotide Dissociation Inhibitor. Domain 1	Flavin Containing Amine Oxidoreductase	0.914
2XFU	1038	1.4.3.4	NA	Oxidoreductase	Guanine Nucleotide Dissociation Inhibitor. Domain 1	Flavin Containing Amine Oxidoreductase	0.913
2XWD	1010	3.2.1.45	NA	Hydrolase	Glycosidases	Glycosyl Hydrolase Family 30 Tim-Barrel Domain	0.930
2XWE	1010	3.2.1.45	NA	Hydrolase	Glycosidases	Glycosyl Hydrolase Family 30 Beta Sandwich Domain	0.925
2ZC4	1356	NA	NA	Biosynthetic Protein	NA	Penicillin-Binding Protein Dimerisation Domain	0.914
3BHT	1124	2.7.11.22	Protein Kinase-Like (Pk-Like)	Transferase	Phosphorylase Kinase; Domain 1	Protein Kinase Domain	0.921
3BHU	1124	2.7.11.22	NA	Transferase	Transferase(Phosphotransferase) Domain 1	Protein Kinase Domain	0.923
3BHV	1124	2.7.11.22	NA	Transferase	Phosphorylase Kinase; Domain 1	Protein Kinase Domain	0.922
3C4E	1092	2.7.11.1	NA	Transferase	Transferase(Phosphotransferase) Domain 1	Protein Kinase Domain	0.937
3CDG	1250	NA	Immunoglobulin-Like Beta-Sandwich	Immune System	Murine Class I Major Histocompatibility Complex. H2-Db. Subunit A. Domain 1	Immunoglobulin C1-Set Domain	0.908
3DAK	1160	2.7.11.1	NA	Transferase	Phosphorylase Kinase; Domain 1	Protein Kinase Domain	0.924
3DDQ	1136	2.7.11.22	NA	Transferase/Cell Cycle	Transferase(Phosphotransferase) Domain 1	Protein Kinase Domain	0.928
3DG8	1216	1.5.1.3	NA	Oxidoreductase. Transferase	Dihydrofolate Reductase. Subunit A	Dihydrofolate Reductase	0.902
3DGA	1216	1.5.1.3	NA	Oxidoreductase. Transferase	Dihydrofolate Reductase. Subunit A	Dihydrofolate Reductase	0.907
3DOG	1126	2.7.11.22	NA	Transferase. Cell Cycle	Phosphorylase Kinase; Domain 1	Protein Kinase Domain	0.932
3DS6	1464	2.7.11.24	Protein Kinase-Like (Pk-Like)	Transferase	Transferase(Phosphotransferase) Domain 1	Protein Kinase Domain	0.923
3EID	1116	2.7.11.22	NA	Transferase/Cell Cycle	Phosphorylase Kinase; Domain 1	Protein Kinase Domain	0.931
3EJ1	1116	2.7.11.22	NA	Transferase/Cell Cycle	Phosphorylase Kinase; Domain 1	Protein Kinase Domain	0.929
3EJL	1252	2.1.1.45	NA	Transferase	Thymidylate Synthase. Chain A	Thymidylate Synthase	0.911
3ENM	1264	2.7.12.2	NA	Transferase	NA	Protein Kinase Domain	0.903
3F3Y	1140	2.8.2.14	NA	Transferase	P-Loop Containing Nucleotide Triphosphate Hydrolases	Sulfotransferase Domain	0.904
3F5X	1108	2.7.11.22	NA	Transferase/Cell Cycle	Phosphorylase Kinase; Domain 1	Protein Kinase Domain	0.932
3F9P	1162	1.11.1.7	NA	Oxidoreductase	NA	Animal Haem Peroxidase	0.905
3FGA	1354	NA	NA	Hydrolase/Hydrolase Inhibitor	Leucine-Rich Repeat Variant	Heat Repeats	0.903
3GG5	1252	2.1.1.45	NA	Transferase	Thymidylate Synthase. Chain A	Thymidylate Synthase	0.910
3IYP	1241	NA	NA	Virus	NA	Picornavirus Capsid Protein	0.903
3KE0	994	3.2.1.45	NA	Hydrolase	Glycosidases	Glycosyl Hydrolase Family 30 Tim-Barrel Domain	0.911
3KEH	994	3.2.1.45	NA	Hydrolase	Glycosidases	Glycosyl Hydrolase Family 30 Beta Sandwich Domain	0.908
3L4T	875	3.2.1.20	NA	Hydrolase	NA	N-Terminal Barrel Of Ntmgam And Ctmgam. Maltase-Glucoamylase	0.905
3L4V	875	3.2.1.20	NA	Hydrolase	NA	N-Terminal Barrel Of Ntmgam And Ctmgam. Maltase-Glucoamylase	0.906
3L4W	875	3.2.1.20	NA	Hydrolase	NA	N-Terminal Barrel Of Ntmgam And Ctmgam. Maltase-Glucoamylase	0.912
3L4X	875	3.2.1.20	NA	Hydrolase	NA	N-Terminal Barrel Of Ntmgam And Ctmgam. Maltase-Glucoamylase	0.901
3L4Z	875	3.2.1.20	NA	Hydrolase	NA	N-Terminal Barrel Of Ntmgam And Ctmgam. Maltase-Glucoamylase	0.913
3LII	1080	3.1.1.7	NA	Hydrolase	NA	Carboxylesterase Family	0.907
3LMY	1112	3.2.1.52	NA	Hydrolase	Glycosidases	Beta-Acetyl Hexosaminidase Like	0.913
3MY5	1124	2.7.11.22	NA	Transferase/Protein Binding/Inhibitor	Phosphorylase Kinase; Domain 1	Protein Kinase Domain	0.926
3O9M	1148	3.1.1.8	NA	Hydrolase	NA	Carboxylesterase Family	0.920
3ORI	1244	NA	NA	Transferase	Phosphorylase Kinase; Domain 1	Protein Kinase Domain	0.904
3P1M	1056	NA	NA	Electron Transport	NA	2fe-2s Iron-Sulfur Cluster Binding Domain	0.913
3P70	1464	3.4.21.5	NA	Hydrolase	NA	Thrombin Light Chain	0.920
3PO7	1040	1.4.3.4	NA	Oxidoreductase/Oxidoreductase Inhibitor	Guanine Nucleotide Dissociation Inhibitor. Domain 1	Flavin Containing Amine Oxidoreductase	0.914
3QHR	1158	2.7.11.22	NA	Transferase/Protein Binding	Phosphorylase Kinase; Domain 1	Protein Kinase Domain	0.901
3TNW	1124	2.7.11.22	NA	Transferase/Transferase Inhibitor	Transferase(Phosphotransferase) Domain 1	Protein Kinase Domain	0.922
3UW6	1170	5.1.1.1	NA	Isomerase	Lyase. Ornithine Decarboxylase; Chain A. Domain 1	Alanine Racemase. N-Terminal Domain	0.906
3WJA	1160	1.1.1.40	NA	Oxidoreductase	NA	Malic Enzyme. Nad Binding Domain	0.920
3ZC6	1152	2.7.10.2	NA	Transferase	NA	Protein Tyrosine Kinase	0.925
3ZEP	1152	2.7.10.2	NA	Transferase	NA	Protein Tyrosine Kinase	0.916
3ZHP	1268	NA	NA	Cell Cycle	Leucine-Rich Repeat Variant	Mo25-Like	0.913
3ZLK	1212	2.7.7.74	NA	Transferase	Spore Coat Polysaccharide Biosynthesis Protein Spsa; Chain A	Nucleotidyl Transferase	0.912
3ZS0	1150	1.11.2.2	NA	Oxidoreductase	NA	Animal Haem Peroxidase	0.917
3ZS1	1162	1.11.2.2	NA	Oxidoreductase	NA	Animal Haem Peroxidase	0.913
4A55	1375	2.7.1.153	NA	Transferase	NA	Pi3-Kinase Family. Ras-Binding Domain	0.911
4A69	940	3.5.1.98	NA	Transcription	NA	Histone Deacetylase Domain	0.914
4A79	1040	1.4.3.4	NA	Oxidoreductase	Guanine Nucleotide Dissociation Inhibitor. Domain 1	Flavin Containing Amine Oxidoreductase	0.914
4A7A	1040	1.4.3.4	NA	Oxidoreductase	Guanine Nucleotide Dissociation Inhibitor. Domain 1	Flavin Containing Amine Oxidoreductase	0.917
4AQD	1062	3.1.1.8	NA	Hydrolase	NA	Carboxylesterase Family	0.911
4ARW	1208	2.7.7.74	NA	Transferase	Spore Coat Polysaccharide Biosynthesis Protein Spsa; Chain A	Nucleotidyl Transferase	0.908
4ASJ	1172	2.7.7.74	NA	Transferase	Spore Coat Polysaccharide Biosynthesis Protein Spsa; Chain A	Nucleotidyl Transferase	0.907
4B2W	1212	2.7.7.74	NA	Transferase	NA	Nucleotidyl Transferase	0.901
4B42	1212	2.7.7.74	NA	Transferase	Spore Coat Polysaccharide Biosynthesis Protein Spsa; Chain A	Nucleotidyl Transferase	0.903
4B4G	1212	2.7.7.74	NA	Transferase	Spore Coat Polysaccharide Biosynthesis Protein Spsa; Chain A	Nucleotidyl Transferase	0.911
4B4M	1212	2.7.7.74	NA	Transferase	Spore Coat Polysaccharide Biosynthesis Protein Spsa; Chain A	Nucleotidyl Transferase	0.908
4BBE	1192	2.7.10.2	NA	Transferase	Transferase(Phosphotransferase) Domain 1	Protein Tyrosine Kinase	0.910
4BBF	1192	2.7.10.2	NA	Transferase	Transferase(Phosphotransferase) Domain 1	Protein Tyrosine Kinase	0.905
4BCM	1126	2.7.11.22	NA	Transferase/Cell Cycle	Transferase(Phosphotransferase) Domain 1	Protein Kinase Domain	0.923
4BCN	1124	2.7.11.22	NA	Transferase/Cell Cycle	Transferase(Phosphotransferase) Domain 1	Protein Kinase Domain	0.929
4BCO	1124	2.7.11.22	NA	Transferase/Cell Cycle	Transferase(Phosphotransferase) Domain 1	Protein Kinase Domain	0.929
4BCP	1124	2.7.11.22	NA	Transferase/Cell Cycle	NA	Protein Kinase Domain	0.923
4BXK	1258	3.2.1.-	NA	Hydrolase	NA	Angiotensin-Converting Enzyme	0.908
4C1M	1150	1.11.2.2	NA	Oxidoreductase	NA	Animal Haem Peroxidase	0.908
4CFM	1122	2.7.11.22	NA	Cell Cycle	NA	Protein Kinase Domain	0.925
4CFN	1120	2.7.11.22	NA	Cell Cycle	NA	Protein Kinase Domain	0.934
4CFU	1130	2.7.11.22	NA	Transferase	NA	Protein Kinase Domain	0.930
4CFV	1130	2.7.11.22	NA	Transferase	NA	Protein Kinase Domain	0.932
4CFW	1122	2.7.11.22	NA	Cell Cycle	NA	Protein Kinase Domain	0.915
4CH8	1236	3.4.21.5	NA	Hydrolase/Peptide	NA	Thrombin Light Chain	0.906
4COO	1126	4.2.1.22	NA	Lyase	NA	Cbs Domain	0.913
4CRT	1040	1.4.3.4	NA	Oxidoreductase	NA	Flavin Containing Amine Oxidoreductase	0.913
4E4M	1208	2.7.10.2	NA	Transferase/Transferase Inhibitor	Phosphorylase Kinase; Domain 1	Protein Tyrosine Kinase	0.919
4EOI	1114	2.7.11.22	NA	Transferase/Transferase Inhibitor	Phosphorylase Kinase; Domain 1	Protein Kinase Domain	0.926
4EOJ	1120	2.7.11.22	NA	Transferase. Cell Cycle	Phosphorylase Kinase; Domain 1	Protein Kinase Domain	0.918
4EOK	1116	2.7.11.22	NA	Transferase/Transferase Inhibitor	Phosphorylase Kinase; Domain 1	Protein Kinase Domain	0.922
4EOL	1116	2.7.11.22	NA	Transferase/Transferase Inhibitor	Phosphorylase Kinase; Domain 1	Protein Kinase Domain	0.926
4EOM	1118	2.7.11.22	NA	Transferase. Cell Cycle	Phosphorylase Kinase; Domain 1	Protein Kinase Domain	0.921
4EON	1116	2.7.11.22	NA	Transferase/Transferase Inhibitor	Phosphorylase Kinase; Domain 1	Protein Kinase Domain	0.929
4EOO	1114	2.7.11.22	NA	Transferase. Cell Cycle	Transferase(Phosphotransferase) Domain 1	Protein Kinase Domain	0.910
4EOP	1116	2.7.11.22	NA	Transferase/Transferase Inhibitor	Transferase(Phosphotransferase) Domain 1	Protein Kinase Domain	0.922
4EOQ	1118	2.7.11.22	NA	Transferase. Cell Cycle	Transferase(Phosphotransferase) Domain 1	Protein Kinase Domain	0.926
4EOR	1112	2.7.11.22	NA	Transferase/Transferase Inhibitor	Transferase(Phosphotransferase) Domain 1	Protein Kinase Domain	0.918
4EOS	1116	2.7.11.22	NA	Transferase/Transferase Inhibitor	Transferase(Phosphotransferase) Domain 1	Protein Kinase Domain	0.915
4EXM	1388	3.2.1.17	NA	Toxin. Hydrolase	NA	Phage Lysozyme	0.908
4EY4	1084	3.1.1.7	NA	Hydrolase	NA	Carboxylesterase Family	0.909
4FX3	1112	2.7.11.22	NA	Transferase/Transferase Inhibitor	NA	Protein Kinase Domain	0.927
4H1I	1272	2.1.1.45	NA	Transferase	NA	Thymidylate Synthase	0.910

PDB ID	Residue count	EC number	SCOPE fold	Classification	CATH description	Protein Family Annotation	Mean persistent similary
4HZS	1364	2.7.10.2	NA	Transferase	NA	Variant Sh3 Domain	0.902
4I3Z	1106	2.7.11.22	NA	Transferase/Cell Cycle	Phosphorylase Kinase; Domain 1	Protein Kinase Domain	0.919
4II5	1112	2.7.11.22	NA	Transferase/Cell Cycle	Phosphorylase Kinase; Domain 1	Protein Kinase Domain	0.925
4ILS	1170	NA	NA	Structural Genomics. Unknown Function	Alanine Racemase	Alanine Racemase. C-Terminal Domain	0.938
4IQR	1484	NA	NA	Transcription/Dna	NA	Zinc Finger. C4 Type (Two Domains)	0.905
4JWU	1056	1.14.15.1	NA	Oxidoreductase/Electron Transport	NA	Cytochrome P450	0.922
4O1X	1300	2.1.1.45	NA	Transferase	NA	Thymidylate Synthase	0.905
4RIW	1342	2.7.10.1	NA	Transferase	NA	Protein Tyrosine Kinase	0.923
4RIX	1342	2.7.10.1	NA	Transferase	NA	Protein Tyrosine Kinase	0.923
4RIY	1342	2.7.10.1	NA	Transferase	NA	Protein Tyrosine Kinase	0.915
4TPK	1204	3.1.1.8	NA	Hydrolase	NA	Carboxylesterase Family	0.915
4UP1	1252	2.1.1.45	NA	Transferase	NA	Thymidylate Synthase	0.905
4XII	1088	3.1.1.8	NA	Hydrolase	NA	Carboxylesterase Family	0.923
4YKN	1383	2.7.1.153	NA	Transferase/Transferase Inhibitor	NA	Phosphatidylinositol 3-Kinase Regulatory Subunit P85 Inter-Sh2 Domain	0.918
4YZ9	1215	2.7.11.1	NA	Transferase/Transferase Inhibitor	NA	Protein Kinase Domain	0.914
4YZD	1215	2.7.11.1	NA	Transferase	NA	Protein Kinase Domain	0.942
4Z16	1264	2.7.10.2	NA	Transferase/Transferase Inhibitor	NA	Protein Tyrosine Kinase	0.915
5CYI	1126	2.7.11.22	NA	Transferase	NA	Protein Kinase Domain	0.919
5D6R	1158	2.2.1.6	NA	Transferase	NA	Thiamine Pyrophosphate Enzyme. N-Terminal Tpp Binding Domain	0.912
5DX6	1158	2.2.1.6	NA	Transferase	NA	Thiamine Pyrophosphate Enzyme. N-Terminal Tpp Binding Domain	0.922
5DYT	1060	3.1.1.8	NA	Hydrolase	NA	Carboxylesterase Family	0.914
5DYW	1060	3.1.1.8	NA	Hydrolase	NA	Carboxylesterase Family	0.909
5DYY	1060	3.1.1.8	NA	Hydrolase	NA	Carboxylesterase Family	0.915
5F19	1104	1.14.99.1	NA	Oxidoreductase/Inhibitor	NA	Egf-Like Domain	0.904
5F1A	1106	1.14.99.1	NA	Oxidoreductase/Inhibitor	NA	Animal Haem Peroxidase	0.913
5FBH	1136	NA	NA	Signaling Protein	NA	Receptor Family Ligand Binding Region	0.909
5FBK	1136	NA	NA	Signaling Protein	NA	Receptor Family Ligand Binding Region	0.922
5FIW	1142	1.11.2.2	NA	Oxidoreductase	NA	Animal Haem Peroxidase	0.920
5FO8	1812	3.4.21.47	NA	Lipid Binding	NA	Mg2 Domain	0.925
5FOQ	1096	3.1.1.7	NA	Hydrolase	NA	Carboxylesterase Family	0.915
5FPQ	1084	3.1.1.7	NA	Hydrolase	NA	Carboxylesterase Family	0.914
5FTS	1212	2.7.7.24	NA	Transferase	NA	Nucleotidyl Transferase	0.905
5FTV	1212	2.7.7.24	NA	Transferase	NA	Nucleotidyl Transferase	0.906
5FU0	1212	2.7.7.24	NA	Transferase	NA	Nucleotidyl Transferase	0.903
5FU8	1212	2.7.7.24	NA	Transferase	NA	Nucleotidyl Transferase	0.904
5FUH	1212	2.7.7.24	NA	Transferase	NA	Nucleotidyl Transferase	0.901
5FYE	1212	2.7.7.24	NA	Transferase	NA	Nucleotidyl Transferase	0.907
5HF6	1084	3.1.1.7	NA	Hydrolase	NA	Carboxylesterase Family	0.923
5HF8	1084	3.1.1.7	NA	Hydrolase	NA	Carboxylesterase Family	0.915
5HF9	1084	3.1.1.7	NA	Hydrolase	NA	Carboxylesterase Family	0.912
5HIE	1208	2.7.11.1	NA	Transferase/Transferase Inhibitor	NA	Protein Tyrosine Kinase	0.929
5IF1	1126	2.7.11.22	NA	Transferase	NA	Protein Kinase Domain	0.924
5IKQ	1102	1.14.99.1	NA	Oxidoreductase	NA	Animal Haem Peroxidase	0.910
5IKR	1102	1.14.99.1	NA	Oxidoreductase	NA	Animal Haem Peroxidase	0.913
5IKT	1102	1.14.99.1	NA	Oxidoreductase	NA	Animal Haem Peroxidase	0.915
5IKV	1102	1.14.99.1	NA	Oxidoreductase	NA	Egf-Like Domain	0.912
5IQ8	1052	3.5.2.6	NA	Hydrolase	NA	Beta-Lactamase Enzyme Family	0.900
5JG8	1172	3.1.4.12	NA	Hydrolase	NA	Calcineurin-Like Phosphoesterase	0.924
5K5E	1058	3.1.1.8	NA	Hydrolase	NA	Carboxylesterase Family	0.927
5K5S	1230	NA	NA	Signaling Protein	NA	Receptor Family Ligand Binding Region	0.919
5KIR	1102	1.14.99.1	NA	Oxidoreductase	NA	Animal Haem Peroxidase	0.907
5LKR	1148	3.1.1.8	NA	Hydrolase	NA	Carboxylesterase Family	0.920
5LMK	1112	2.7.11.22	NA	Transferase	NA	Protein Kinase Domain	0.920
5LVP	1304	2.7.11.1	NA	Transferase	NA	Protein Kinase Domain	0.909
5MRL	1040	1.4.3.4	NA	Oxidoreductase	NA	Flavin Containing Amine Oxidoreductase	0.920
5NEV	1126	2.7.11.22	NA	Transferase	NA	Protein Kinase Domain	0.918
5OY6	1204	2.7.11.30	NA	Signaling Protein	NA	Protein Tyrosine Kinase	0.905
5QQQ	938	2.3.1.37	NA	Transferase	NA	Aminotransferase Class I And Ii	0.911
5QQR	938	2.3.1.37	NA	Transferase	NA	Aminotransferase Class I And Ii	0.909
5QQS	938	2.3.1.37	NA	Transferase	NA	Aminotransferase Class I And Ii	0.907
5QQT	938	2.3.1.37	NA	Transferase	NA	Aminotransferase Class I And Ii	0.910
5QQU	938	2.3.1.37	NA	Transferase	NA	Aminotransferase Class I And Ii	0.907
5QQV	938	2.3.1.37	NA	Transferase	NA	Aminotransferase Class I And Ii	0.903
5QQW	938	2.3.1.37	NA	Transferase	NA	Aminotransferase Class I And Ii	0.909
5QQX	938	2.3.1.37	NA	Transferase	NA	Aminotransferase Class I And Ii	0.908
5QQY	938	2.3.1.37	NA	Transferase	NA	Aminotransferase Class I And Ii	0.911
5QQZ	938	2.3.1.37	NA	Transferase	NA	Aminotransferase Class I And Ii	0.910
5QR0	938	2.3.1.37	NA	Transferase	NA	Aminotransferase Class I And Ii	0.905

PDB ID	Residue count	EC number	SCOPE fold	Classification	CATH description	Protein Family Annotation	Mean persistent similary
5QR1	938	2.3.1.37	NA	Transferase	NA	Aminotransferase Class I And Ii	0.909
5QR2	938	2.3.1.37	NA	Transferase	NA	Aminotransferase Class I And Ii	0.907
5QR3	938	2.3.1.37	NA	Transferase	NA	Aminotransferase Class I And Ii	0.908
5QR4	938	2.3.1.37	NA	Transferase	NA	Aminotransferase Class I And Ii	0.910
5QR5	938	2.3.1.37	NA	Transferase	NA	Aminotransferase Class I And Ii	0.910
5QR6	938	2.3.1.37	NA	Transferase	NA	Aminotransferase Class I And Ii	0.907
5QR7	938	2.3.1.37	NA	Transferase	NA	Aminotransferase Class I And Ii	0.909
5QR8	938	2.3.1.37	NA	Transferase	NA	Aminotransferase Class I And Ii	0.913
5QR9	938	2.3.1.37	NA	Transferase	NA	Aminotransferase Class I And Ii	0.909
5QRA	938	2.3.1.37	NA	Transferase	NA	Aminotransferase Class I And Ii	0.909
5QRB	938	2.3.1.37	NA	Transferase	NA	Aminotransferase Class I And Ii	0.910
5QRC	938	2.3.1.37	NA	Transferase	NA	Aminotransferase Class I And Ii	0.910
5QRD	938	2.3.1.37	NA	Transferase	NA	Aminotransferase Class I And Ii	0.900
5QRE	938	2.3.1.37	NA	Transferase	NA	Aminotransferase Class I And Ii	0.908
5QT3	938	2.3.1.37	NA	Transferase	NA	Aminotransferase Class I And Ii	0.910
5SW8	1375	2.7.1.153	NA	Transferase/Transferase Inhibitor	NA	Pi3-Kinase Family. Ras-Binding Domain	0.905
5SXF	1375	2.7.1.153	NA	Transferase/Transferase Inhibitor	NA	Phosphoinositide 3-Kinase C2	0.912
5VA9	1032	3.2.1.1	NA	Hydrolase/Hydrolase Inhibitor	NA	Alpha Amylase. Catalytic Domain	0.908
5VBN	1344	2.7.7.7	NA	Transferase	NA	Dna Polymerase Alpha/Epsilon Subunit B	0.923
5VK0	1188	2.3.2.27	NA	Ligase/Ligase Inhibitor	NA	Swib/Mdm2 Domain	0.900
5VL7	1142	3.4.21.-	NA	Hydrolase	NA	Peptidase Inhibitor I9	0.907
5WDG	1158	2.2.1.6	NA	Transferase	NA	Thiamine Pyrophosphate Enzyme. N-Terminal Tpp Binding Domain	0.904
5X3V	992	2.1.2.1	NA	Transferase	NA	Serine Hydroxymethyltransferase	0.900
5YT3	1400	NA	NA	Transferase	NA	Protein Kinase Domain	0.916
6B2N	1052	3.5.2.6	NA	Hydrolase	NA	Beta-Lactamase Enzyme Family	0.904
6B5J	1244	2.7.11.1	NA	Transferase/Transferase Inhibitor	NA	Protein Tyrosine Kinase	0.911
6CD6	1160	2.7.11.17	NA	Transferase/Transferase Inhibitor	NA	Protein Kinase Domain	0.923
6CQU	1084	3.1.1.7	NA	Hydrolase	NA	Carboxylesterase Family	0.917
6CQV	1084	3.1.1.7	NA	Hydrolase	NA	Carboxylesterase Family	0.918
6CQW	1084	3.1.1.7	NA	Hydrolase	NA	Carboxylesterase Family	0.911
6CQX	1084	3.1.1.7	NA	Hydrolase	NA	Carboxylesterase Family	0.926
6CQY	1084	3.1.1.7	NA	Hydrolase	NA	Carboxylesterase Family	0.906
6CQZ	1084	3.1.1.7	NA	Hydrolase	NA	Carboxylesterase Family	0.907
6DF6	1120	NA	NA	Nuclear Protein	NA	Ligand-Binding Domain Of Nuclear Hormone Receptor	0.915
6E69	872	3.4.21.37	NA	Hydrolase/Hydrolase Inhibitor	NA	Trypsin	0.904
6EMI	1064	3.1.1.8	NA	Hydrolase	NA	Carboxylesterase Family	0.915
6ESJ	1058	3.1.1.8	NA	Hydrolase	NA	Carboxylesterase Family	0.912
6ESY	1058	3.1.1.8	NA	Hydrolase	NA	Carboxylesterase Family	0.925
6EZ2	1054	3.1.1.8	NA	Hydrolase	NA	Carboxylesterase Family	0.921
6F25	1078	3.1.1.7	NA	Hydrolase	NA	Carboxylesterase Family	0.916
6F7Q	1058	3.1.1.8	NA	Hydrolase	NA	Carboxylesterase Family	0.924
6F9V	1258	3.2.1.-	NA	Hydrolase	NA	Angiotensin-Converting Enzyme	0.910
6FVZ	1040	1.4.3.4	NA	Flavoprotein	NA	Flavin Containing Amine Oxidoreductase	0.917
6FW0	1040	1.4.3.4	NA	Flavoprotein	NA	Flavin Containing Amine Oxidoreductase	0.913
6FWC	1040	1.4.3.4	NA	Flavoprotein	NA	Flavin Containing Amine Oxidoreductase	0.912
6GQ3	1122	6.3.5.4	NA	Biosynthetic Protein	NA	Asparagine Synthase	0.927
6GUB	1140	2.7.11.22	NA	Cell Cycle	NA	Protein Kinase Domain	0.910
6GUC	1140	2.7.11.22	NA	Cell Cycle	NA	Protein Kinase Domain	0.922
6GUE	1140	2.7.11.22	NA	Cell Cycle	NA	Protein Kinase Domain	0.923
6GUF	1140	2.7.11.22	NA	Cell Cycle	NA	Protein Kinase Domain	0.913
6H5X	1258	3.2.1.-	NA	Hydrolase	NA	Angiotensin-Converting Enzyme	0.901
6HRH	938	2.3.1.37	NA	Oxidoreductase	NA	Aminotransferase Class I And Ii	0.911
6HXF	1208	2.7.11.1	NA	Transferase	NA	Protein Kinase Domain	0.943
6ILZ	1380	2.7.11.13	NA	Transferase	NA	Protein Kinase Domain	0.910
6MBW	1144	NA	NA	Transcription Activator	NA	Stat Protein. All-Alpha Domain	0.908
6MOZ	994	3.2.1.45	NA	Hydrolase	NA	Glycosyl Hydrolase Family 30 Tim-Barrel Domain	0.922
6NBL	1054	1.14.15.1	NA	Oxidoreductase	NA	Cytochrome P450	0.907
6O50	1100	3.1.1.7	NA	Hydrolase	NA	Carboxylesterase Family	0.902
6O52	1100	3.1.1.7	NA	Hydrolase	NA	Carboxylesterase Family	0.910
6O5R	1100	3.1.1.7	NA	Hydrolase	NA	Carboxylesterase Family	0.908
6O5S	1100	3.1.1.7	NA	Hydrolase	NA	Carboxylesterase Family	0.910
6P3W	1124	2.7.11.22	NA	Cell Cycle	NA	Protein Kinase Domain	0.901
6Q6K	994	3.2.1.45	NA	Hydrolase	NA	Glycosyl Hydrolase Family 30 Beta Sandwich Domain	0.918
6Q6L	994	3.2.1.45	NA	Hydrolase	NA	Glycosyl Hydrolase Family 30 Tim-Barrel Domain	0.915
6Q6N	994	3.2.1.45	NA	Hydrolase	NA	Glycosyl Hydrolase Family 30 Tim-Barrel Domain	0.914
6QVL	1008	2.1.2.1	NA	Transferase	NA	Serine Hydroxymethyltransferase	0.903
6S1F	1268	2.7.11.1	NA	Signaling Protein	NA	Protein Tyrosine Kinase	0.907
6S8L	1058	NA	NA	Structural Protein	NA	Tubulin/Ftsz Family. Gtpase Domain	0.913
6T6D	1204	2.7.11.30	NA	Signaling Protein	NA	Protein Tyrosine Kinase	0.908

Supplementary Table 3: Proteins targeted by Drugbank FDA-approved medications showing average persistent similarity measures higher than 0.9 with 6M71.

Drug name	Drug ID	DS1 PC	DS2 PC	DS3 PC	Autodock LE	Autodock cluster
**Danazol**	DB01406	NA	NA	NA	-8.31	149
**Bazedoxifene**	DB06401	-0.0215722028	-0.02560255855	-0.01968259763	-8.3	132
**Trilostane**	DB01108	NA	NA	NA	-8.28	150
**Estradiol benzoate**	DB13953	NA	NA	NA	-8.26	79
**Alpelisib**	DB12015	-0.02748757587	-0.03637858041	-0.02711652859	-8.04	136
**Norgestimate**	DB00957	NA	NA	NA	-7.78	82
**Meloxicam**	DB00814	NA	NA	NA	-7.77	111
**Etonogestrel**	DB00294	NA	NA	NA	-7.57	51
**Tibolone**	DB09070	NA	NA	NA	-7.56	128
**Regorafenib**	DB08896	-0.02240332949	-0.03581579405	-0.02348317089	-7.5	47
**Estradiol acetate**	DB13952	NA	NA	NA	-7.45	123
**Lornoxicam**	DB06725	NA	NA	NA	-7.43	122
**Tenoxicam**	DB00469	NA	NA	NA	-7.38	87
**Sorafenib**	DB00398	-0.1139503326	-0.1488412632	-0.0528631983	-7.34	18
**Parecoxib**	DB08439	-0.02956392566	-0.09595335133	-0.04444581116	-7.33	8
**Norethynodrel**	DB09371	NA	NA	NA	-7.32	150
**Acemetacin**	DB13783	-0.04903803014	-0.03113981002	-0.0295586911	-7.32	15
**Piroxicam**	DB00554	NA	NA	NA	-7.31	79
**Pyridoxal phosphate**	DB00114	NA	NA	NA	-7.25	18
**Sulindac**	DB00605	-0.06768358761	-0.1193287796	-0.06631246593	-7.23	32
**Gestrinone**	DB11619	-0.02317537043	-0.0289833741	-0.01718246109	-7.22	81
**Levonorgestrel**	DB00367	-0.08286521718	-0.1387530571	-0.07915695554	-7.21	89
**Zofenopril**	DB13166	-0.02675101496	-0.028950874	-0.02815095671	-7.15	3
**Prasterone**	DB01708	NA	NA	NA	-7.13	129
**Naloxone**	DB01183	-0.05572216872	-0.1159793987	-0.08947993744	-7.08	1
**Raloxifene**	DB00481	-0.1278104657	-0.1700041351	-0.0726603481	-7.05	1
**Phenolphthalein**	DB04824	-0.1299268982	-0.09894230167	-0.03725859228	-6.99	46

Drug name	Drug ID	DS1 PC	DS2 PC	DS3 PC	Autodock LE	Autodock cluster
**Carprofen**	DB00821	-0.07383721985	-0.08740776905	-0.06570999705	-6.97	106
**Cocarboxylase**	DB01987	NA	NA	NA	-6.88	8
**Lasofoxifene**	DB06202	NA	NA	NA	-6.84	9
**Hexachlorophene**	DB00756	-0.1205069314	-0.02762659079	-0.021927507	-6.83	69
**Benazepril**	DB00542	NA	NA	NA	-6.8	7
**Etoricoxib**	DB01628	-0.04561993738	-0.09469646296	-0.02849270576	-6.8	25
**Indomethacin**	DB00328	-0.06872835755	-0.1234924169	-0.05359016853	-6.73	19
**Homosalate**	DB11064	NA	NA	NA	-6.68	8
**Mitotane**	DB00648	-0.07788038982	-0.1279216274	-0.0534352127	-6.64	37
**Proguanil**	DB01131	-0.05906985523	-0.0986410706	-0.06488844299	-6.64	41
**Imatinib**	DB00619	-0.1275296881	-0.1406454315	-0.05471119522	-6.61	10
**Valdecoxib**	DB00580	-0.1174180274	-0.139154822	-0.05737104159	-6.57	131
**Baricitinib**	DB11817	-0.02509236501	-0.03395915629	-0.02316989406	-6.57	21
**Benzylpenicillin**	DB01053	-0.05151804782	-0.1205224642	-0.04497655044	-6.54	46
**Ruxolitinib**	DB08877	-0.103105195	-0.1131754173	-0.08494928103	-6.49	38
**Rofecoxib**	DB00533	-0.06532970378	-0.1390964269	-0.06670270062	-6.48	63
**Netarsudil**	DB13931	NA	NA	NA	-6.46	12
**Zonisamide**	DB00909	NA	NA	NA	-6.45	16
**Pomalidomide**	DB08910	-0.01624233519	-0.02789451725	-0.02620193498	-6.45	54
**Arzoxifene**	DB06249	NA	NA	NA	-6.41	2
**Lenalidomide**	DB00480	-0.06385406488	-0.1404006454	-0.08674137477	-6.4	25
**Vandetanib**	DB05294	-0.02622700096	-0.0392738498	-0.02858052382	-6.39	64
**Camphor**	DB01744	NA	NA	NA	-6.35	73
**Donepezil**	DB00843	-0.07815528694	-0.08555866665	-0.04839647863	-6.33	42
**Lenvatinib**	DB09078	-0.02085713306	-0.03670757124	-0.03819902793	-6.33	32
**Galantamine**	DB00674	-0.04982630031	-0.1281839865	-0.06054166546	-6.32	56
**Thalidomide**	DB01041	-0.08657364237	-0.1593972746	-0.08730739799	-6.27	15

Drug name	Drug ID	DS1 PC	DS2 PC	DS3 PC	Autodock LE	Autodock cluster
**Oxaprozin**	DB00991	-0.0783222472	-0.1521492916	-0.09516869794	-6.24	70
**Desipramine**	DB01151	-0.0626465238	-0.1244359823	-0.06518023426	-6.2	49
**Pioglitazone**	DB01132	-0.08437085738	-0.1610229985	-0.08492279365	-6.19	16
**Permethrin**	DB04930	NA	NA	NA	-6.19	12
**Meclofenamic acid**	DB00939	-0.0711683043	-0.1137762251	-0.07724600644	-6.03	23
**Dasatinib**	DB01254	-0.03959075682	-0.1464677954	-0.09185735897	-6.03	18
**Chlorpromazine**	DB00477	-0.09631391962	-0.1572395409	-0.06452754988	-6.02	84
**Etodolac**	DB00749	-0.03718207416	-0.1263476927	-0.08220971901	-6.01	23
**Bendazac**	DB13501	NA	NA	NA	-5.88	27
**Nomifensine**	DB04821	-0.03876006779	-0.1043832671	-0.08165269248	-5.87	130
**Cefprozil**	DB01150	NA	NA	NA	-5.83	31
**Enzacamene**	DB11219	NA	NA	NA	-5.82	118
**Dobutamine**	DB00841	-0.08435685642	-0.1260907777	-0.06239967631	-5.81	16
**Isocarboxazid**	DB01247	-0.04975326092	-0.1163862569	-0.06504677419	-5.81	9
**Diclofenac**	DB00586	-0.06267402209	-0.1425241791	-0.08212780643	-5.8	67
**Celecoxib**	DB00482	-0.09092385036	-0.1448463125	-0.07540573835	-5.78	12
**Phenylbutazone**	DB00812	-0.03669629969	-0.2123980944	-0.09277102578	-5.78	25
**Ketamine**	DB01221	NA	NA	NA	-5.75	86
**Spirapril**	DB01348	NA	NA	NA	-5.69	2
**Ketorolac**	DB00465	-0.06601382618	-0.1489144421	-0.07699282352	-5.68	92
**Minaprine**	DB00805	-0.02485426552	-0.1302259838	-0.07751564536	-5.68	27
**Zimelidine**	DB04832	NA	NA	NA	-5.67	24
**Bromfenac**	DB00963	-0.03038903046	-0.07734959514	-0.06200072483	-5.66	7
**Physostigmine**	DB00981	-0.05175476108	-0.1478856324	-0.07026511377	-5.66	19
**Ramipril**	DB00178	-0.07548091718	-0.1278868792	-0.07810335242	-5.64	6
**Sulfasalazine**	DB00795	NA	NA	NA	-5.64	25
**Tiaprofenic acid**	DB01600	-0.04855219047	-0.05210586323	-0.07681435882	-5.64	34
**Menthyl salicylate**	DB11201	NA	NA	NA	-5.61	30
**Nimesulide**	DB04743	-0.05493930538	-0.1138073474	-0.06126457811	-5.59	6
**Metyrapone**	DB01011	-0.03354450516	-0.1500441948	-0.08878627781	-5.58	10
**Afatinib**	DB08916	-0.05469023142	-0.1569087453	-0.0476744751	-5.58	5
**Tofacitinib**	DB08895	-0.03848190173	-0.09969335877	-0.08081765762	-5.54	30

Drug name	Drug ID	DS1 PC	DS2 PC	DS3 PC	Autodock LE	Autodock cluster
**Tolmetin**	DB00500	-0.0828485655	-0.1572255765	-0.07010605108	-5.53	11
**Loxoprofen**	DB09212	-0.05426943458	-0.1141684645	-0.05005096196	-5.46	102
**Menadione**	DB00170	-0.1453822115	-0.1355622276	-0.07575705865	-5.45	28
**Lumiracoxib**	DB01283	NA	NA	NA	-5.45	35
**Dexketoprofen**	DB09214	NA	NA	NA	-5.45	2
**Ketoprofen**	DB01009	-0.06965267839	-0.1522945923	-0.0876240037	-5.44	13
**Enalapril**	DB00584	NA	NA	NA	-5.43	3
**Naproxen**	DB00788	-0.02031065294	-0.05251558703	-0.06314355619	-5.43	21
**Gemcitabine**	DB00441	-0.1197856961	-0.144233304	-0.05538734049	-5.41	5
**Flurbiprofen**	DB00712	-0.04757602254	-0.04423595102	-0.06243437665	-5.39	144
**Rasagiline**	DB01367	-0.01971289372	-0.03802722786	-0.01953619295	-5.36	1
**Enalaprilat**	DB09477	-0.06440736764	-0.0891707389	-0.07534522909	-5.36	1
**Nepafenac**	DB06802	-0.01789915777	-0.03172058721	-0.02354260654	-5.32	15
**Suprofen**	DB00870	-0.01875661461	-0.03681705366	-0.01692008136	-5.31	44
**Cilazapril**	DB01340	NA	NA	NA	-5.31	2
**Dipyrithione**	DB11327	NA	NA	NA	-5.24	19
**Tacrine**	DB00382	-0.09287031672	-0.08302414823	-0.06630365667	-5.21	1
**Ranitidine**	DB00863	-0.06662630567	-0.1474593113	-0.05935072968	-5.21	11
**Aceclofenac**	DB06736	-0.03724291835	-0.04663686721	0.003666648941	-5.2	1
**Tolfenamic acid**	DB09216	-0.03298036562	-0.03208419733	-0.02504916606	-5.19	79
**Cefdinir**	DB00535	-0.0258255418	-0.02941001403	-0.02565040918	-5.17	11
**Erlotinib**	DB00530	-0.09381046692	-0.1442473956	-0.07040092013	-5.16	8
**Cladribine**	DB00242	-0.1176725341	-0.1384668748	-0.0553976742	-5.15	9
**Lindane**	DB00431	NA	NA	NA	-5.13	117
**Melatonin**	DB01065	-0.05332669635	-0.1452299399	-0.08659236386	-5.13	33
**Proflavine**	DB01123	NA	NA	NA	-5.13	53
**Mefenamic acid**	DB00784	-0.08053264085	-0.1610880628	-0.09040675467	-5.11	78
**Nialamide**	DB04820	-0.04823406067	-0.1353793456	-0.06789379013	-5.08	23
**Diflunisal**	DB00861	-0.05325602118	-0.1474645017	-0.04859592628	-5.06	149
**Balsalazide**	DB01014	NA	NA	NA	-5.06	2
**Diethylstilbestrol**	DB00255	NA	NA	NA	-5.05	3
**Raltitrexed**	DB00293	-0.08206102395	-0.1586436186	-0.08207151651	-5.05	3
**Capecitabine**	DB01101	-0.04501451453	-0.1586436186	-0.06742698378	-5.05	48

Drug name	Drug ID	DS1 PC	DS2 PC	DS3 PC	Autodock LE	Autodock cluster
**Nabumetone**	DB00461	-0.03081962528	-0.1586436186	-0.07669459855	-5.03	64
**Fenoprofen**	DB00573	NA	NA	NA	-5.02	4
**Dienestrol**	DB00890	-0.0602286817	-0.1899624664	-0.07632484338	-5.02	5
**Moclobemide**	DB01171	-0.08176735304	-0.1085182704	-0.04853535362	-5.02	3
**Trifluridine**	DB00432	-0.05545399283	-0.1136605124	-0.0562147341	-4.99	9
**Floxuridine**	DB00322	-0.09416751706	-0.08941499712	-0.03464425628	-4.98	11
**Fluorouracil**	DB00544	-0.07334066692	-0.1090989338	-0.07021383781	-4.98	102
**Salsalate**	DB01399	-0.02229614783	-0.03075313934	-0.02406440909	-4.98	64
**Chloramphenicol**	DB00446	-0.004486549446	-0.08476272265	-0.03044854044	-4.9	12
**Dacomitinib**	DB11963	-0.03310816615	-0.03038494182	-0.02443712107	-4.89	12
**Tegafur**	DB09256	NA	NA	NA	-4.83	85
**Pyrimethamine**	DB00205	-0.07835576333	-0.1466342633	-0.07482209246	-4.81	27
**Isoprenaline**	DB01064	NA	NA	NA	-4.81	1
**Oxybenzone**	DB01428	-0.05085103307	-0.1425623805	-0.08471895184	-4.79	5
**Vorinostat**	DB02546	-0.1589620803	-0.1799903936	-0.09706307605	-4.79	44
**Trandolapril**	DB00519	NA	NA	NA	-4.77	2
**Osimertinib**	DB09330	-0.02506710417	-0.04465094636	-0.03109529258	-4.76	3
**Flufenamic acid**	DB02266	-0.03794344247	-0.1482318394	-0.0330936669	-4.68	45
**Neostigmine**	DB01400	-0.05841227851	-0.1346627898	-0.05182911851	-4.67	46
**Phenyl salicylate**	DB11071	NA	NA	NA	-4.65	11
**Mesalazine**	DB00244	NA	NA	NA	-4.64	5
**Tegafur-uracil**	DB09327	NA	NA	NA	-4.64	94
**Calcium Phosphate**	DB11348	NA	NA	NA	-4.63	16
**Phenol**	DB03255	NA	NA	NA	-4.61	149
**Acetaminophen**	DB00316	-0.0407834655	-0.1210933814	-0.04252458414	-4.58	6
**Ibuprofen**	DB01050	-0.06653703289	-0.1530301568	-0.06000440304	-4.57	14
**Antipyrine**	DB01435	-0.06423609069	-0.163852781	-0.09369368933	-4.57	2
**Resveratrol**	DB02709	-0.07453453575	-0.1569588619	-0.08585819831	-4.54	5
**Dexibuprofen**	DB09213	-0.04237383773	-0.1315091813	-0.05557828985	-4.54	13

Drug name	Drug ID	DS1 PC	DS2 PC	DS3 PC	Autodock LE	Autodock cluster
**Aminosalicylic acid**	DB00233	-0.02288280655	-0.1064705647	-0.04682085856	-4.5	19
**Alclofenac**	DB13167	NA	NA	NA	-4.5	83
**Caffeine**	DB00201	-0.07396416979	-0.1449457802	-0.08165567669	-4.49	147
**Lidocaine**	DB00281	-0.0528568396	-0.12199959	-0.07073725187	-4.49	25
**Quinapril**	DB00881	NA	NA	NA	-4.49	1
**Salicylic acid**	DB00936	NA	NA	NA	-4.49	43
**Timolol**	DB00373	-0.06960236247	-0.1682405832	-0.08729867794	-4.47	1
**Safinamide**	DB06654	-0.02830858562	-0.03012863409	-0.02532369029	-4.46	20
**Gefitinib**	DB00317	-0.07722806756	-0.1528584167	-0.08818786305	-4.42	5
**Amlodipine**	DB00381	-0.09589179185	-0.1279609055	-0.05497065618	-4.4	4
**Sulfadoxine**	DB01299	-0.0164610665	-0.07173518613	-0.07017371301	-4.4	7
**Tranylcypromine**	DB00752	NA	NA	NA	-4.38	141
**Captopril**	DB01197	-0.07362021	-0.1151497512	-0.07366461938	-4.37	37
**Edrophonium**	DB01010	-0.05870860654	-0.07766297865	-0.04997515091	-4.3	4
**Metamfetamine**	DB01577	NA	NA	NA	-4.29	3
**Acetohydroxamic acid**	DB00551	NA	NA	NA	-4.24	50
**Erdafitinib**	DB12147	NA	NA	NA	-4.23	3
**Pralidoxime**	DB00733	NA	NA	NA	-4.21	11
**Selegiline**	DB01037	NA	NA	NA	-4.19	1
**Pidolic acid**	DB03088	NA	NA	NA	-4.17	109
**Phentermine**	DB00191	-0.05959242774	-0.1754224209	-0.0609105017	-4.11	2
**Eugenol**	DB09086	-0.06666871845	-0.09662148661	-0.05256826742	-4.09	33

Drug name	Drug id	DS1 PC	DS2 PC	DS3 PC	Autodock LE	Autodock cluster
**Perindopril**	DB00790	-0.04672058301	-0.1156423864	-0.08355714744	-3.94	3
**Amphetamine**	DB00182	NA	NA	NA	-3.91	115
**Pyridostigmine**	DB00545	-0.01605146411	-0.1446496333	-0.07263672947	-3.91	23
**Ephedrine**	DB01364	NA	NA	NA	-3.9	108
**Rivastigmine**	DB00989	-0.04118877114	-0.08288883411	-0.01291249181	-3.87	4
**Bufexamac**	DB13346	-0.03237086481	-0.03754711732	-0.02197067876	-3.85	18
**Miglitol**	DB00491	-0.08052162269	-0.167591787	-0.06218658938	-3.81	1
**Acetylsalicylic acid**	DB00945	-0.03307493015	-0.1012026588	-0.07444387473	-3.79	113
**Glycol salicylate**	DB11323	NA	NA	NA	-3.71	13
**Trimethoprim**	DB00440	-0.02305001067	-0.02936002336	-0.05608023011	-3.68	16
**Choline**	DB00122	NA	NA	NA	-3.62	50
**Pargyline**	DB01626	-0.05079848604	-0.09896463587	-0.05224864791	-3.5	17
**Carbamoylcholine**	DB00411	NA	NA	NA	-3.44	97
**Acetylcholine**	DB03128	-0.04267333997	-0.09419844744	-0.07222182479	-3.44	117
**Isoflurophate**	DB00677	NA	NA	NA	-3.41	4
**Phenelzine**	DB00780	-0.07217048468	-0.08791322702	-0.07068957721	-3.4	105
**Guanidine**	DB00536	NA	NA	NA	-3.35	92
**Aspartic acid**	DB00128	NA	NA	NA	-3.32	4
**Cysteine**	DB00151	NA	NA	NA	-3.3	96
**Choline salicylate**	DB14006	NA	NA	NA	-3.3	58
**Glycerin**	DB09462	NA	NA	NA	-3.24	5
**Asparagine**	DB00174	NA	NA	NA	-3.2	4
**Levocarnitine**	DB00583	NA	NA	NA	-3.17	4
**Propanoic acid**	DB03766	NA	NA	NA	-3.17	150
**Citric acid**	DB04272	NA	NA	NA	-3.07	59
**Echothiophate**	DB01057	NA	NA	NA	-2.95	18
**Glycine**	DB00145	NA	NA	NA	-2.77	6
**Glutamic acid**	DB00142	NA	NA	NA	-2.75	11
**Calcium phosphate dihydrate**	DB14481	NA	NA	NA	-2	150
**Trolamine salicylate**	DB11079	NA	NA	NA	-1.97	1
**Zinc acetate**	DB14487	NA	NA	NA	-1.07	150

Supplementary Table 4: Transcriptomic and molecular docking analyses results for drugs with the potential of targeting the SARS-CoV-2 RNA dependent RNA polymerase (6M71).

PDB ID	Residue count	EC number	SCOPE fold	Classification	CATH description	Protein Family Annotation	Mean persistent similary
1I8L	668	NA	Immunoglobulin-Like Beta-Sandwich	Immune System	Immunoglobulins	Immunoglobulin V-Set Domain	0.900
1PYW	621	NA	Immunoglobulin-Like Beta-Sandwich	Immune System/Protein Binding/Toxin	Immunoglobulins	Immunoglobulin C1-Set Domain	0.904
4MVL	912	NA	NA	Protein Binding/Protein Fibril	NA	Lipocalin / Cytosolic Fatty-Acid Binding Protein Family	0.911
4QFD	694	1.4.3.3	NA	Oxidoreductase/Oxidoreductase Inhibitor	NA	Fad Dependent Oxidoreductase	0.904
4S0T	870	NA	NA	Transcription	NA	Ligand-Binding Domain Of Nuclear Hormone Receptor	0.909
5CS6	704	2.7.11.1	NA	Transferase	NA	Protein Kinase Domain	0.902
5KPN	704	2.4.2.30	NA	Transferase/Transferase Inhibitor	NA	Poly(Adp-Ribose) Polymerase Catalytic Domain	0.900
5KPP	700	2.4.2.30	NA	Transferase/Transferase Inhibitor	NA	Poly(Adp-Ribose) Polymerase Catalytic Domain	0.901
5KPQ	700	2.4.2.30	NA	Transferase/Transferase Inhibitor	NA	Poly(Adp-Ribose) Polymerase Catalytic Domain	0.902
5OTP	704	2.7.11.1	NA	Transferase	NA	Protein Kinase Domain	0.901
5WS0	704	2.4.2.30	NA	Transferase/Transferase Inhibitor	NA	Poly(Adp-Ribose) Polymerase Catalytic Domain	0.901
5WTC	704	2.4.2.30	NA	Transferase/Transferase Inhibitor	NA	Poly(Adp-Ribose) Polymerase Catalytic Domain	0.908
6BC1	666	NA	NA	Signaling Protein	NA	Ras Family	0.903

Supplementary Table 5: Proteins targeted by Drugbank FDA-approved medications showing average persistent similarity measures higher than 0.9 with 6W01.

Drug name	Drug id	PC set 1(GSE150316)	PC set 2 (CRA002390)	PC set 3 (GSE147507)	Autodock LE (kcal/mol)	Autodock cluster
Estradiol benzoate	DB13953	NA	NA	NA	-11.56	2
Clotrimazole	DB00257	-0.09247459429	-0.09417997645	-0.04845518428	-11.47	12
Dexamethasone	DB01234	-0.07176892746	-0.1529509474	-0.07847304485	-11.42	11
Phenolphthalein	DB04824	-0.1299268982	-0.09894230167	-0.03725859228	-11.15	25
Spironolactone	DB00421	-0.1200039446	-0.1046496564	-0.08907503297	-10.99	2
Prasterone	DB01708	NA	NA	NA	-10.98	40
Pregnenolone	DB02789	-0.0611673301	-0.09701467974	-0.05776315121	-10.77	44
Estradiol acetate	DB13952	NA	NA	NA	-10.61	23
Mifepristone	DB00834	-0.1336064601	-0.1414272927	-0.06288002847	-10.04	25
Niraparib	DB11793	NA	NA	NA	-9.85	36
Carbamazepine	DB00564	-0.08046486733	-0.1427960558	-0.07447221905	-9.66	32
Rucaparib	DB12332	-0.06746929306	-0.1769352724	-0.08890911592	-9.53	28
Sulfinpyrazone	DB01138	-0.08049934697	-0.1008229621	-0.07343952772	-9.5	2
Olaparib	DB09074	NA	NA	NA	-9.48	1
Rilpivirine	DB08864	-0.01779495312	-0.03036612398	-0.03165871921	-9.31	1
Methylphenobarbital	DB00849	NA	NA	NA	-9.28	74
Phenobarbital	DB01174	NA	NA	NA	-9.19	112
Triclosan	DB08604	-0.1338300494	-0.0825734045	-0.03948117551	-9.13	26
Flutemetamol (18F)	DB09151	NA	NA	NA	-8.91	129
Clonazepam	DB01068	-0.01622828602	-0.03401228995	-0.0298152316	-8.64	6
Phenytoin	DB00252	-0.03024144853	-0.1181536543	-0.08137144335	-8.59	18
Erlotinib	DB00530	-0.09381046692	-0.1442473956	-0.07040092013	-8.5	1

Drug name	Drug id	PC set 1(GSE150316)	PC set 2 (CRA002390)	PC set 3 (GSE147507)	Autodock LE (kcal/mol)	Autodock cluster
Mephenytoin	DB00532	-0.04907013636	-0.07908497205	-0.1006311735	-8.36	58
Miconazole	DB01110	-0.1202302847	-0.02711535835	-0.02795302676	-8.35	4
Permethrin	DB04930	NA	NA	NA	-8.26	1
Fenofibrate	DB01039	-0.06579908049	-0.1497709694	-0.06584586954	-8.21	16
Pentobarbital	DB00312	-0.0488779023	-0.04255962821	-0.03422718896	-8.08	32
Warfarin	DB00682	-0.05985786376	-0.1881027925	-0.07311862078	-8.05	1
Lindane	DB00431	NA	NA	NA	-8	19
Resveratrol	DB02709	-0.07453453575	-0.1569588619	-0.08585819831	-7.99	9
Econazole	DB01127	-0.04461210256	-0.1396819043	-0.06847544611	-7.93	6
Ethotoin	DB00754	-0.02095545218	-0.02810516847	-0.05989308745	-7.4	42
Talazoparib	DB11760	NA	NA	NA	-7.39	22
Bezafibrate	DB01393	-0.05022272695	-0.1735191963	-0.07664844941	-7.23	1
Fenofibric acid	DB13873	NA	NA	NA	-7.17	2
Ifosfamide	DB01181	-0.04544867364	-0.1517373733	-0.06720973109	-6.91	2
Theophylline	DB00277	-0.0674258857	-0.1042658312	-0.05348957409	-6.72	55
Cyclophosphamide	DB00531	-0.04964264496	-0.148458618	-0.06910821362	-6.34	27
Flutamide	DB00499	-0.06257285784	-0.1291467547	-0.08429414812	-5.9	15
Nicotinamide	DB02701	-0.06537166631	-0.1381641029	-0.04894040471	-5.76	73
Azathioprine	DB00993	-0.07268609978	-0.1879651099	-0.07933128967	-5.31	1
Diethylstilbestrol	DB00255	NA	NA	NA	-5	8
Dimercaprol	DB06782	NA	NA	NA	-4.05	42
Benzoic acid	DB03793	NA	NA	NA	-3.68	137
Nifedipine	DB01115	-0.06997637254	-0.1589009534	-0.07188197956	-2.59	12
Aluminum acetate	DB14518	NA	NA	NA	-1.21	86
Zinc acetate	DB14487	NA	NA	NA	-1.01	150

Supplementary Table 6: Transcriptomic and molecular docking analyses results for drugs with the potential of targeting the SARS-CoV-2 NSP15 Endoribonuclease (6W01).

3CL protease									
**Candidate drug**	**ID**	**Description**	**Set size**	**Enrichment score**	**NES**	**p-value**	**p-adjust**	**q-values**
Cholic acid	R-HSA-168274	Export of Viral Ribonucleoproteins from Nucleus	31	0.5504421479	2.164129742	4.44345700955343e-05	0.002159494061	0.00178604772
Cholic acid	R-HSA-168325	Viral Messenger RNA Synthesis	42	0.5068214292	2.156207981	4.97784857384638e-05	0.002159494061	0.00178604772
Cholic acid	R-HSA-192823	Viral mRNA Translation	85	0.47674678	2.356347972	7.38443361394181e-05	0.002159494061	0.00178604772
Cholic acid	R-HSA-168273	Influenza Viral RNA Transcription and Replication	130	0.4924408244	2.618170517	0.0001063942973	0.002441836331	0.002019563883
Cholic acid	R-HSA-9662851	Anti-inflammatory response favouring Leishmania parasite infection	140	-0.3902072615	-1.65818373	0.0003200354501	0.00520987942	0.004308922829
Cholic acid	R-HSA-9660821	ADORA2B mediated anti-inflammatory cytokines production	108	-0.4124621455	-1.697201332	0.0003684692011	0.005862010017	0.004848278961
Cholic acid	R-HSA-1169410	Antiviral mechanism by IFN-stimulated genes	77	0.3605928817	1.750131725	0.0007519824993	0.01052775499	0.008707165782
Cholic acid	R-HSA-1169408	ISG15 antiviral mechanism	69	0.3689047506	1.751465308	0.0008275510854	0.01135854431	0.009394284767
Rutin	R-HSA-168274	Export of Viral Ribonucleoproteins from Nucleus	31	0.5569809409	2.514532143	7.24375226367258e-05	0.001857379768	0.001207995112
Rutin	R-HSA-168325	Viral Messenger RNA Synthesis	42	0.6257977832	3.063251562	8.99280575539568e-05	0.001857379768	0.001207995112
Rutin	R-HSA-5676590	NIK–>noncanonical NF-kB signaling	56	0.5028596279	2.646049886	0.0001159554731	0.001857379768	0.001207995112
Rutin	R-HSA-5607761	Dectin-1 mediated noncanonical NF-kB signaling	57	0.5115624867	2.705496875	0.0001178550383	0.001857379768	0.001207995112
Rutin	R-HSA-1169091	Activation of NF-kappaB in B cells	64	0.4616018681	2.496165981	0.0001333511135	0.001857379768	0.001207995112
Rutin	R-HSA-1169408	ISG15 antiviral mechanism	69	0.4239371602	2.331862094	0.0001448855404	0.001857379768	0.001207995112
Rutin	R-HSA-2871837	FCERI mediated NF-kB activation	75	0.3861340167	2.15575237	0.0001607458608	0.001857379768	0.001207995112
Rutin	R-HSA-1169410	Antiviral mechanism by IFN-stimulated genes	77	0.3997968025	2.246800743	0.0001639613051	0.001857379768	0.001207995112
Rutin	R-HSA-9662851	Anti-inflammatory response favouring Leishmania parasite infection	140	-0.3800257431	-1.641123355	0.0002054348943	0.00202541445	0.001317280827
Rutin	R-HSA-9020702	Interleukin-1 signaling	94	0.3362458296	1.964845044	0.0002143622722	0.002055528638	0.00133686637
Rutin	R-HSA-3238698	WNT ligand biogenesis and trafficking	19	-0.6461670939	-1.989186587	0.0002228443293	0.002122326946	0.001380310382
Rutin	R-HSA-9660821	ADORA2B mediated anti-inflammatory cytokines production	108	-0.4002326642	-1.683341581	0.000336555758	0.002799602456	0.001820794079
Rutin	R-HSA-168273	Influenza Viral RNA Transcription and Replication	130	0.3406951661	2.111772556	0.0003617945007	0.00291415321	0.001895295133
Rutin	R-HSA-5668541	TNFR2 non-canonical NF-kB pathway	93	0.3187363572	1.857850168	0.0004231013328	0.003254625637	0.002116730207
Rutin	R-HSA-8950505	Gene and protein expression by JAK-STAT signaling after Interleukin-12 stimulation	35	0.4205242683	1.969330941	0.001322957198	0.00755975542	0.004916683036
Rutin	R-HSA-9679506	SARS-CoV Infections	131	0.2656706236	1.646723675	0.0014803849	0.008175514895	0.005317158184
Rutin	R-HSA-446652	Interleukin-1 family signaling	126	0.2530289061	1.561771852	0.001715265866	0.009096106866	0.005915889052
Rutin	R-HSA-9020591	Interleukin-12 signaling	44	0.3510323101	1.737816087	0.004107542942	0.01885429547	0.01226238014
Rutin	R-HSA-447115	Interleukin-12 family signaling	52	0.3043832231	1.570910759	0.009667608082	0.03812577835	0.02479608893
Rutin	R-HSA-9679191	Potential therapeutics for SARS	74	0.2713525038	1.512977379	0.009813232035	0.0384799498	0.02502643351
Rutin	R-HSA-209560	NF-kB is activated and signals survival	13	0.5236055278	1.800037483	0.01221948613	0.04513794349	0.02935663242
Sulindac	R-HSA-5676590	NIK–>noncanonical NF-kB signaling	56	0.4996312573	2.170976078	4.02349722378692e-05	0.002711160687	0.002091466815
Sulindac	R-HSA-5607761	Dectin-1 mediated noncanonical NF-kB signaling	57	0.5100412224	2.224029415	4.05153553196662e-05	0.002711160687	0.002091466815
Sulindac	R-HSA-9660821	ADORA2B mediated anti-inflammatory cytokines production	108	-0.4372180691	-1.871071118	9.9644128113879e-05	0.003278197179	0.002528894967
Sulindac	R-HSA-1169410	Antiviral mechanism by IFN-stimulated genes	77	0.3908637601	1.811891075	0.0005332622317	0.00957137339	0.0073836309
Sulindac	R-HSA-1169091	Activation of NF-kappaB in B cells	64	0.4058740207	1.812595358	0.001050552591	0.01598666986	0.01233257389
Sulindac	R-HSA-9662851	Anti-inflammatory response favouring Leishmania parasite infection	140	-0.3640246568	-1.614235234	0.001323845772	0.01872105132	0.01444195388
Sulindac	R-HSA-5668541	TNFR2 non-canonical NF-kB pathway	93	0.3412820726	1.643943657	0.002704626335	0.02981477849	0.02299997198
Sulindac	R-HSA-1169408	ISG15 antiviral mechanism	69	0.3760216788	1.706866142	0.002785276599	0.03022780806	0.02331859479
Sulindac	R-HSA-2871837	FCERI mediated NF-kB activation	75	0.3629367679	1.673597969	0.003009249015	0.0316192247	0.02439197334
Sulindac	R-HSA-9020702	Interleukin-1 signaling	94	0.3322636217	1.604201166	0.003850908054	0.0366753148	0.0282923857
Sulfafurazole	R-HSA-9660821	ADORA2B mediated anti-inflammatory cytokines production	108	-0.4306193538	-1.825004033	3.73055448474825e-05	0.001029873693	0.0006883892582
Sulfafurazole	R-HSA-9662851	Anti-inflammatory response favouring Leishmania parasite infection	140	-0.4197075671	-1.836736506	4.83512232859491e-05	0.001029873693	0.0006883892582
Sulfafurazole	R-HSA-168325	Viral Messenger RNA Synthesis	42	0.5102774546	2.278239823	5.47705115565779e-05	0.001029873693	0.0006883892582
Sulfafurazole	R-HSA-9678108	SARS-CoV-1 Infection	42	0.5180480874	2.312933429	5.47705115565779e-05	0.001029873693	0.0006883892582
Sulfafurazole	R-HSA-5676590	NIK–>noncanonical NF-kB signaling	56	0.4500133485	2.149550825	6.33673404727204e-05	0.001029873693	0.0006883892582
Sulfafurazole	R-HSA-5607761	Dectin-1 mediated noncanonical NF-kB signaling	57	0.4717460974	2.262212277	6.42425799820121e-05	0.001029873693	0.0006883892582
Sulfafurazole	R-HSA-9694516	SARS-CoV-2 Infection	59	0.4534124748	2.19100229	6.5772165219679e-05	0.001029873693	0.0006883892582
Sulfafurazole	R-HSA-1169091	Activation of NF-kappaB in B cells	64	0.4113534726	2.023081707	6.97009827838573e-05	0.001029873693	0.0006883892582
Sulfafurazole	R-HSA-192823	Viral mRNA Translation	85	0.8647064394	4.497675929	8.48608282416836e-05	0.001031271086	0.0006893233047
Sulfafurazole	R-HSA-168273	Influenza Viral RNA Transcription and Replication	130	0.7708064983	4.331049731	0.0001253604112	0.001262622846	0.0008439636921
Sulfafurazole	R-HSA-209560	NF-kB is activated and signals survival	13	0.7380042107	2.346446175	0.0001856045139	0.001596757214	0.001067306138
Sulfafurazole	R-HSA-193639	p75NTR signals via NF-kB	16	0.6439705868	2.19322002	0.0001962092375	0.001654776702	0.001106087585
Sulfafurazole	R-HSA-2871837	FCERI mediated NF-kB activation	75	0.3562834389	1.809860239	0.0003883495146	0.002861522739	0.001912702041
Sulfafurazole	R-HSA-9679506	SARS-CoV Infections	131	0.2892214101	1.626126132	0.000508001016	0.003610159505	0.002413106616
Sulfafurazole	R-HSA-1169408	ISG15 antiviral mechanism	69	0.3388176757	1.693232294	0.001620745543	0.009299359673	0.006215887781
Sulfafurazole	R-HSA-8950505	Gene and protein expression by JAK-STAT signaling after Interleukin-12 stimulation	35	0.4355614264	1.85504697	0.001970692269	0.01112487571	0.007436101133
Sulfafurazole	R-HSA-1169410	Antiviral mechanism by IFN-stimulated genes	77	0.3114963289	1.592532992	0.002595359811	0.01350744883	0.009028663166
Sulfafurazole	R-HSA-9020702	Interleukin-1 signaling	94	0.2902885679	1.537551592	0.003333642004	0.01660889255	0.01110173344
Sulfafurazole	R-HSA-168274	Export of Viral Ribonucleoproteins from Nucleus	31	0.4310027981	1.779404701	0.004099546638	0.01985939548	0.01327443803
Sulfafurazole	R-HSA-68884	Mitotic Telophase/Cytokinesis	13	0.5923137743	1.883230976	0.006162069862	0.02810064432	0.01878306226
Sulfafurazole	R-HSA-447115	Interleukin-12 family signaling	52	0.3366313237	1.581831635	0.009289053488	0.03836187281	0.02564188341
Dasatinib	R-HSA-9660821	ADORA2B mediated anti-inflammatory cytokines production	108	-0.4590362984	-1.867333177	2.51863792061253e-05	0.005876821815	0.005434955513
Dasatinib	R-HSA-9662851	Anti-inflammatory response favouring Leishmania parasite infection	140	-0.3852605708	-1.616602947	0.0008056247254	0.04699477565	0.04346133387

Supplementary Table 7: GSEA results for top drugs targeting the 3CL protease (6M2Q)

**RNA-dependent RNA polymerase (NSP12)**								
**Candidate drug**	ID	Description	**Set size**	**Enrichment score**	**NES**	**p-value**	**p-adjust**	**q-values**
Vemurafenib	R-HSA-1169410	Antiviral mechanism by IFN-stimulated genes	77	0.406581833606254	1.94257871020949	7.17591762046572e-05	0.00522916830078176	0.00460795883347085
Vemurafenib	R-HSA-1169408	ISG15 antiviral mechanism	69	0.40901832833856	1.90960217162075	0.000170120104793985	0.00850600523969923	0.00749551739919361
Vemurafenib	R-HSA-168273	Influenza Viral RNA Transcription and Replication	130	0.308179865093806	1.61361511760422	0.000810988899589437	0.0252307657650047	0.0222334266741244
Sorafenib	R-HSA-9660821	ADORA2B mediated anti-inflammatory cytokines production	108	-0.453523422493337	-1.94021226408423	8.72120910843079e-06	0.000678831323992614	0.000539492262752025
Sorafenib	R-HSA-192823	Viral mRNA Translation	85	0.629603412689939	2.95410295613698	2.57506308904568e-05	0.00074937574324246	0.000595556511734797
Sorafenib	R-HSA-168273	Influenza Viral RNA Transcription and Replication	130	0.543925333373416	2.74880523032984	3.03573054855651e-05	0.000758932637139128	0.00060315172741057
Sorafenib	R-HSA-9662851	Anti-inflammatory response favouring Leishmania parasite infection	140	-0.390591425899787	-1.73464079134822	0.000135434830452522	0.00223069132510036	0.00177281257942186
Levonorgestrel	R-HSA-9660821	ADORA2B mediated anti-inflammatory cytokines production	108	-0.453087343563133	-1.81709723888399	2.16993482962395e-05	0.000995033362047153	0.000712234406517962
Levonorgestrel	R-HSA-5676590	NIK–>noncanonical NF-kB signaling	56	0.569886693839874	2.64911999403131	4.64921660700172e-05	0.000995033362047153	0.000712234406517962
Levonorgestrel	R-HSA-5607761	Dectin-1 mediated noncanonical NF-kB signaling	57	0.577349130224171	2.6933633064629	4.71120324130783e-05	0.000995033362047153	0.000712234406517962
Levonorgestrel	R-HSA-1169091	Activation of NF-kappaB in B cells	64	0.494622347300901	2.36383615761675	5.11665984445354e-05	0.000995033362047153	0.000712234406517962
Levonorgestrel	R-HSA-1169408	ISG15 antiviral mechanism	69	0.428852654813679	2.08203863728266	5.42475859824238e-05	0.000995033362047153	0.000712234406517962
Levonorgestrel	R-HSA-2871837	FCERI mediated NF-kB activation	75	0.433326167735557	2.1416776581742	5.86303939962477e-05	0.000995033362047153	0.000712234406517962
Levonorgestrel	R-HSA-192823	Viral mRNA Translation	85	0.655605018572043	3.31485267520204	6.57548658600736e-05	0.000995033362047153	0.000712234406517962
Levonorgestrel	R-HSA-5668541	TNFR2 non-canonical NF-kB pathway	93	0.386441281935432	1.98764878523195	7.1694866647548e-05	0.000995033362047153	0.000712234406517962
Levonorgestrel	R-HSA-9020702	Interleukin-1 signaling	94	0.367207724197035	1.89240574447968	7.24952878062926e-05	0.000995033362047153	0.000712234406517962
Levonorgestrel	R-HSA-168273	Influenza Viral RNA Transcription and Replication	130	0.602685988089197	3.28182083703367	0.000106849022331446	0.00115068177895403	0.000823645904935517
Levonorgestrel	R-HSA-1169410	Antiviral mechanism by IFN-stimulated genes	77	0.392188744918012	1.94631068899511	0.000120307988450433	0.00122051582485947	0.000873632379899408
Levonorgestrel	R-HSA-168325	Viral Messenger RNA Synthesis	42	0.460052305619905	2.0022540544456	0.000272458352794644	0.00235457835748458	0.00168538240325212
Levonorgestrel	R-HSA-9662851	Anti-inflammatory response favouring Leishmania parasite infection	140	-0.392665725203299	-1.62074231763402	0.00038851411012609	0.00322766145388363	0.00231032609330618
Levonorgestrel	R-HSA-3858494	Beta-catenin independent WNT signaling	132	0.290978062858633	1.58775986774646	0.000875944377532027	0.00662876826240453	0.00474480254572114
Levonorgestrel	R-HSA-5099900	WNT5A-dependent internalization of FZD4	14	0.643351140854843	2.06864366184519	0.00198166955660144	0.0127263182534037	0.00910936464454161
Levonorgestrel	R-HSA-446652	Interleukin-1 family signaling	126	0.278850803225048	1.50950052185097	0.00279127468210483	0.016699933995499	0.0119536369651993
Levonorgestrel	R-HSA-9679506	SARS-CoV Infections	131	0.259226404719143	1.41329932175509	0.00800432666306111	0.0378583017847485	0.0270985739090831
Levonorgestrel	R-HSA-5140745	WNT5A-dependent internalization of FZD2. FZD5 and ROR2	12	0.609947800813526	1.86839878244653	0.00907018821815448	0.0419084604139151	0.0299976348225918
Raloxifene	R-HSA-168274	Export of Viral Ribonucleoproteins from Nucleus	31	0.605171403210317	2.49461268431468	3.30622231038815e-05	0.000698380107341023	0.000486765683838442
Raloxifene	R-HSA-168325	Viral Messenger RNA Synthesis	42	0.602176676765964	2.68356349766003	3.88033060416748e-05	0.000698380107341023	0.000486765683838442
Raloxifene	R-HSA-5676590	NIK–>noncanonical NF-kB signaling	56	0.606307873703184	2.88719165837004	4.69792351780513e-05	0.000698380107341023	0.000486765683838442
Raloxifene	R-HSA-5607761	Dectin-1 mediated noncanonical NF-kB signaling	57	0.603784485054598	2.88438114995461	4.76235831983998e-05	0.000698380107341023	0.000486765683838442
Raloxifene	R-HSA-1169091	Activation of NF-kappaB in B cells	64	0.589865818118373	2.88643023502572	5.19966722129784e-05	0.000698380107341023	0.000486765683838442
Raloxifene	R-HSA-2871837	FCERI mediated NF-kB activation	75	0.549041872593982	2.77815047634368	5.92417061611374e-05	0.000698380107341023	0.000486765683838442
Raloxifene	R-HSA-192823	Viral mRNA Translation	85	0.766484033530631	3.96950847020697	6.68851581833991e-05	0.000698380107341023	0.000486765683838442
Raloxifene	R-HSA-5668541	TNFR2 non-canonical NF-kB pathway	93	0.391300936357624	2.06165657320156	7.27749072119933e-05	0.000698380107341023	0.000486765683838442
Raloxifene	R-HSA-9020702	Interleukin-1 signaling	94	0.42015800127342	2.21704751137723	7.33299112708074e-05	0.000698380107341023	0.000486765683838442
Raloxifene	R-HSA-9660821	ADORA2B mediated anti-inflammatory cytokines production	108	-0.436976428791342	-1.77770325909326	7.94631182772396e-05	0.000713130548641894	0.000497046630519576
Raloxifene	R-HSA-168273	Influenza Viral RNA Transcription and Replication	130	0.694969213984698	3.87184041152553	0.000109170305676856	0.00080441277867157	0.000560669658517703
Raloxifene	R-HSA-1169408	ISG15 antiviral mechanism	69	0.414562046032442	2.06143901082339	0.000110920082080861	0.000812713627582107	0.00056645528779595
Raloxifene	R-HSA-3858494	Beta-catenin independent WNT signaling	132	0.367572106418147	2.05259728263026	0.000111457868925546	0.000812713627582107	0.00056645528779595
Raloxifene	R-HSA-1169410	Antiviral mechanism by IFN-stimulated genes	77	0.38107989667861	1.93746883861453	0.000183049606443346	0.00112010573798167	0.000780705277525567
Raloxifene	R-HSA-446652	Interleukin-1 family signaling	126	0.304987424495168	1.69020073899593	0.00021117094287826	0.00127705183462179	0.000890095526837892
Raloxifene	R-HSA-201681	TCF dependent signaling in response to WNT	173	0.26881714593418	1.55663981191347	0.000518941359626362	0.00279429962875733	0.00194760583147222
Raloxifene	R-HSA-9662851	Anti-inflammatory response favouring Leishmania parasite infection	140	-0.385104328959152	-1.61201104962118	0.000535803670255141	0.00286307304716488	0.0019955403870089
Raloxifene	R-HSA-195721	Signaling by WNT	255	0.227862984774869	1.38930277839498	0.00236220472440945	0.0104324498869818	0.00727133913175348
Raloxifene	R-HSA-8950505	Gene and protein expression by JAK-STAT signaling after Interleukin-12 stimulation	35	0.406989802714876	1.73214027849819	0.00453443003269008	0.0184540757144364	0.0128623520205132
Raloxifene	R-HSA-9020591	Interleukin-12 signaling	44	0.36292617362108	1.63571879013351	0.00644103056489038	0.0255451637134463	0.017804787039372
Raloxifene	R-HSA-447115	Interleukin-12 family signaling	52	0.341316965276608	1.59974801443277	0.00700173928555501	0.0276904943496526	0.0193000663625022
Raloxifene	R-HSA-909733	Interferon alpha/beta signaling	69	-0.396151219313152	-1.51786623045219	0.0126542952618378	0.0458964076854222	0.0319894510709672

Supplementary Table 8: GSEA results for top drugs targeting the RNA-dependent RNA polymerase (NSP12)(6M71)

Endoribonuclease (NSP15)								
**Candidate drug**	**ID**	**Description**	**Set size**	**Enrichment score**	**NES**	**p-value**	**p-adjust**	**q-values**
Dexamethasone	R-HSA-9660821	ADORA2B mediated anti-inflammatory cytokines production	108	-0.471477511726897	-1.87570091093215	1.13948426941966e-05	0.00142723885351942	0.000944338489546683
Dexamethasone	R-HSA-9662851	Anti-inflammatory response favouring Leishmania parasite infection	140	-0.419911576651085	-1.7075467323971	2.25418150669492e-05	0.00210390273958192	0.00139205594799406
Dexamethasone	R-HSA-168274	Export of Viral Ribonucleoproteins from Nucleus	31	0.611649876017228	2.75690770473583	8.99361453368109e-05	0.0021467190466114	0.00142038553460003
Dexamethasone	R-HSA-168325	Viral Messenger RNA Synthesis	42	0.602186196773026	2.94025297194721	0.000115874855156431	0.0021467190466114	0.00142038553460003
Dexamethasone	R-HSA-5676590	NIK–>noncanonical NF-kB signaling	56	0.411270780003163	2.14635606918885	0.000160230732254446	0.0021467190466114	0.00142038553460003
Dexamethasone	R-HSA-5607761	Dectin-1 mediated noncanonical NF-kB signaling	57	0.417782317608317	2.18791868132162	0.000163907556138338	0.0021467190466114	0.00142038553460003
Dexamethasone	R-HSA-192823	Viral mRNA Translation	85	0.502118366025727	2.8474762221436	0.000286944045911047	0.00250327511827975	0.00165630233389938
Dexamethasone	R-HSA-1169408	ISG15 antiviral mechanism	69	0.333776736342806	1.81832252157487	0.000413223140495868	0.00316896945113449	0.00209676174210402
Dexamethasone	R-HSA-2871837	FCERI mediated NF-kB activation	75	0.330265587701427	1.82929876670774	0.000469263256687001	0.00335660580021482	0.00222091210841281
Dexamethasone	R-HSA-1169091	Activation of NF-kappaB in B cells	64	0.338206529337173	1.81546995193578	0.000564440263405456	0.00383600179013417	0.00253810644760757
Dexamethasone	R-HSA-168273	Influenza Viral RNA Transcription and Replication	130	0.535633669485802	3.28090684949883	0.00066577896138482	0.00431523400897569	0.00285519242699143
Dexamethasone	R-HSA-909733	Interferon alpha/beta signaling	69	-0.445011867083094	-1.6813820109872	0.000927643784786642	0.00547975231519535	0.00362570077997888
Dexamethasone	R-HSA-1169410	Antiviral mechanism by IFN-stimulated genes	77	0.285704187494755	1.59306267573016	0.00242365487154629	0.011989812085388	0.00793310874822666
Dexamethasone	R-HSA-4839735	AXIN mutants destabilize the destruction complex. activating WNT signaling	13	0.566297733305241	1.9398739298277	0.00511576393650124	0.021653747184866	0.0143272913704377
Dexamethasone	R-HSA-1266695	Interleukin-7 signaling	23	-0.529515770891534	-1.64481373536237	0.00887066130321414	0.0346897369399436	0.022952607900113
Dexamethasone	R-HSA-8950505	Gene and protein expression by JAK-STAT signaling after Interleukin-12 stimulation	35	0.34710911872055	1.61792913011669	0.0109452736318408	0.0415019810923063	0.0274599574144583
Dexamethasone	R-HSA-5668541	TNFR2 non-canonical NF-kB pathway	93	0.242503602555991	1.40434249270299	0.011768661735037	0.0440538139814219	0.029148388198234
Phenolphthalein	R-HSA-168273	Influenza Viral RNA Transcription and Replication	130	-0.734808136077605	-3.14957180402213	1.65005610190746e-05	0.000207862580266507	0.000134407382728719
Phenolphthalein	R-HSA-192823	Viral mRNA Translation	85	-0.803860900521987	-3.22507922269736	1.72562553925798e-05	0.000207862580266507	0.000134407382728719
Phenolphthalein	R-HSA-1169408	ISG15 antiviral mechanism	69	-0.486093116955364	-1.87816069268596	8.80080263320015e-05	0.000720533548916971	0.000465908911329771
Phenolphthalein	R-HSA-1169410	Antiviral mechanism by IFN-stimulated genes	77	-0.46729998470842	-1.84159155653004	0.000139435981455014	0.00106512981396409	0.000688730556397832
Phenolphthalein	R-HSA-5676590	NIK–>noncanonical NF-kB signaling	56	-0.507065603306953	-1.88314619071709	0.000286902883373978	0.00197864057499295	0.00127942172518341
Phenolphthalein	R-HSA-449147	Signaling by Interleukins	409	0.268573809873669	1.43229999756256	0.000402860308188136	0.00267300678418668	0.00172841040180492
Phenolphthalein	R-HSA-168325	Viral Messenger RNA Synthesis	42	-0.537305345776849	-1.88341630019971	0.000495212941564873	0.00315135508268555	0.00203771832414254
Phenolphthalein	R-HSA-5607761	Dectin-1 mediated noncanonical NF-kB signaling	57	-0.49247317102619	-1.83615309892325	0.000572113063844242	0.00354406322735371	0.00229164990640917
Phenolphthalein	R-HSA-6785807	Interleukin-4 and Interleukin-13 signaling	104	0.374559414869498	1.67244802551366	0.000937146550331233	0.0052480206818549	0.00339345698225204
Phenolphthalein	R-HSA-168274	Export of Viral Ribonucleoproteins from Nucleus	31	-0.543112552605167	-1.78078847785057	0.00278634876110332	0.0123837722715703	0.00800755199515073
Phenolphthalein	R-HSA-448424	Interleukin-17 signaling	65	0.394120551294936	1.61585792923176	0.00616102404593851	0.0235028725122923	0.015197346135768
Phenolphthalein	R-HSA-5668541	TNFR2 non-canonical NF-kB pathway	93	-0.381513325406234	-1.55270841737834	0.00665628582673123	0.0250505380575907	0.0161980922778406
Phenolphthalein	R-HSA-4791275	Signaling by WNT in cancer	31	0.474742317052852	1.65104462343028	0.012456538998549	0.0421235618308421	0.0272377918605445
Spironolactone	R-HSA-168325	Viral Messenger RNA Synthesis	42	-0.607686036666276	-2.46723663517616	4.13479429398387e-05	0.000573166787441097	0.000403802466039329
Spironolactone	R-HSA-5676590	NIK–>noncanonical NF-kB signaling	56	-0.471164851310287	-2.04221383918887	4.51834447858305e-05	0.000573166787441097	0.000403802466039329
Spironolactone	R-HSA-1169410	Antiviral mechanism by IFN-stimulated genes	77	-0.439939317070795	-2.03263496921474	5.05178075271533e-05	0.000573166787441097	0.000403802466039329
Spironolactone	R-HSA-192823	Viral mRNA Translation	85	-0.406893918695999	-1.91840721723034	5.2534804307854e-05	0.000573166787441097	0.000403802466039329
Spironolactone	R-HSA-168273	Influenza Viral RNA Transcription and Replication	130	-0.487261048942222	-2.47827948941961	6.50195058517555e-05	0.000619233389064338	0.000436256906431041
Spironolactone	R-HSA-5607761	Dectin-1 mediated noncanonical NF-kB signaling	57	-0.466265935834548	-2.02791577478152	9.07935355002724e-05	0.000710117037432298	0.000500285461709822
Spironolactone	R-HSA-1169408	ISG15 antiviral mechanism	69	-0.426235503276121	-1.92525857314502	9.72478848585043e-05	0.000748060652757726	0.000527017166642097
Spironolactone	R-HSA-168274	Export of Viral Ribonucleoproteins from Nucleus	31	-0.568183028077224	-2.13976468379911	0.000116004794864854	0.000863082272290127	0.000608051194838984
Spironolactone	R-HSA-913531	Interferon Signaling	182	-0.308699366886192	-1.65026397284668	0.000162866449511401	0.0011400651465798	0.000803188753643065
Spironolactone	R-HSA-1169091	Activation of NF-kappaB in B cells	64	-0.375793573615333	-1.67411189942594	0.00362762649580703	0.0183345743470391	0.0129169144084028
Spironolactone	R-HSA-9660821	ADORA2B mediated anti-inflammatory cytokines production	108	0.374013015756146	1.56841557232671	0.00372266170311773	0.0187472172099454	0.0132076259591871
Spironolactone	R-HSA-2871837	FCERI mediated NF-kB activation	75	-0.347361789593439	-1.59839901888183	0.00483501146446017	0.023023864116477	0.0162205719376985
Spironolactone	R-HSA-909733	Interferon alpha/beta signaling	69	-0.35101936654178	-1.58551561186282	0.00602936886122727	0.0275853477311051	0.0194341885895079
Spironolactone	R-HSA-68884	Mitotic Telophase/Cytokinesis	13	-0.611341403855248	-1.80849902401457	0.00968998701341947	0.0406167120322972	0.0286149317099718
Mifepristone	R-HSA-1169408	ISG15 antiviral mechanism	69	0.358074615543641	1.77434154584096	0.000536078052964512	0.0206833211175888	0.0184750266824778
Mifepristone	R-HSA-1169410	Antiviral mechanism by IFN-stimulated genes	77	0.327670020734597	1.66051471525399	0.00132402503610977	0.0378292867459935	0.033790370416722
Carbamazepine	R-HSA-9662851	Anti-inflammatory response favouring Leishmania parasite infection	140	-0.422455596963905	-1.80348774668326	1.13247718058481e-05	0.00135624088775654	0.00106153892041696
Carbamazepine	R-HSA-9660821	ADORA2B mediated anti-inflammatory cytokines production	108	-0.46934122580356	-1.95508155679512	1.14714418455256e-05	0.00135624088775654	0.00106153892041696
Carbamazepine	R-HSA-168274	Export of Viral Ribonucleoproteins from Nucleus	31	0.586167427094314	2.69302482424876	8.34167500834168e-05	0.00341997264021888	0.00267683572817132
Carbamazepine	R-HSA-168325	Viral Messenger RNA Synthesis	42	0.624453496035066	3.11598581830055	0.000103874519580347	0.00341997264021888	0.00267683572817132
Carbamazepine	R-HSA-1169408	ISG15 antiviral mechanism	69	0.469530361379513	2.63036504705283	0.000175654312313367	0.00352552479954873	0.00275945211754153
Carbamazepine	R-HSA-1169410	Antiviral mechanism by IFN-stimulated genes	77	0.489620094845069	0.489620094845069	0.000204123290467442	0.00376016587703183	0.00294310727668431
Carbamazepine	R-HSA-192823	Viral mRNA Translation	85	0.569593747617224	3.32843929163762	0.000240442414041837	0.0039579552072555	0.00309791832387442
Carbamazepine	R-HSA-168273	Influenza Viral RNA Transcription and Replication	130	0.568120009422238	3.58424407798979	0.000510204081632653	0.00624191198681173	0.00488558675057971
Carbamazepine	R-HSA-913531	Interferon Signaling	182	0.211050398272419	1.4100753845135	0.0058411214953271	0.0393152408339324	0.030772305043702

Supplementary Table 9: GSEA results for top drugs targeting the SP15 Endoribonuclease (6W01)

Drug name	**Protein target**	Tested against SARs-COV-2 / COVID patients	Suggested as candidate by Repurposing strategies	Used against other SARS/MERS	Already in Clinical Trials
Cholic Acid	3CLPro	Yes [1]	Yes [2]	No	No
Rutin	3CLPro	No	Yes [3, 4, 5, 6, 7, 8, 9, 10, 11]	No	No
Indomethacin	3CLPro	Yes [12]	Yes [13, 14]	Yes [15]	2 clinical trials
Sulindac	3CLPro	No	No	No	No
Sulfisoxazole	3CLPro	No	No	No	No
Dasatinib	3CLPro	No	Yes [16]	No	No
Dexamethasone	NSP15	Yes [17, 18]	Yes [19]	No	40 clinical trials
Phenolphthalein	NSP15	No	No	No	No
Spironolactone	NSP15	Yes [20]	Yes [21, 22, 23]	No	4 clinical trials
Mifepristone	NSP15	Yes [24]	No	No	No
Carbamazepine	NSP15	No	No	No	No
Vemurafenib	NSP12	No	Yes [25, 26]	No	No
Sorafenib	NSP12	No	Yes [27]	No	No
Levonorgestrel	NSP12	No	No	No	No
Naloxone	NSP12	No	No	No	No
Raloxifene	NSP12	No	No	No	No

Supplementary Table 10: Previous research analyzing the effects of our candidate drugs in SARS-CoV-2 infection.

2. References

[1] S. Abdulrab, S. Al-Maweri, E. Halboub, Ursodeoxycholic acid as a candidate therapeutic to alleviate and/or prevent COVID-19-associated cytokine storm, Med Hypotheses 143 (2020) 109897.

[2] A. Carino, F. Moraca, B. Fiorillo, S. Marchian?, V. Sepe, M. Biagioli, C. Finamore, S. Bozza, D. Francisci, E. Distrutti, B. Catalanotti, A. Zampella, S. Fiorucci, Hijacking SARS-CoV-2/ACE2 Receptor Interaction by Natural and Semi-synthetic Steroidal Agents Acting on Functional Pockets on the Receptor Binding Domain, Front Chem 8 (2020) 572885.

[3] M. T. Rehman, M. F. AlAjmi, A. Hussain, Natural Compounds as Inhibitors of SARS-CoV-2 Main Protease (3CLpro): A Molecular Docking and Simulation Approach to Combat COVID-19, Curr Pharm Des.

[4] M. Ye, G. Luo, D. Ye, M. She, N. Sun, Y. J. Lu, J. Zheng, Network pharmacology, molecular docking integrated surface plasmon resonance technology reveals the mechanism of Toujie Quwen Granules against coronavirus disease 2019 pneumonia, Phytomedicine (2020) 153401.

[5] M. Bello, A. Mart?nez-Mu?oz, I. Balbuena-Rebolledo, Identification of saquinavir as a potent inhibitor of dimeric SARS-CoV2 main protease through MM/GBSA, J Mol Model 26 (12) (2020) 340.

[6] T. Huynh, H. Wang, B. Luan, Structure-based lead optimization of herbal medicine rutin for inhibiting SARS-CoV-2's main protease, Phys Chem Chem Phys 22 (43) (2020) 25335–25343.

[7] H. A. Hassan, U. R. Abdelmohsen, O. M. Aly, S. Y. Desoukey, K. M. Mohamed, M. S. Kamel, Potential of Ficus microcarpa metabolites against SARS-CoV-2 main protease supported by docking studies, Nat Prod Res (2020) 1–5.

[8] F. M. A. da Silva, K. P. A. da Silva, L. P. M. de Oliveira, E. V. Costa, H. H. Koolen, M. L. B. Pinheiro, A. Q. L. de Souza, A. D. L. de Souza, Flavonoid glycosides and their putative human metabolites as potential inhibitors of the SARS-CoV-2 main protease (Mpro) and RNA-dependent RNA polymerase (RdRp), Mem Inst Oswaldo Cruz 115 (2020) e200207.

[9] S. C, D. K. S, V. Ragunathan, P. Tiwari, S. A, B. D. P, Molecular docking, validation, dynamics simulations, and pharmacokinetic prediction of natural compounds against the SARS-CoV-2 main-protease, J Biomol Struct Dyn (2020) 1–27.

[10] D. Bhowmik, R. Nandi, R. Jagadeesan, N. Kumar, A. Prakash, D. Kumar, Identification of potential inhibitors against SARS-CoV-2 by targeting proteins responsible for envelope formation and virion

assembly using docking based virtual screening, and pharmacokinetics approaches, Infect Genet Evol 84 (2020) 104451.

[11] P. Das, R. Majumder, M. Mandal, P. Basak, In-Silico approach for identification of effective and stable inhibitors for COVID-19 main protease (Mpro) from flavonoid based phytochemical constituents of Calendula officinalis, J Biomol Struct Dyn (2020) 1–16.

[12] R. Gomeni, T. Xu, X. Gao, F. Bressolle-Gomeni, Model based approach for estimating the dosage regimen of indomethacin a potential antiviral treatment of patients infected with SARS CoV-2, J Pharmacokinet Pharmacodyn 47 (3) (2020) 189–198.

[13] X. Zeng, X. Song, T. Ma, X. Pan, Y. Zhou, Y. Hou, Z. Zhang, K. Li, G. Karypis, F. Cheng, Repurpose Open Data to Discover Therapeutics for COVID-19 Using Deep Learning, J Proteome Res 19 (11) (2020) 4624–4636.

[14] M. A. Marinella, Indomethacin and resveratrol as potential treatment adjuncts for SARS-CoV-2/COVID-19, Int J Clin Pract 74 (9) (2020) e13535.

[15] C. Amici, A. Di Caro, A. Ciucci, L. Chiappa, C. Castilletti, V. Martella, N. Decaro, C. Buonavoglia, M. R. Capobianchi, M. G. Santoro, Indomethacin has a potent antiviral activity against SARS coronavirus, Antivir Ther 11 (8) (2006) 1021–1030.

[16] Z. Qiao, H. Zhang, H. F. Ji, Q. Chen, Computational View toward the Inhibition of SARS-CoV-2 Spike Glycoprotein and the 3CL Protease, Computation (Basel) 8 (2).

[17] R. Hajjo, D. A. Sabbah, S. K. Bardaweel, Chemocentric Informatics Analysis: Dexamethasone Versus Combination Therapy for COVID-19, ACS Omega 5 (46) (2020) 29765–29779.

[18] A. Alvarez, L. Cabia, C. Trigo, A. C. Bandr?s, M. Bestu?, Prescription profile in patients with SARS-CoV-2 infection hospitalised in Aragon, Spain, Eur J Hosp Pharm.

[19] I. Sarkar, A. Sen, In silico screening predicts common cold drug Dextromethorphan along with Prednisolone and Dexamethasone can be effective against novel Coronavirus disease (COVID-19), J Biomol Struct Dyn (2020) 1–5.

[20] V. Y. Mareev, Y. A. Orlova, E. P. Pavlikova, S. T. Matskeplishvili, Z. A. Akopyan, A. G. Plisyk, E. M. Seredenina, D. A. Asratyan, A. V. Potapenko, P. S. Malakhov, L. M. Samokhodskaya, E. A. Mershina, V. E. Sinitsyn, M. M. Bulanova, A. A. Fuks, Y. V. Mareev, Y. L. Begrambekova, A. A. Kamalov, [Combination therapy at an early stage of the novel coronavirus infection (COVID-19). Case series

and design of the clinical trial "BromhexIne and Spironolactone for CoronavirUs Infection requiring hospiTalization (BISCUIT)"], Kardiologiia 60 (8) (2020) 4–15.

[21] G. A. Pathak, F. R. Wendt, A. Goswami, F. De Angelis, R. Polimanti, ACE2 Netlas: In-silico functional characterization and drug-gene interactions of ACE2 gene network to understand its potential involvement in COVID-19 susceptibility, medRxiv.

[22] F. A. Cadegiani, Repurposing existing drugs for COVID-19: an endocrinology perspective, BMC Endocr Disord 20 (1) (2020) 149.

[23] F. A. Cadegiani, A. Goren, C. G. Wambier, Spironolactone may provide protection from SARS-CoV-2: Targeting androgens, angiotensin converting enzyme 2 (ACE2), and renin-angiotensin-aldosterone system (RAAS), Med Hypotheses 143 (2020) 110112.

[24] S. Matsuyama, M. Kawase, N. Nao, K. Shirato, M. Ujike, W. Kamitani, M. Shimojima, S. Fukushi, The inhaled steroid ciclesonide blocks SARS-CoV-2 RNA replication by targeting the viral replication-transcription complex in cultured cells, J Virol.

[25] A. Ianevski, R. Yao, S. Biza, E. Zusinaite, A. Mannik, G. Kivi, A. Planken, K. Kurg, E. M. Tombak, M. Ustav, N. Shtaida, E. Kulesskiy, E. Jo, J. Yang, H. Lysvand, K. L?seth, V. Oksenych, P. A. Aas, T. Tenson, A. Vitkauskien?, M. P. Windisch, M. H. Fenstad, S. A. Nordb?, M. Ustav, M. Bj?r?s, D. E. Kainov, Identification and Tracking of Antiviral Drug Combinations, Viruses 12 (10).

[26] S. Sinha, K. Cheng, A. A. Sch?ffer, K. Aldape, E. Schiff, E. Ruppin, In vitro and in vivo identification of clinically approved drugs that modify ACE2 expression, Mol Syst Biol 16 (7) (2020) e9628.

[27] Y. H. Taguchi, T. Turki, A new advanced in silico drug discovery method for novel coronavirus (SARS-CoV-2) with tensor decomposition-based unsupervised feature extraction, PLoS One 15 (9) (2020) e0238907.
