# Supplementary material for: A COVID-19 Drug Repurposing Strategy through Quantitative Homological Similarities Using a Topological Data Analysis-Based Framework
Source: Pharmaceutics. 2021 Apr 2;13(4):488. doi: 10.3390/pharmaceutics13040488 (PMC8066156; doi:10.3390/pharmaceutics13040488)

SUPPLEMENTARY FILE 4

Interacting residues between the 3 viral proteins and the 16 drugs identified as potential repurposing candidates.

6m2q: 3CL pro

DB00263: Sulfisoxazole

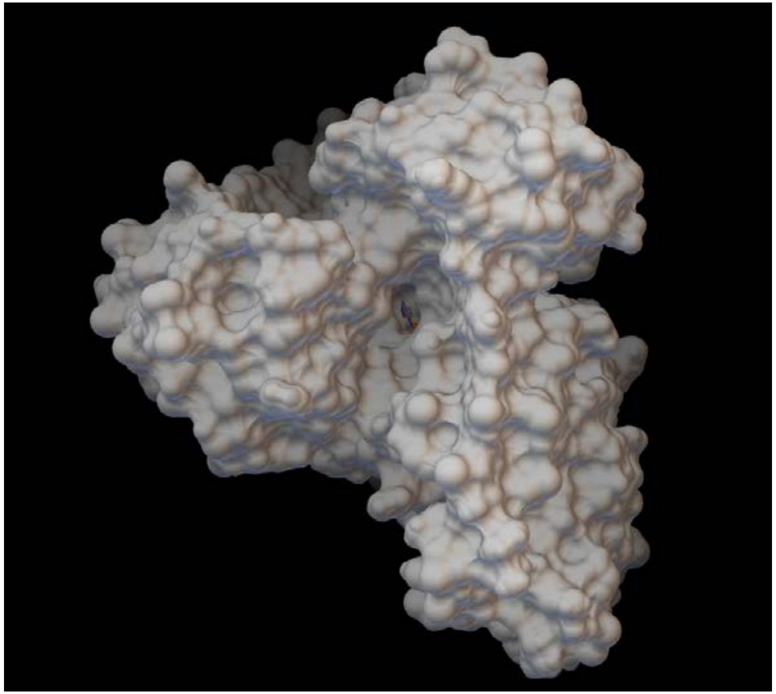

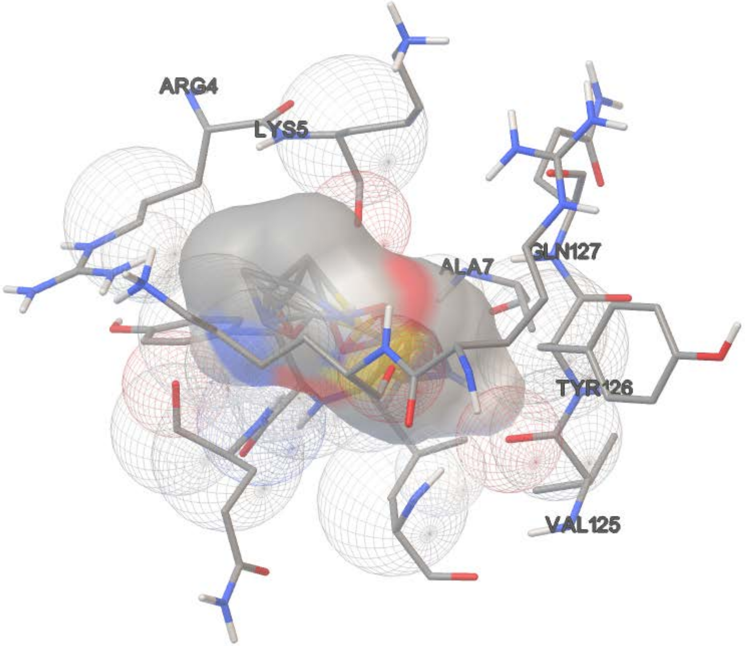

6m2q: 3CL pro

DB00328: Indomethacin

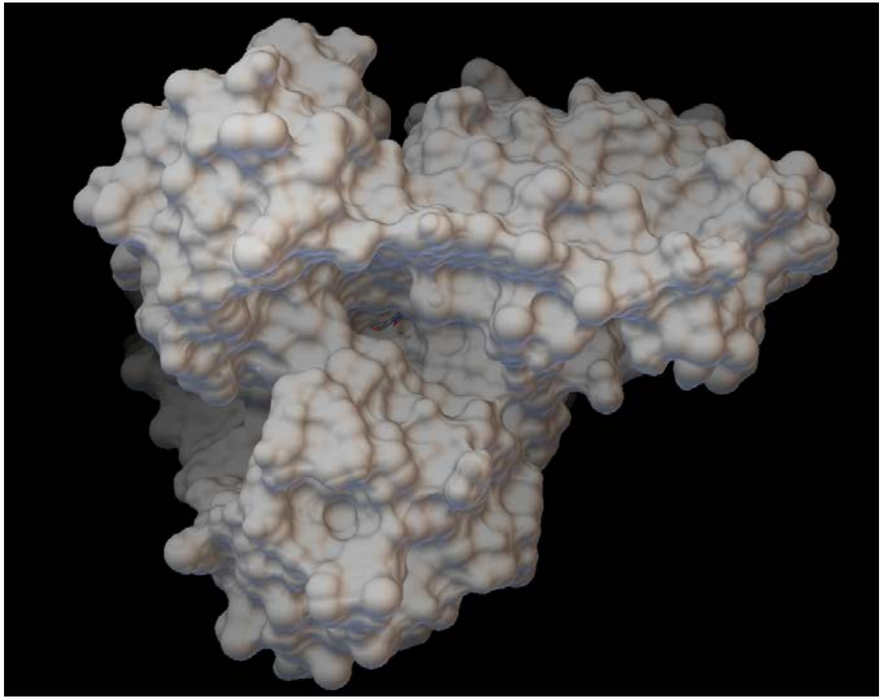

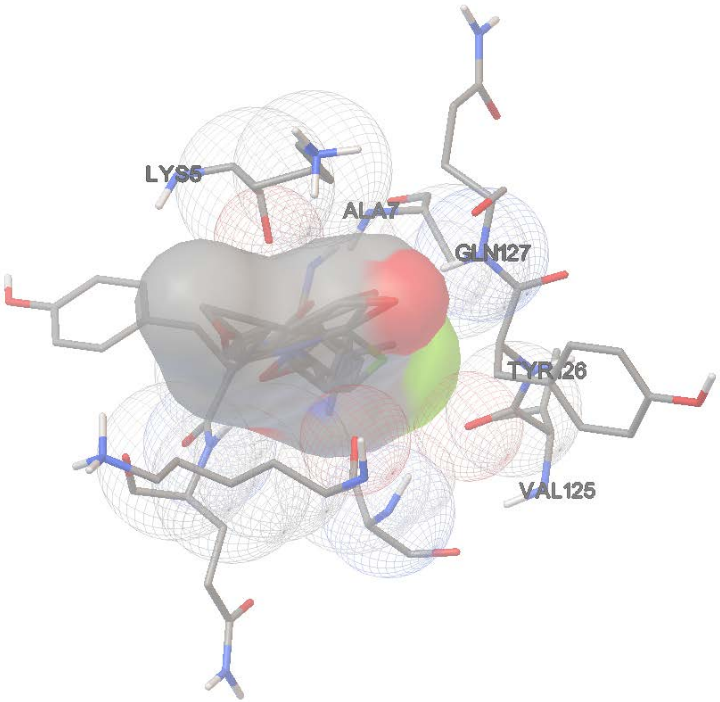

6m2q: 3CL pro

DB00605: Sulindac

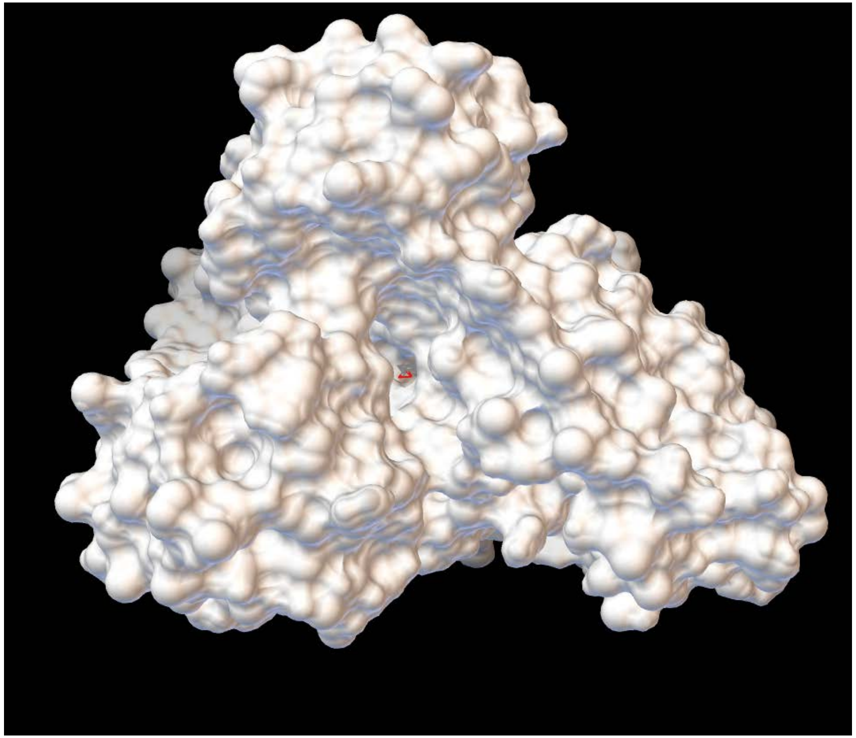

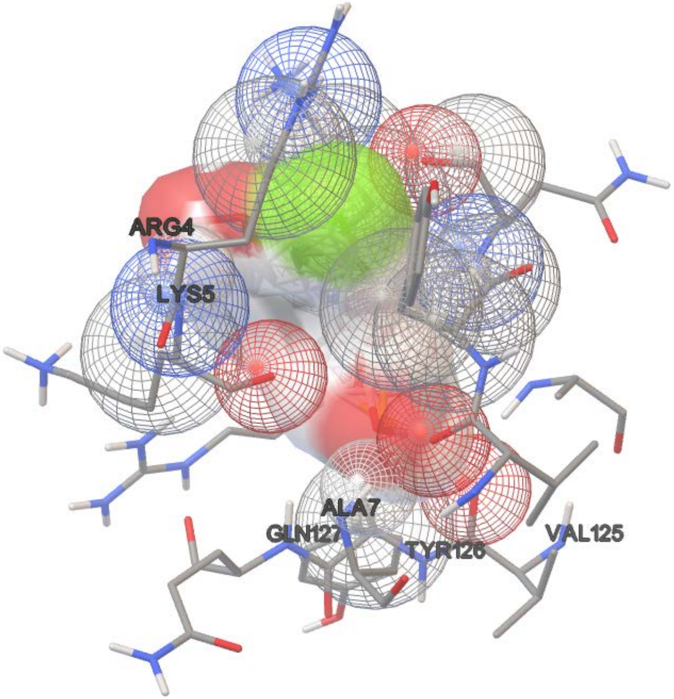

6m2q: 3CL pro

DB01254: Dasatinib

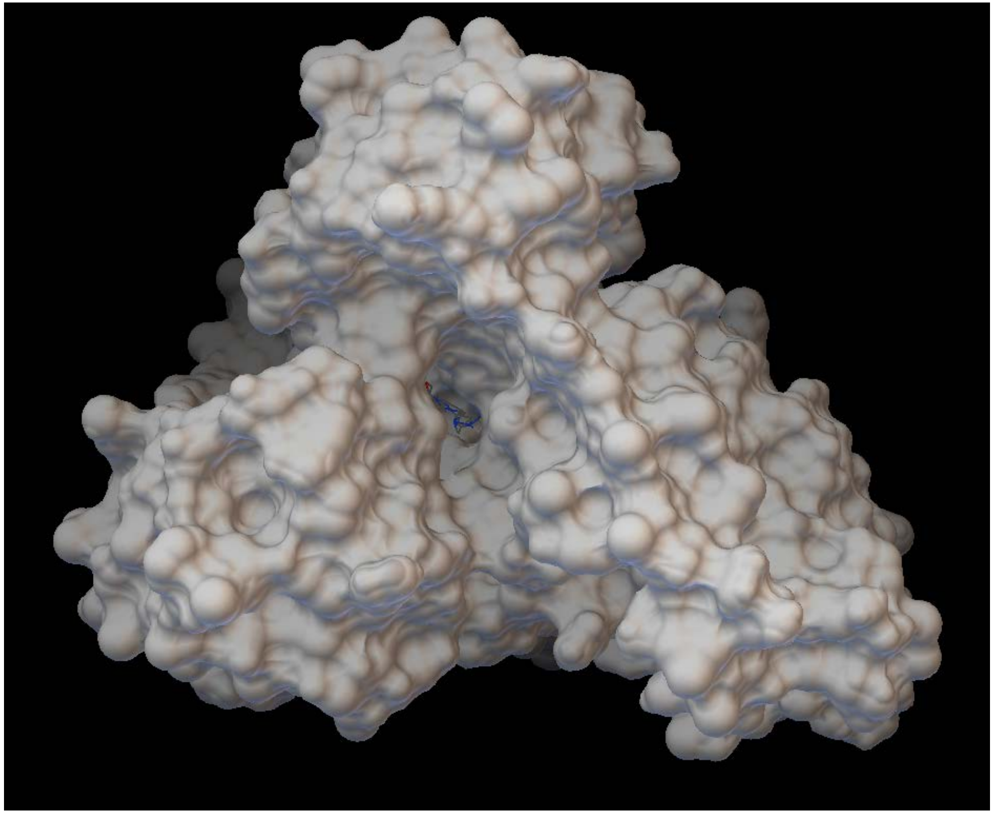

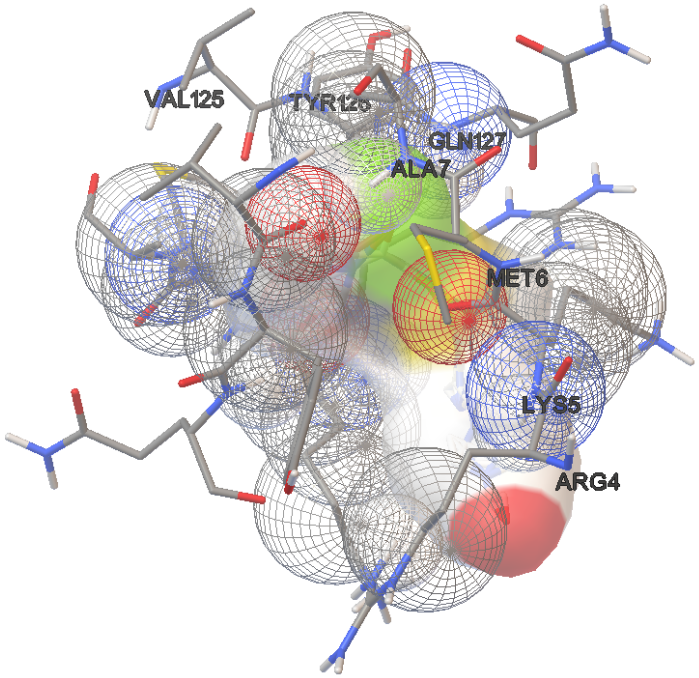

6m2q: 3CL pro

DB01698: Rutin

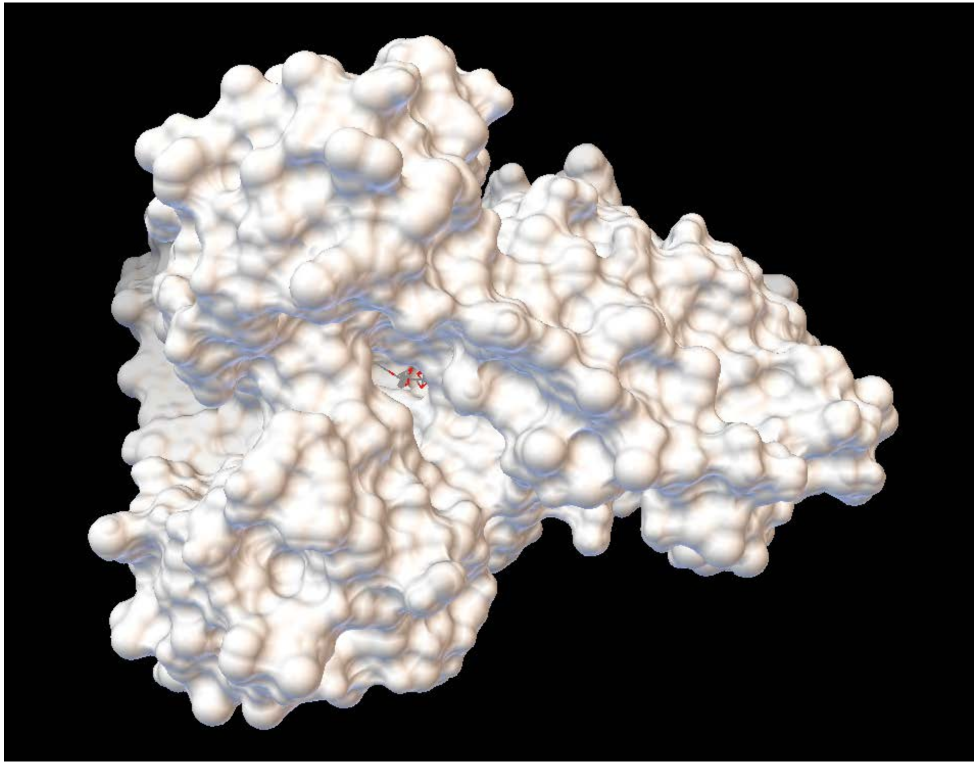

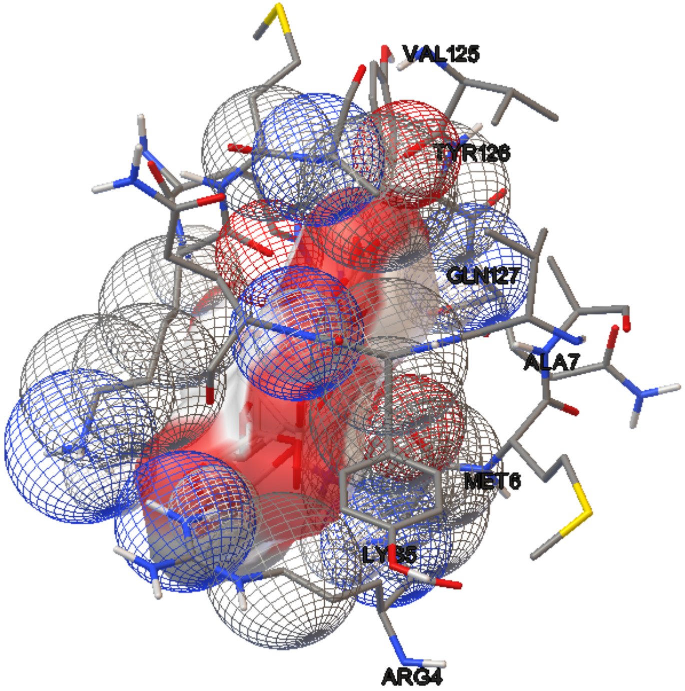

6m2q: 3CL pro

DB02659: Cholic Acid

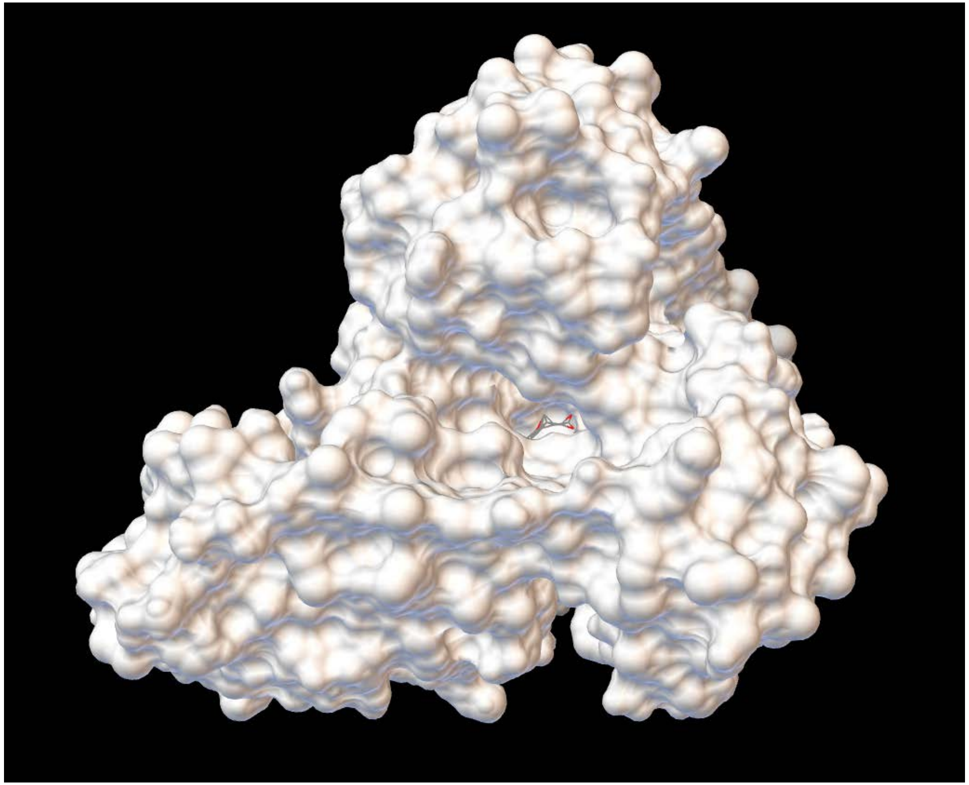

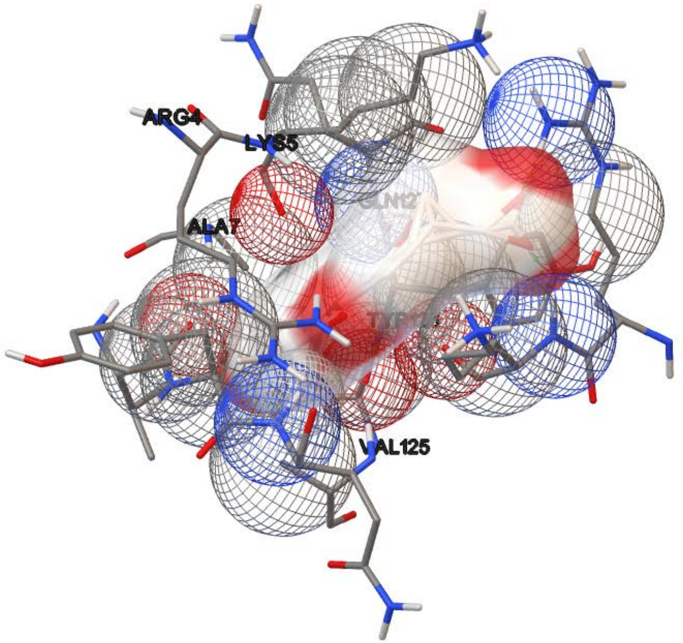

6m71: RNA-dependent RNA pol

DB00367: Levonorgestrel

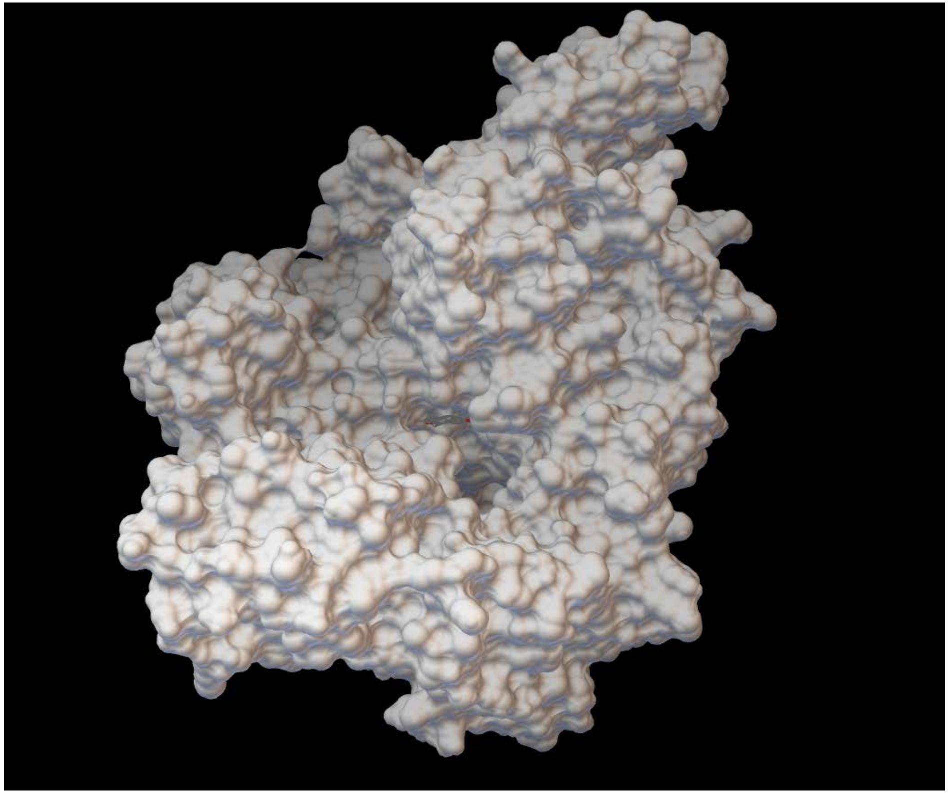

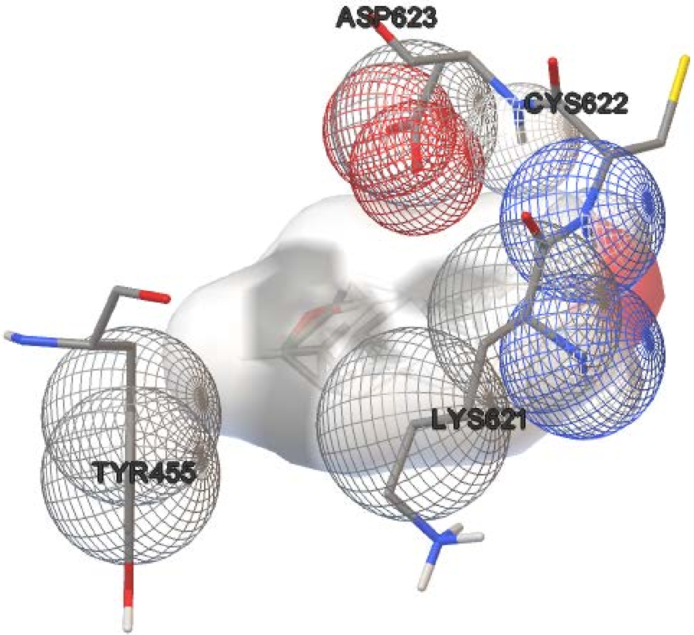

6m71: RNA-dependent RNA pol

DB00398: Sorafenib

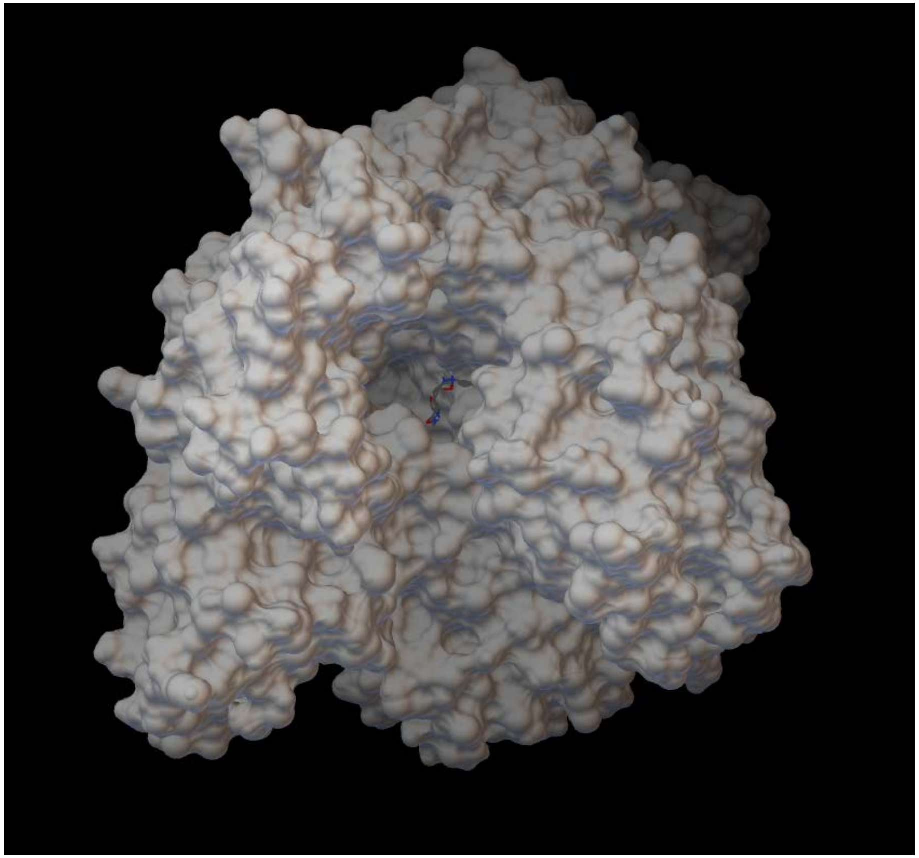

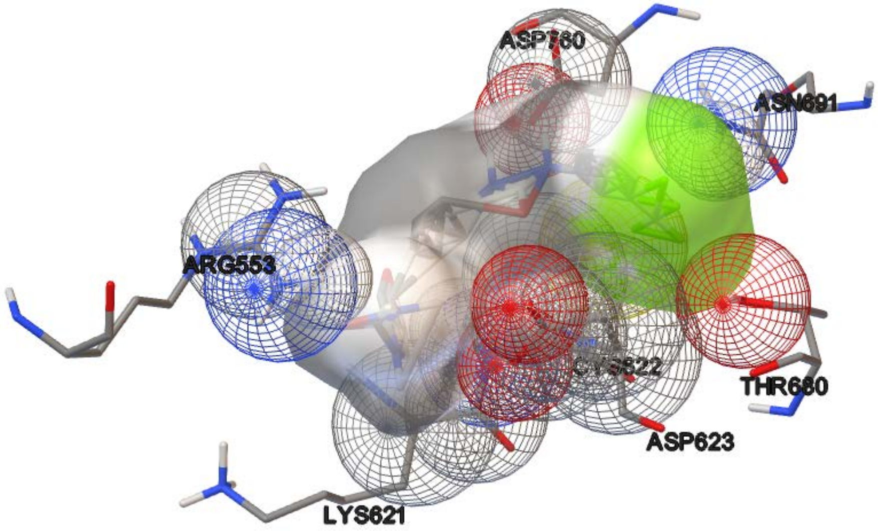

6m71: RNA-dependent RNA pol

DB00481: Raloxifene

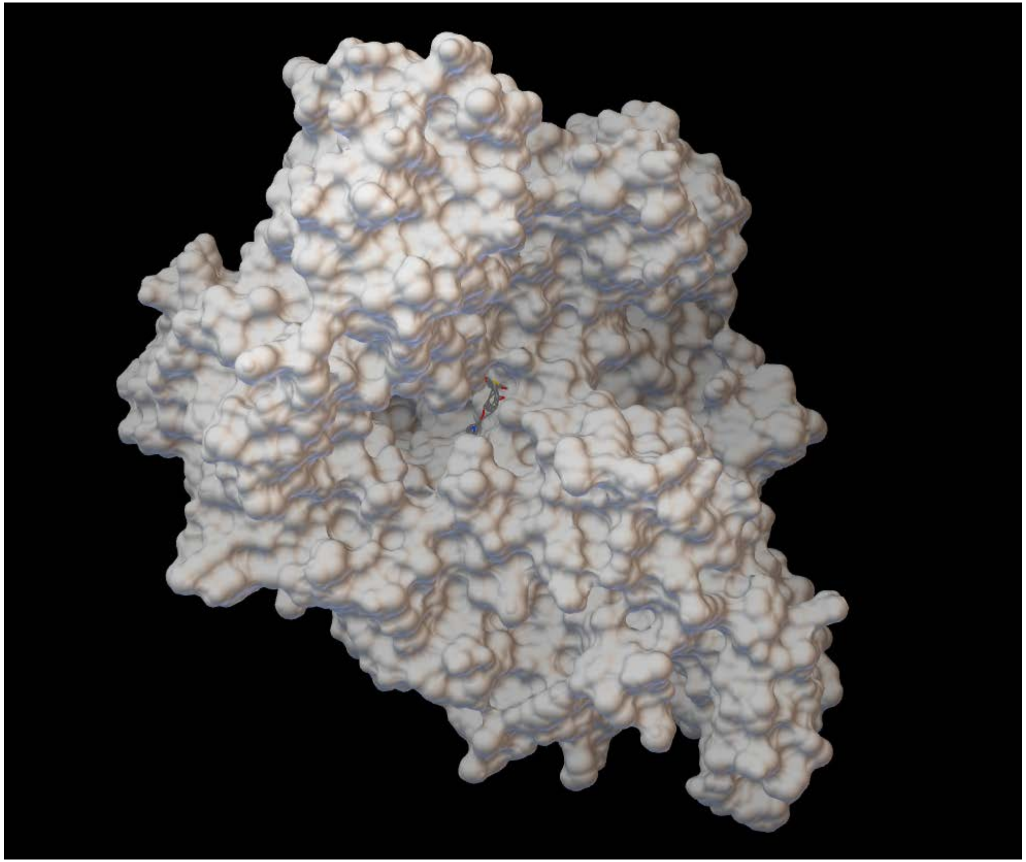

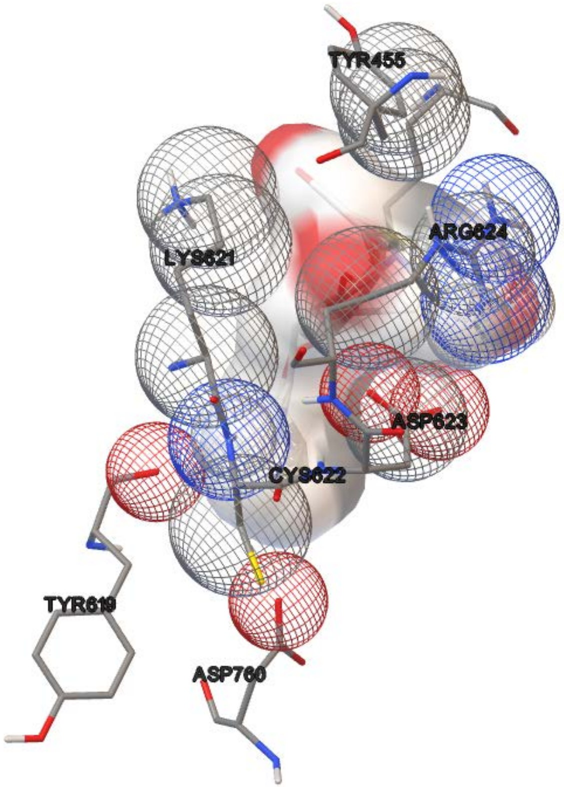

6m71: RNA-dependent RNA pol

DB01183: Naloxone

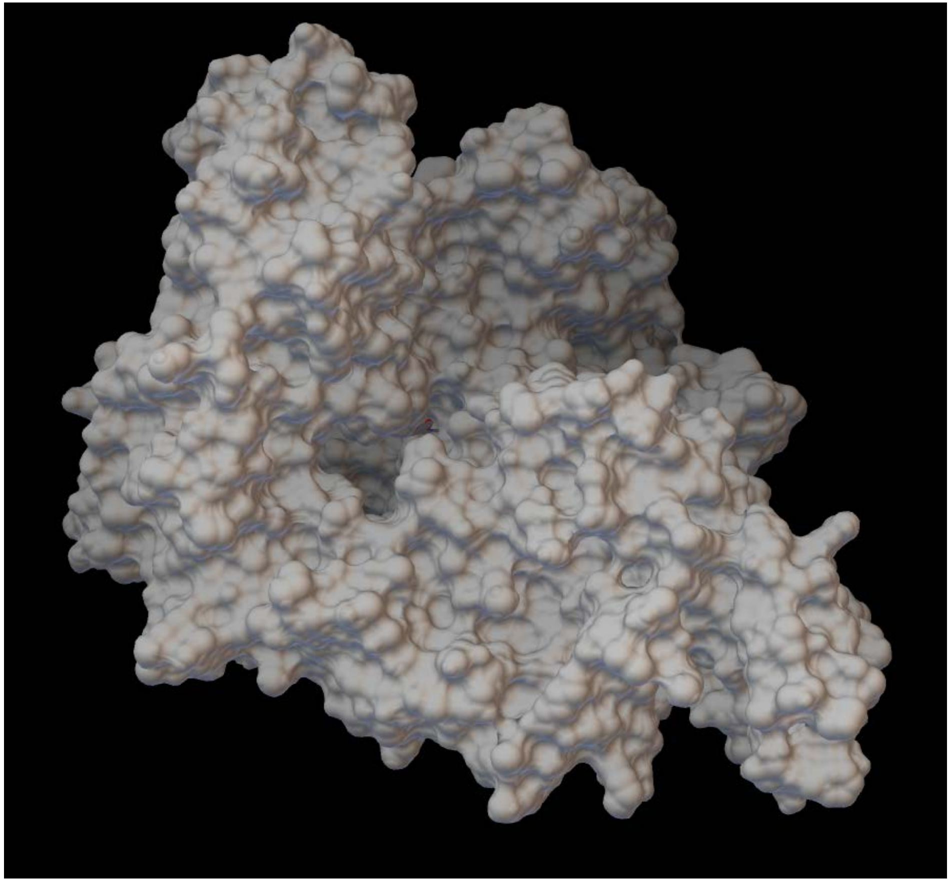

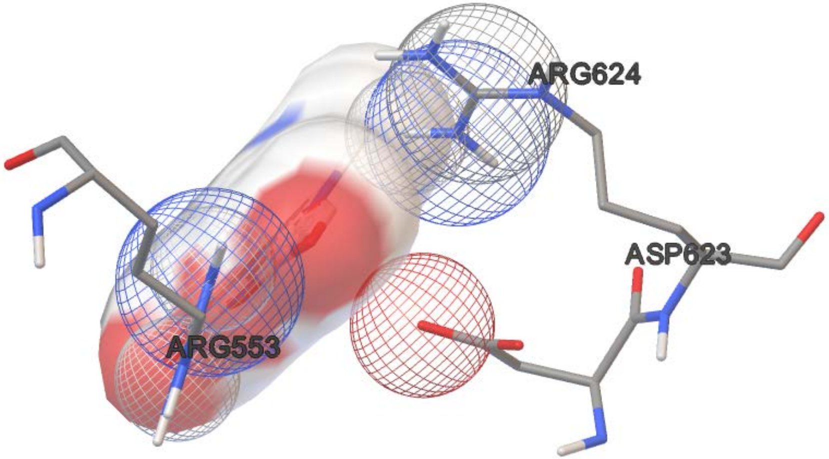

6m71: RNA-dependent RNA pol

DB08881: Vemurafenib

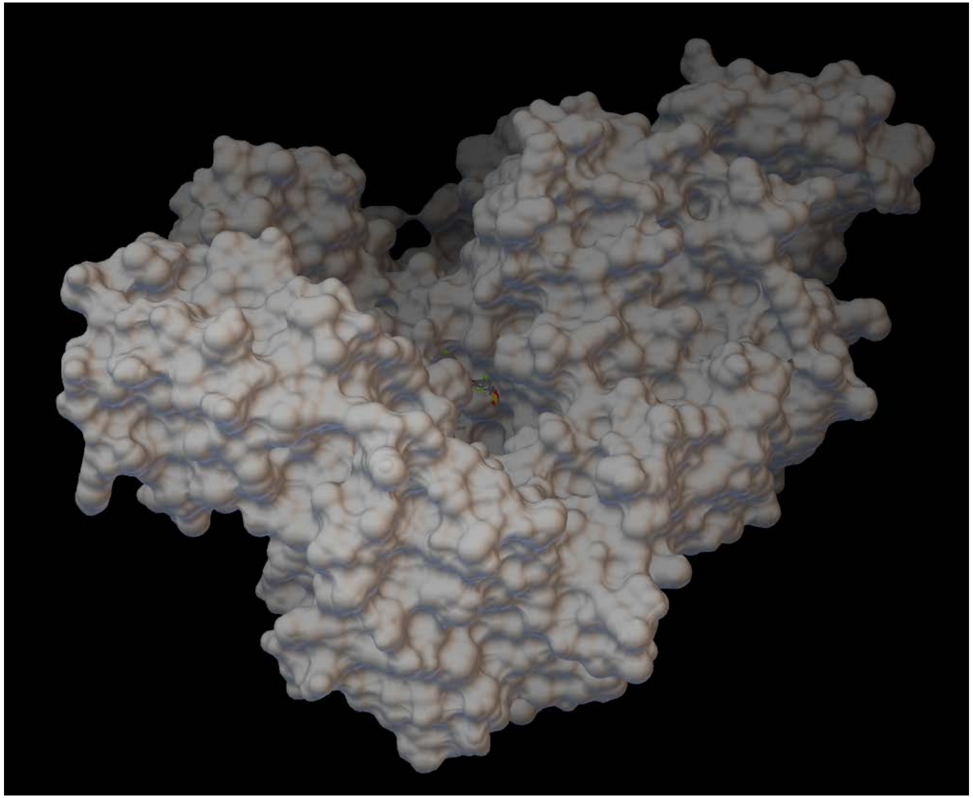

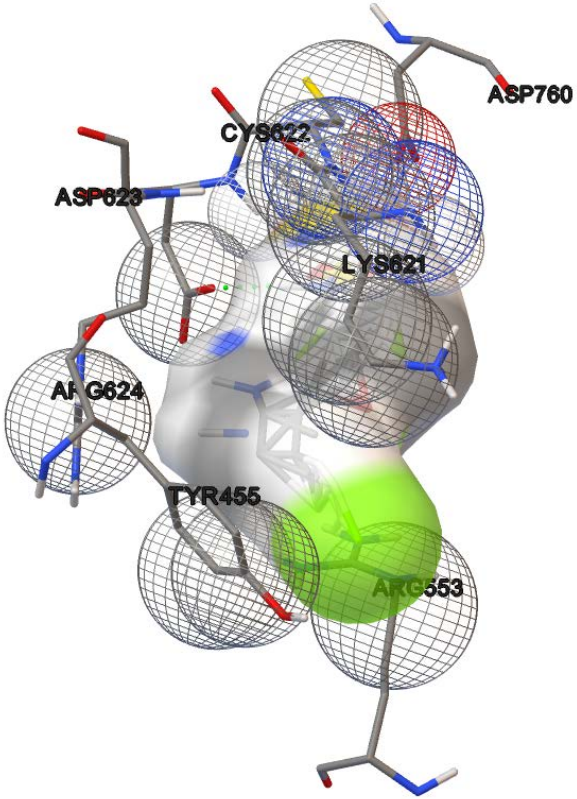

6w01: NSP15 endoribonuclease

DB00421: Spironolactone

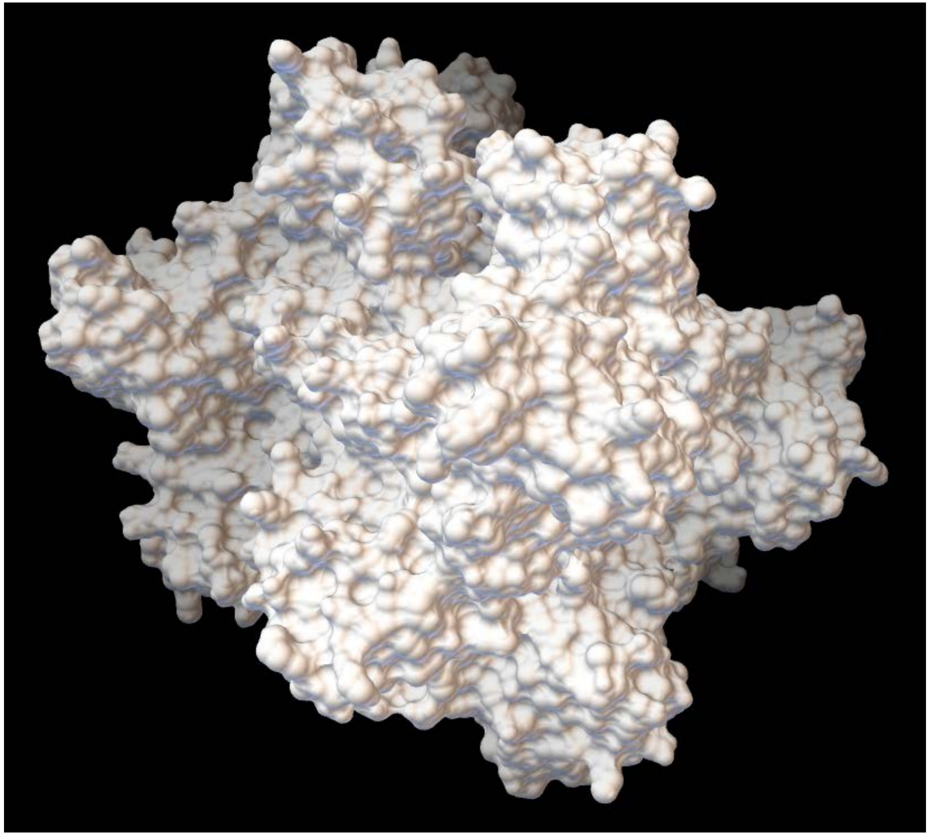

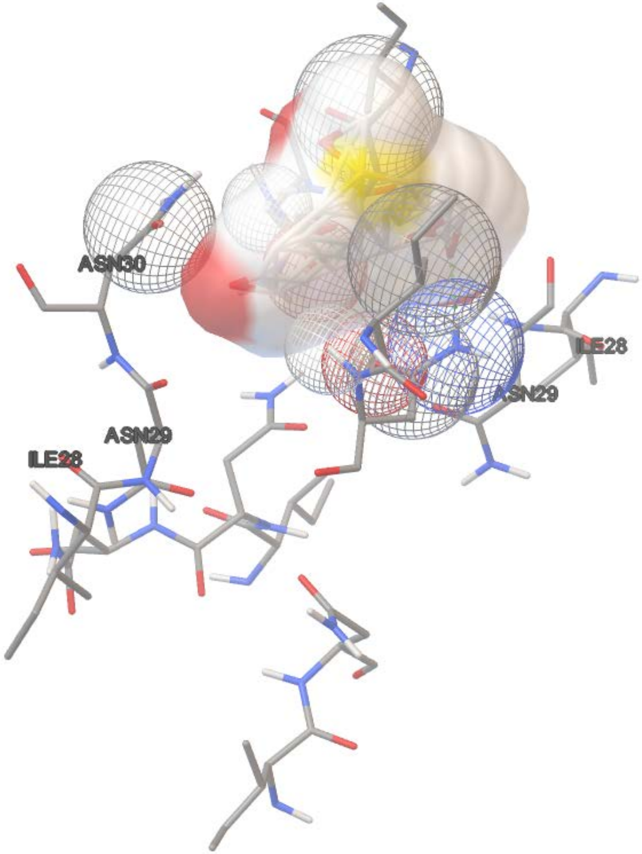

6w01: NSP15 endoribonuclease

DB00564: Carbamazepine

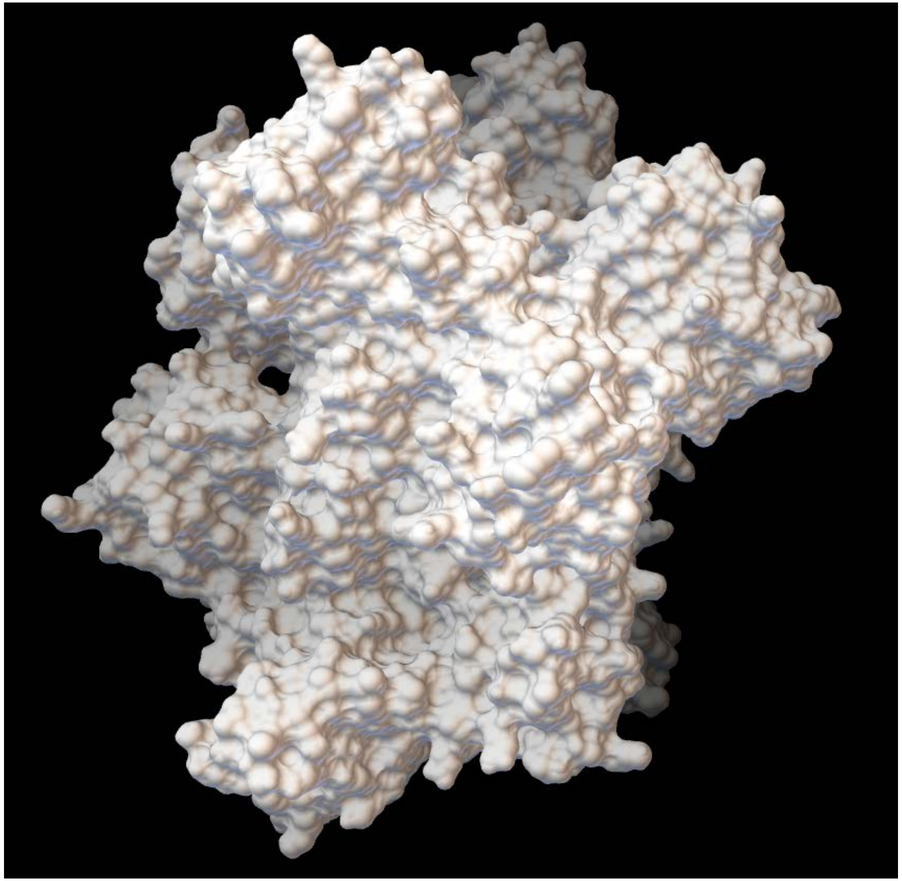

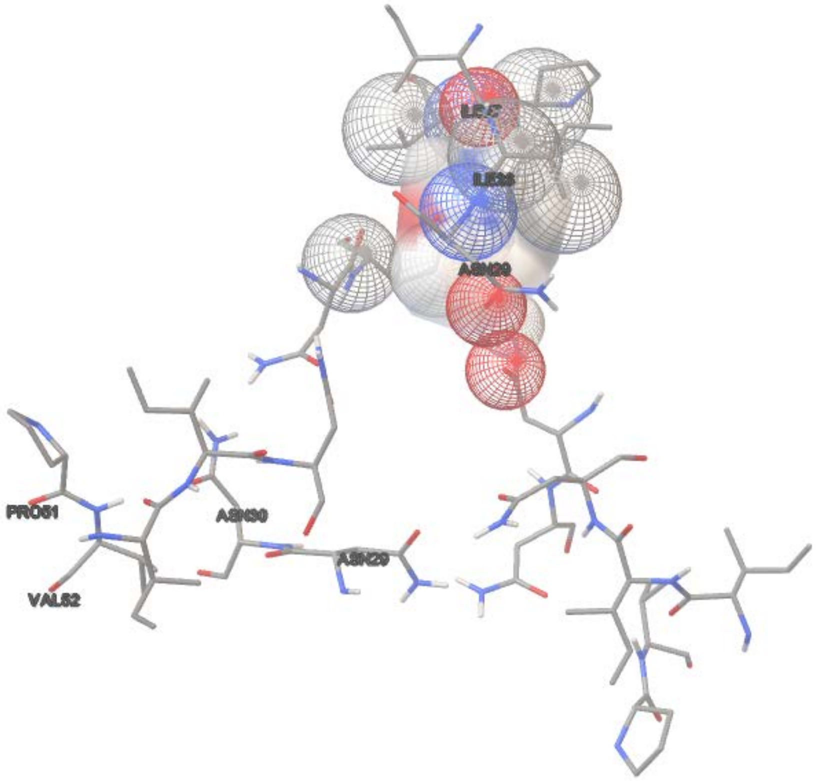

6w01: NSP15 endoribonuclease

DB00834:Mifepristone

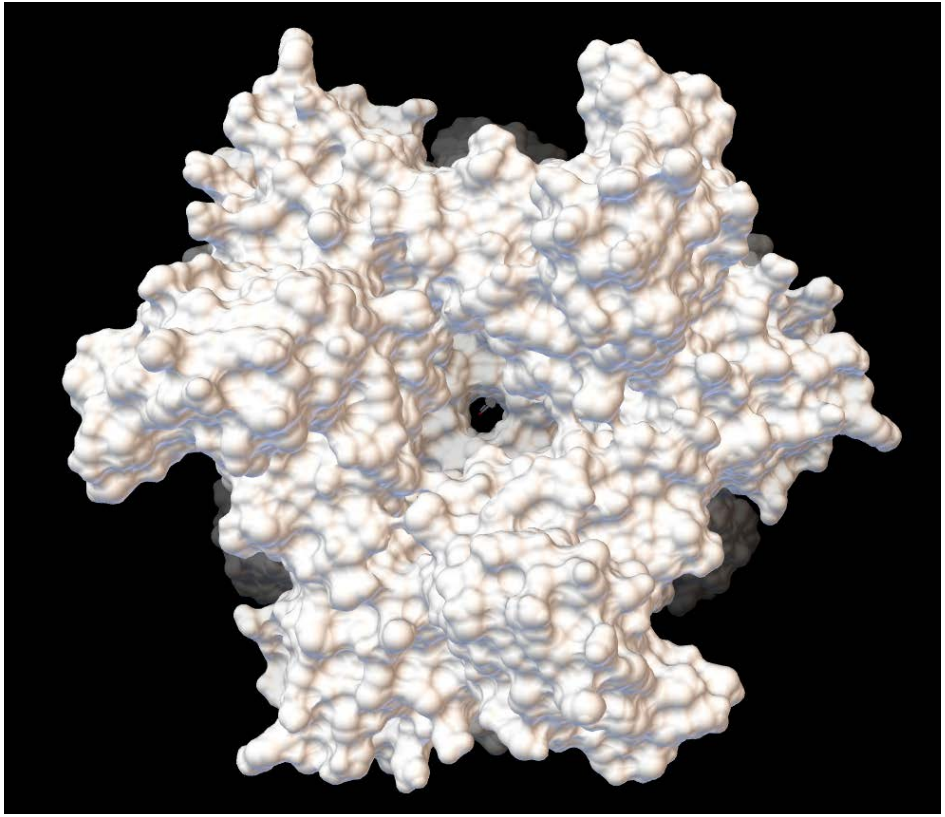

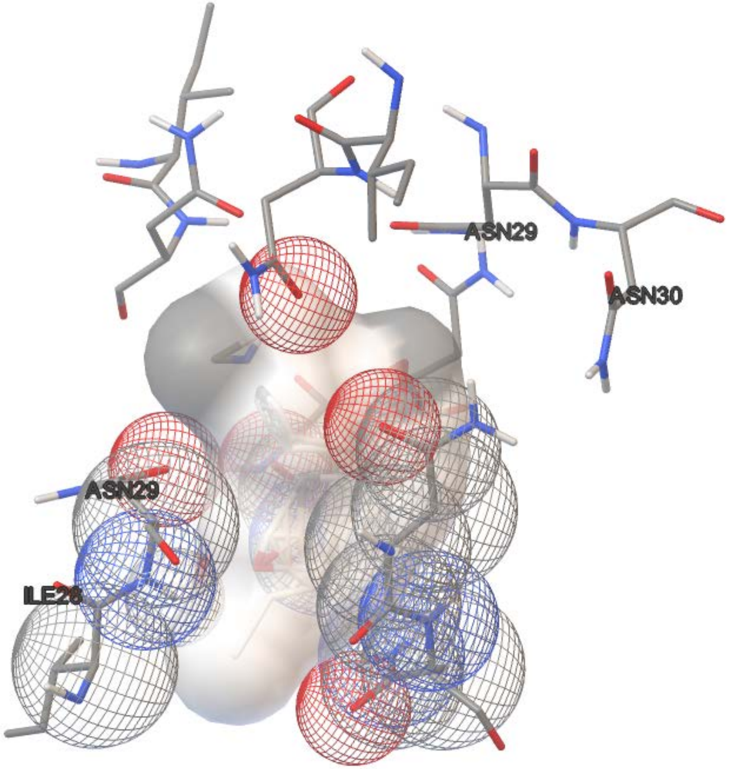

6w01: NSP15 endoribonuclease

DB01234: Dexamethasone

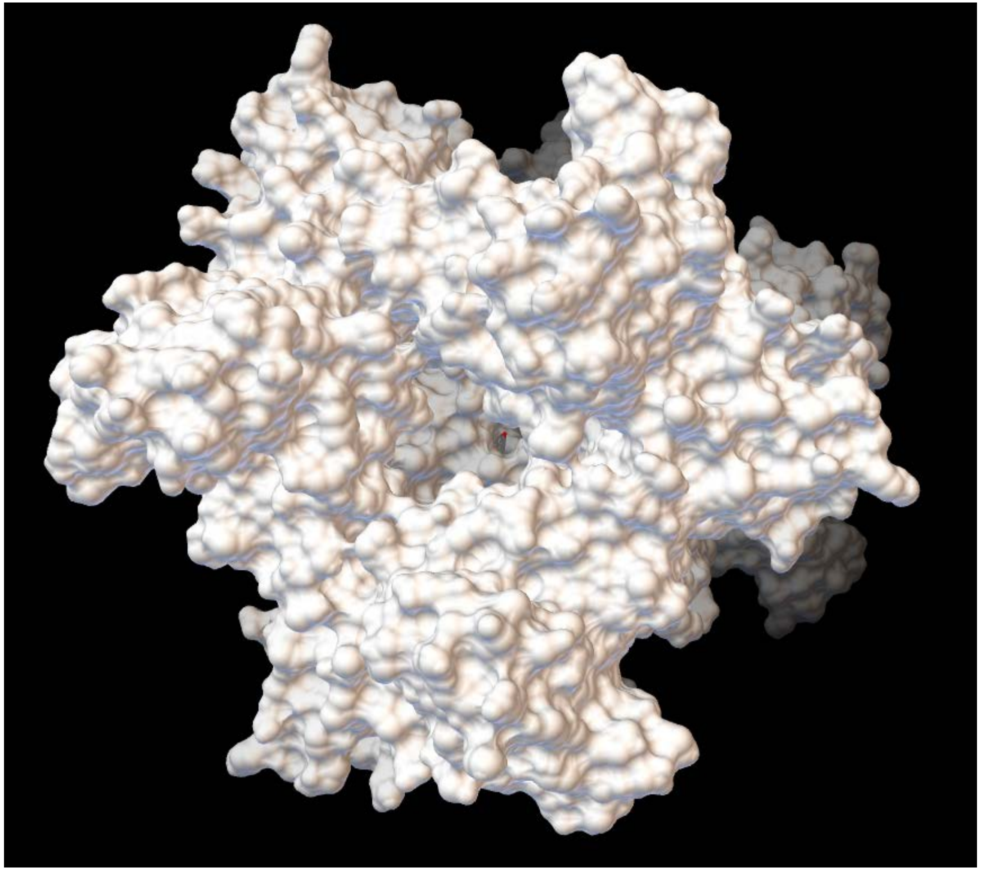

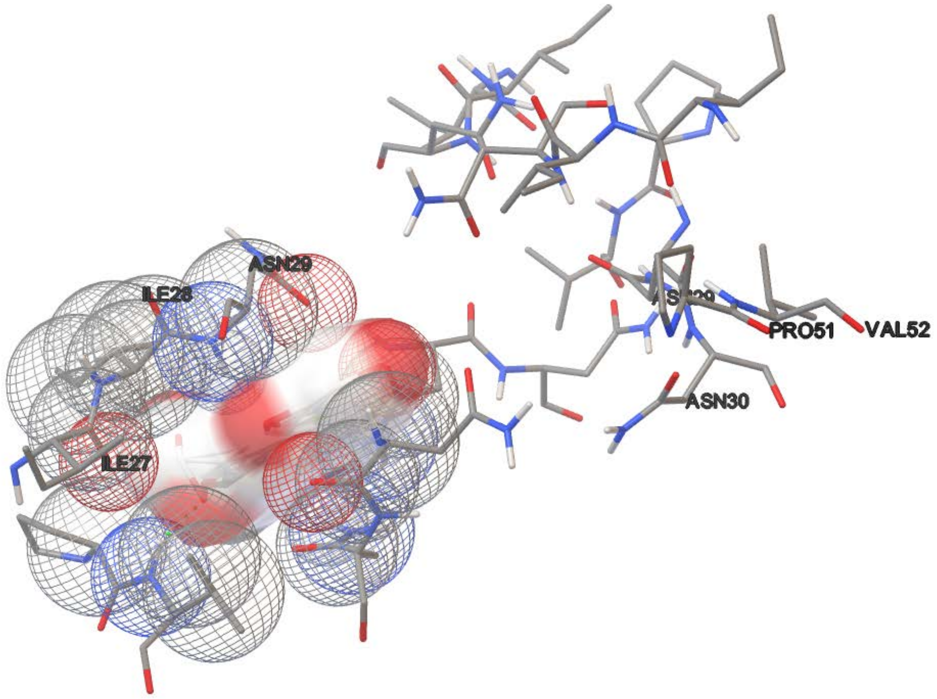

6w01: NSP15 endoribonuclease

DB04824: Phenolphthelein

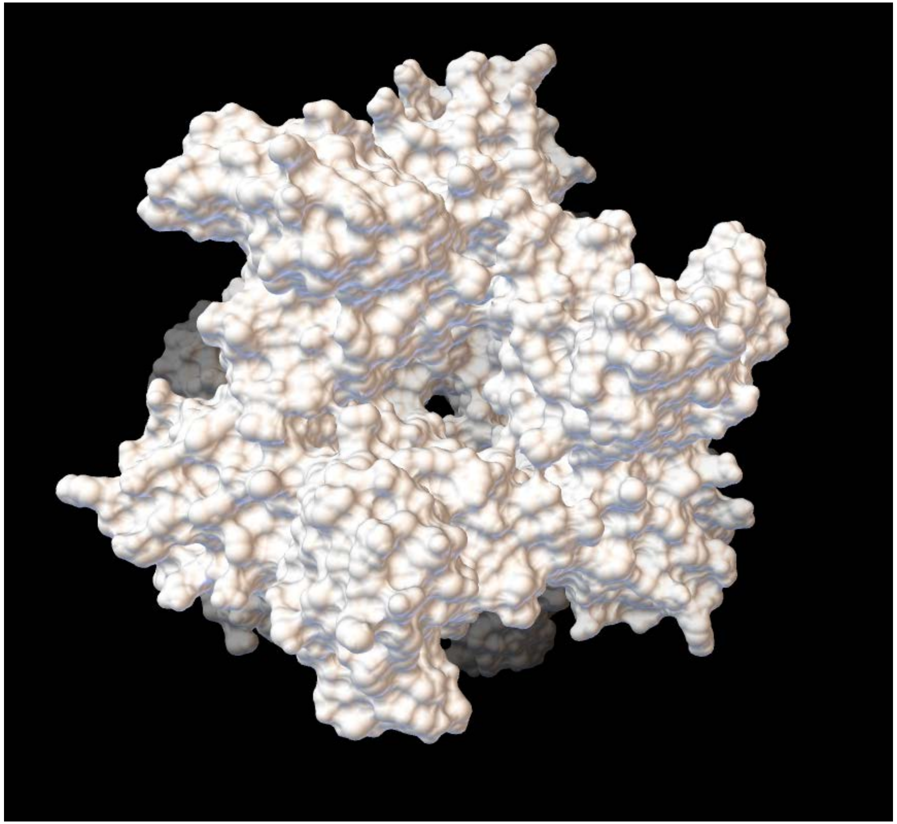

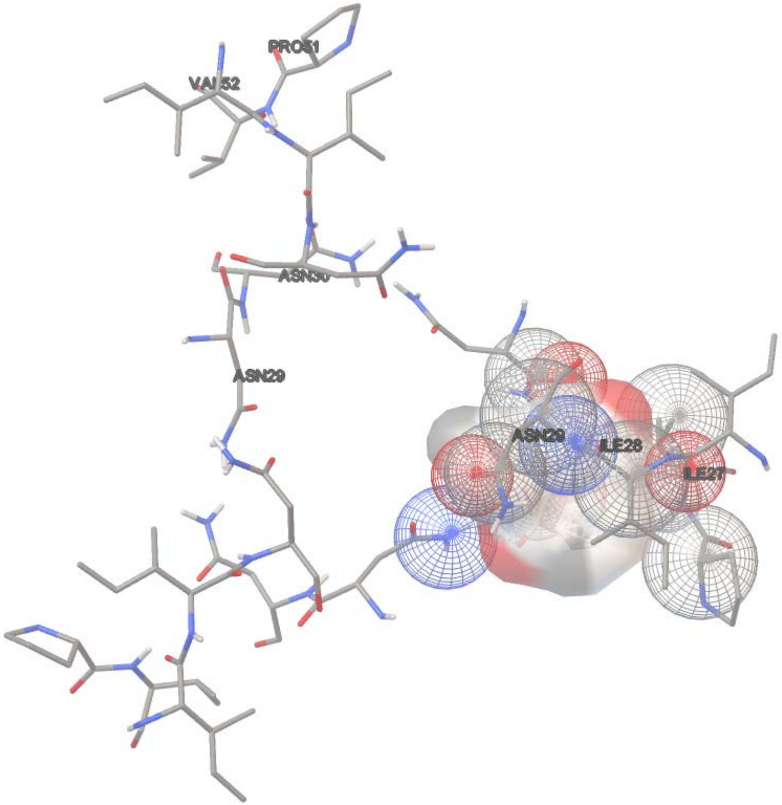

Supplement: Supplementary file 1 [file pharmaceutics-13-00488-s001.zip › SupplementaryFile4.pdf]
